# Profiling Genome-Wide DNA Methylation Patterns in Human Aortic and Mitral Valves

**DOI:** 10.3389/fcvm.2022.840647

**Published:** 2022-04-06

**Authors:** Sarah Halawa, Najma Latif, Yuan-Tsan Tseng, Ayman M. Ibrahim, Adrian H. Chester, Ahmed Moustafa, Yasmine Aguib, Magdi H. Yacoub

**Affiliations:** ^1^Aswan Heart Centre, Aswan, Egypt; ^2^Biotechnology Graduate Program, American University in Cairo, New Cairo, Egypt; ^3^Heart Science Centre, Magdi Yacoub Institute, Harefield, United Kingdom; ^4^National Heart and Lung Institute (NHLI), Imperial College London, London, United Kingdom; ^5^Zoology Department, Faculty of Science, Cairo University, Giza, Egypt; ^6^Department of Biology, American University in Cairo, New Cairo, Egypt

**Keywords:** epigenetics, heart valves, NOTCH signaling, extracellular matrix (ECM), endothelial mesenchymal trans-differentiation (EMT), HIF-1 signaling pathway, regulation of actin cytoskeleton, promoters

## Abstract

Cardiac valves exhibit highly complex structures and specialized functions that include dynamic interactions between cells, extracellular matrix (ECM) and their hemodynamic environment. Valvular gene expression is tightly regulated by a variety of mechanisms including epigenetic factors such as histone modifications, RNA-based mechanisms and DNA methylation. To date, methylation fingerprints of non-diseased human aortic and mitral valves have not been studied. In this work we analyzed the differential methylation profiles of 12 non-diseased aortic and mitral valve tissue samples (in matched pairs). Analysis of methylation data [reduced representation bisulfite sequencing (RRBS)] of 16,101 promoters genome-wide revealed 584 differentially methylated (DM) promoters, of which 13 were reported in endothelial mesenchymal trans-differentiation (EMT), 37 in aortic and mitral valve disease and 7 in ECM remodeling. Both functional classification as well as network analysis showed that the genes associated with the DM promoters were enriched for WNT-, Cadherin-, Endothelin-, PDGF-, HIF-1 and VEGF- signaling implicated in valvular physiology and pathophysiology. Additional enrichment was detected for TGFB-, NOTCH- and Integrin- signaling involved in EMT as well as ECM remodeling. This data provides the first insight into differential regulation of human aortic and mitral valve tissue and identifies candidate genes linked to DM promoters. Our work will improve the understanding of valve biology, valve tissue engineering approaches and contributes to the identification of relevant drug targets.

## Introduction

Heart valves perform a range of sophisticated functions that ensure unidirectional blood flow during systole, prevent backflow during diastole, enhance coronary blood flow and maintain left ventricular as well as myocardial function (1). These functions are sustained throughout the human's lifetime and require tight regulation of the valve cells and extracellular matrix (ECM), which continuously interact together enabling the valves to actively adapt to their complex hemodynamic and biomechanical environments (1, 2).

The mature valves consist of valve interstitial cells (VICs) populating a central layered ECM (3). The specific functions and hemodynamic environment of the mitral and the aortic valve (4, 5) could require distinct gene regulation by epigenetics including DNA methylation.

Epigenetics refers to heritable phenotype changes that do not involve changes in the DNA sequence itself and includes mechanisms such as histone modifications, RNA-based mechanisms and DNA methylation (6). DNA methylation is a process by which a methyl group is added to the 5^th^ carbon of cytosine, which alters the structure of the DNA molecule thus allowing differential regulation of gene expression either through obstructing transcription factor (TF) binding or through the recruitment of methyl-binding proteins, which bind complexes responsible for chromatin remodeling (7). This process is heritable as well as tissue-specific and plays a major role in various physiological processes linked to cardiogenesis such as cardiomyocyte development, maturation and cardiac regeneration (8, 9). Aberrant changes in methylation profiles are associated with cardiovascular diseases (CVDs) such as ventricular septal defects, tetralogy of Fallot, atherosclerosis as well as other CVDs that lead to end-stage heart failure (10–13).

We here provide an initial insight into differential methylation profiles of non-diseased heart valves.

## Materials and Methods

### Ethics Statement and Study Cohort/Samples

This study was approved by the Royal Brompton hospital ethics review board / Brompton and Harefield trust ethics committee (REC approval 10/H0724/18) and is abiding by all the standards of the Declaration of Helsinki. Written informed consent was obtained from the donors prior to their inclusion in the study. Twelve non-diseased valves free from calcification (6 aortic and 6 mitral valves; 10 males: 2 females; age range 42–64 years, mean age 52.2 years, SD 9.9682) were used in this study. After applying inclusion/exclusion criteria three of the twelve valves were excluded from the downstream analysis (Table 1, Supplementary Figure 1). The non-diseased valves were obtained from unused valves of healthy donor hearts, who died of non-cardiac diseases (Table 1). History, macroscopic, and microscopic evaluation were additionally performed to make sure that the donor hearts chosen are free from cardiovascular and valvular complications. The exclusion criteria of donor hearts were previously described in (14).

**Table 1:** Cohort demographic and clinical characteristics.

**Donor no**.	**Gender**	**Age**	**Cause of death**	**Aortic sample label**	**Mitral sample label**
1.	Male	62	Intracerebral hemorrhage	G1T^*^	G2T^*^
2.	Male	44	Transplant recipient	G3T^*^	G4T
3.	Male	44	Transplant recipient	G5T	G6T
4.	Male	42	Intracranial thrombosis	G7T	G8T
5.	Male	57	Intracerebral hemorrhage	G9T	G10T
6.	Female	64	Intracerebral hemorrhage	G11T	G12T

* *Removed from dataset*.

### Tissue Sampling and DNA Extraction

Aortic and mitral valves from donor hearts were provided by the Royal Brompton Valve biobank. The ischemic time for fresh tissue harvest was set to not exceed 24 h. The aortic and mitral valve leaflets were excised and separately deendothelialized using collagenase II for 10 min at 37°C as the focus of the study was the interstitial cell population. The leaflets were then washed using PBS, snap frozen and stored in −80°C for DNA extraction. DNA was isolated from deendothelialized tissue using the FitAmp™ Blood and Cultured Cell DNA Extraction Kit (Epigentek, NY, USA, catalog #: P-1018) and was subsequently eluted in TE buffer in a total volume of 40 μl. DNA was finally quantified and quality controlled *via* Qubit fluorescence.

### Bisulfite Conversion, Library Preparation and Sequencing

For each sample, 300 ng of DNA was digested for 2 h with the MSP1 enzyme (20U/sample at 37°C) followed by 2 h with TaqαI (20U/sample at 65°C). Digested, CGI enriched DNA fragments <300 bps in length, were selected for using MQ Binding Beads and subsequently collected for bisulfite treatment. Bisulfite treatment was performed using the Methylamp DNA Bisulfite Conversion Kit (Epigentek, NY, USA, catalog #: P-1001). Bisulfite conversion efficiency of the bisulfite-treated DNA was determined by RT-PCR using two pairs of primers against bisulfite-converted DNA (b-actin) and against unconverted DNA (GAPDH), for the same bisulfite-treated DNA samples. Conversion was deemed successful, if more than 99% of the DNA were converted (Epigentek, NY, USA).

Post-bisulfite PBAT-mediated library preparation was performed using the P-1056A kit. First, DNA end polishing and adaptor ligation was performed. This was followed by library amplification using indexed primers and library purification. The final purified library was eluted in 12 μl of water. Assessment of library quality was done *via* bioanalyzer and KAPA library quantification (Roche, CA, USA). Finally, 10 nM of sample libraries were subjected to single-end enhanced RRBS on Illumina HiSeq 2500.

### Data Quality Control and Processing

**Figure 1:**
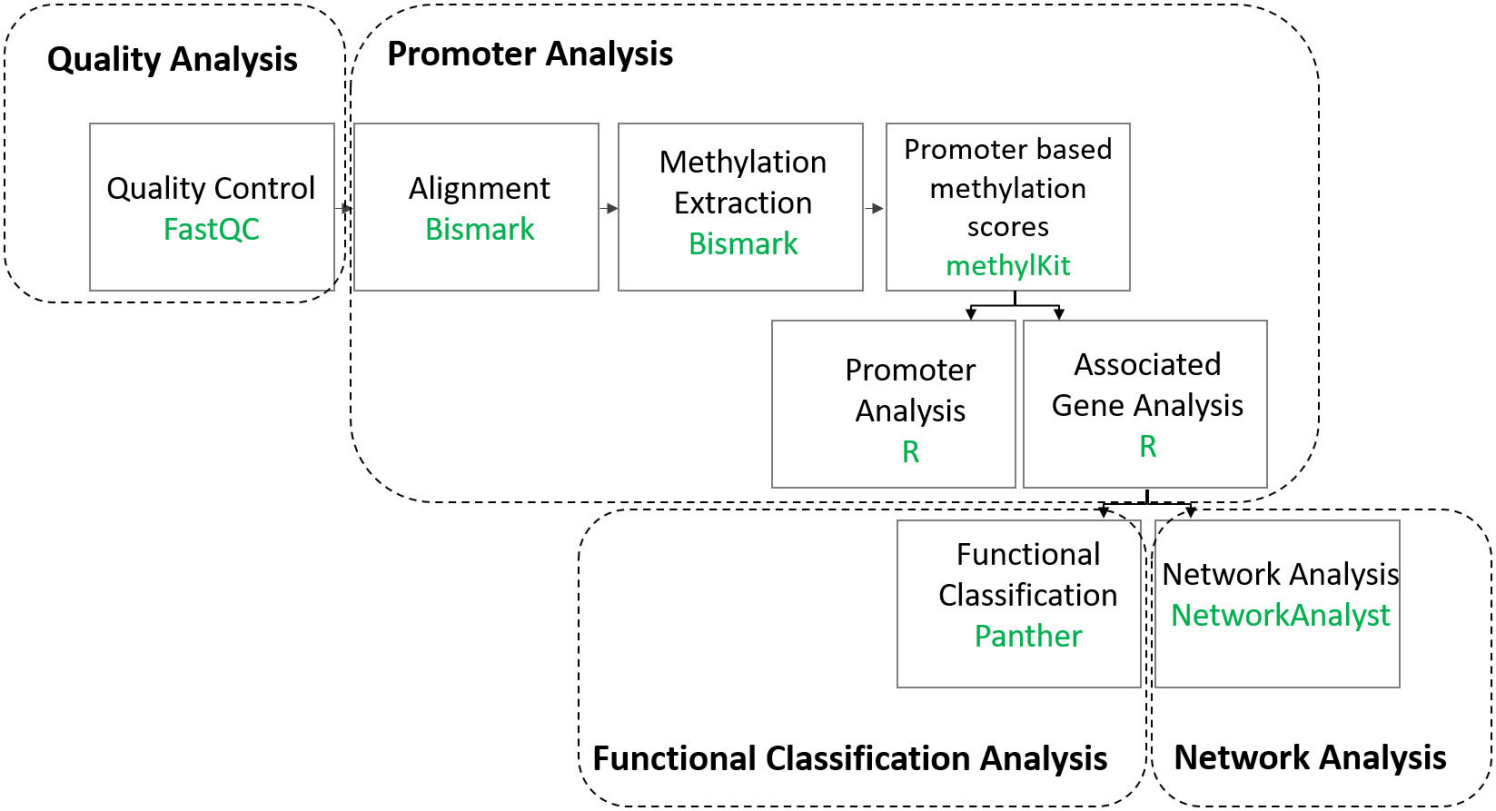
Summary of the data analysis workflow.

A summary of the bioinformatics analysis workflow can be found in (Figure 1, Supplementary Figure 2). First, raw reads were subjected to Quality Control (QC) using FastQC version 0.10.1 (15). Trim Galore version 0.3.7 was then used to remove low quality reads, adapters as well as RRBS-related residues that are artificially added during the end-repair step (16). Trimmed reads were then mapped to the UCSC Homo sapiens genome sequence (version hg19) using Bismark version 0.13.0 (17). To permit only up to one mismatch per seed region, the option “-n 1” was set for Bowtie version 1.0.0 utilized by Bismark (18). Methylation information was extracted from Bismark's sorted and filtered mapping results at base resolution using Bismark's methylation extraction software (17). The subsequent analysis was performed in the CpG context. The R package methylKit version 0.9.2 was used for further analysis of the Bismark methylation extraction reports (19). Samples that generated a minimum of 60 million reads (Supplementary Figure 1) were included in the analysis (Table 1) and processed methylKit. Methylation information found in the aforementioned extraction reports was summarized by methylKit over RefSeq promoters, defined as regions located 1 kb before or after a transcription start site (20). Coverage for each promoter was calculated as the sum of the methylated and unmethylated cytosines. The methylation percentage was calculated as a weighted average of cytosine methylation status (21), which corresponds to the overall proportion of methylated cytosines to the sum of all cytosines within the bin (here promoter with the specified coordinates), and which is covered by a minimum number of reads (21). The promoters were subsequently filtered based on coverage (minimal 5 and maximal 99.9 percentile) and merged for comparative analysis with only those promoters that are covered in all replicates being considered. Additional QC steps can be found in the Supplementary Materials Online (Supplementary Figures 3, 4). RRBS data was deposited in EMBL-EBI's European Genome-phenome Archive (EGA) and is accessible through EGA's accession number EGAD00001006303.

### Differentially Methylated Region Analysis

The difference in the methylation level of a promoter between aortic and mitral tissue was calculated as the difference between the weighted average of the percent methylation values of all mitral tissue samples and the weighted average of the percent methylation values of all aortic tissue samples at this promoter. A positive methylation difference (hypermethylation) indicated increased methylation in the promoter associated with mitral compared to aortic tissue. The significance of this difference was evaluated by methylKit using logistic regression (19). To correct for multiple hypothesis testing, the sliding linear model (SLIM) method was used by methylKit (19). DM promoters were finally filtered with the cut-off chosen as a methylation difference that is larger than 10 % and a q-value that is <0.05. Annotation of DMRs with genic features, primarily promoters, was carried out by methylKit using the genomation package (19).

### Enrichment and Functional Classification Analysis

The Protein Analysis Through Evolutionary Relationships (PANTHER) tool was utilized to perform functional enrichment analysis with the background of 16,101 promoters. PANTHER was also used to categorize genes whose promoters were found to be DM according to their molecular functions, biological processes, protein classes, pathways and cellular components (22).

### Construction of Protein-Protein Interaction Networks

Genes associated with DM promoters were used as seed genes to construct PPI networks using NetworkAnalyst (23). The International Molecular Exchange (IMEx) Interactome database, which contains literature-curated comprehensive data from InnateDB, was used by NetworkAnalyst for the generation of the generic network (23). For network construction, methylation difference values of alternative promoters of the same seed gene were replaced by their average before proceeding to network construction (23). The resulting network was trimmed to the minimum to encompass only nodes that connect the original seed genes. Functional enrichment analysis of all seed genes was performed using NetworkAnalyst's Function Explorer using GO, KEGG and Reactome databases. Enrichment *p*-values were computed based on the hypergeometric test utilized by the Function Explorer (23). NetworkAnalyst's Module Explorer, was used to identify smaller significantly densely connected subnetworks using the Walktrap algorithm (23). Visualization of PANTHER, KEGG and Reactome pathway terms was performed using the GOplot R package (24).

## Results

### Aortic and Mitral Valves Show Different Methylation Signatures in 584 Promoters

**Figure 2:**
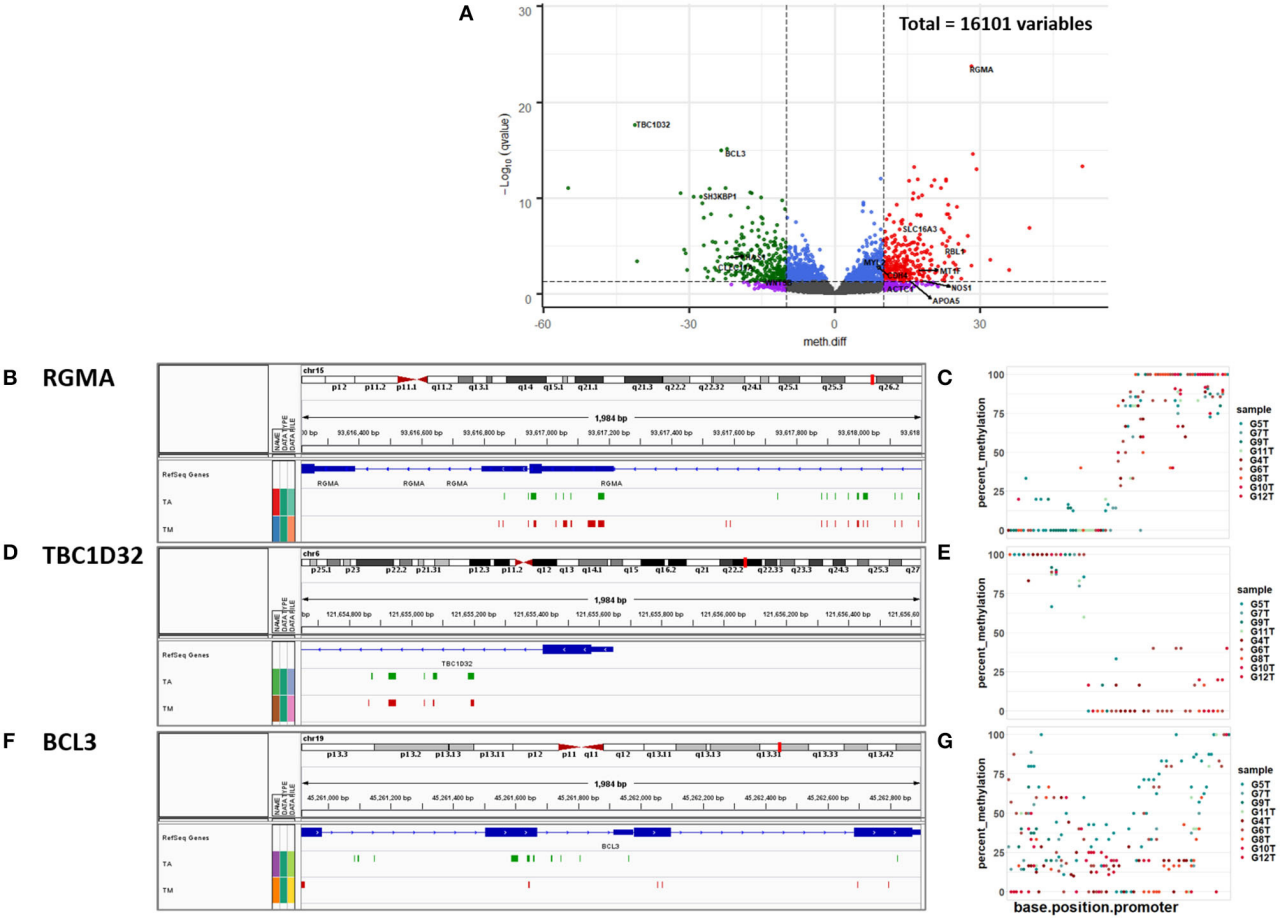
**(A)** Volcano plot of promoter methylation profiles. The vertical lines x = −10 and x = 10 represent our chosen methylation difference (meth.diff) cut-off value of |10|. meth.diff is calculated as described in the Methods. The horizontal line [y = -log_10_ (0.05) = 1.3] shows our chosen q-value cut-off of 0.05. The circles represent the 16,101 promoters, of which 584 are differentially methylated (DM). Each circle in the volcano plot represents a promoter with its meth.diff and -log_10_ (q-value). Gray circles denote promoters that are not DM. Purple circles represent promoters that are biologically, but not statistically significant. Blue circles depict the opposite trend. Red and green circles show promoters that are both statistically and biologically significant. Genes with a meth.diff > 0 (red) show increased methylation in the mitral compared to the aortic valve (↑) and genes with a meth.diff <0 (green) show decreased methylation in the mitral compared to the aortic valve (↓). Snapshots of the IGV genome browser showing the top 3 most significantly differentially methylated promoters linked to **(B)** RGMA **(C)** TBC1D32 and **(D)** BCL3, respectively (coordinates identified in Supplementary Table 1). TM denotes the union of the percent methylation values in all mitral samples that are >70% and which are present in the mitral but not in the aortic samples. TA denotes the union of the percent methylation values in all aortic samples that are >70% and which are present in the aortic but not in the mitral samples. Scatter plots of the percent methylation per base per promoter of the top 3 most significantly differentially methylated promoters linked to **(E)** RGMA **(F)** TBC1D32 and **(G)** BCL3, respectively (coordinates identified in Supplementary Table 1). Aortic samples: G5T, G7T, G9T, G11T; mitral samples (G4T, G6T, G8T, G10T, G12T).

DMR analysis identified 584 significantly DM promoters, of which 305 showed increased methylation in mitral and 279 in aortic valve tissue (Figure 2A, Supplementary Table 1). The gene Repulsive Guidance Molecule A (RGMA) was associated with the most significantly DM promoter (Figures 2B,C, Supplementary Table 1) followed by TBC1 Domain Family Member 32 (TBC1D32) (Figures 2D,E, Supplementary Table 1), B-Cell Lymphoma 3 (BCL3) (Figures 2F,G, Supplementary Table 1) and the long non-coding RNA RP11-1149O23.3 among others (Figure 2A, Supplementary Table 1).

**Figure 3:**
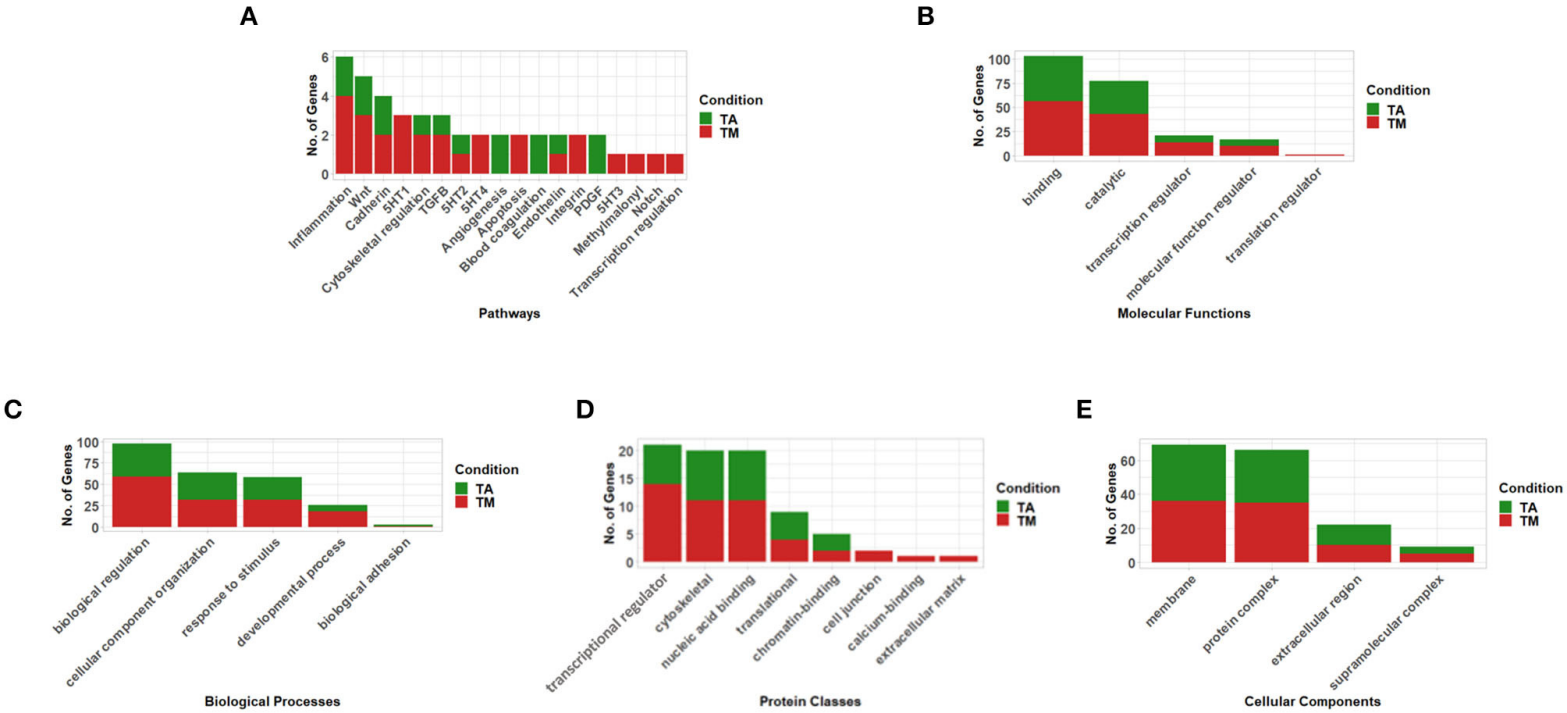
Functional classification of genes associated with differentially methylated promoters (q-value < 0.05 & meth.diff >|10|) between aortic and mitral tissue according to **(A)** Pathways, **(B)** Molecular Functions, **(C)** Biological Processes, **(D)** Protein Classes and **(E)** Cellular Components (selected results, the remaining can be found in Supplementary Figure 5– analysis performed using PANTHER). In the stacked bar graphs, TA (green) labels genes with promoters that show increased methylation in the aortic compared to the mitral valves and TM (red) shows genes whose promoters exhibit increased methylation in the mitral compared to the aortic valves.

When we investigated genes linked to the significant differentially methylated promoters in isolation, no significant enrichment was found. When functional classification analysis was performed, the genes associated with the DM promoters were grouped into key valve-related functions and pathways (Figures 3A–E, Supplementary Figure 5) with their methylation direction specified in (Supplementary Table 1). Key valve-related pathways such as Wingless/Integrated (WNT)-, Cadherin-, Transforming Growth Factor Beta (TGFB)-, Integrin-, Endothelin-, Platelet-Derived Growth Factor (PDGF)-, NOTCH signaling as well as angiogenesis and general transcription regulation were identified utilizing the PANTHER Pathway database (Figure 3A, Supplementary Table 2).

**Table 2:** List of pathways resulting from enrichment analysis of the network (Figure 4A) constructed upon the genes associated with the differentially methylated hypo- and hypermethylated promoters using the KEGG pathway database.

**KEGG Pathways**	**Tot^a^**	**Exp^b^**	**Hits^c^**	**Pval^d^**	**FDR^e^**	**Seed genes^f^**	**Other genes^g^**
Apoptosis	136	7.91	31	2.56E-11	6.8E-10	CASP7, TUBA8, CTSZ	DDIT3, BIRC2, TNFRSF1A, TRADD, IKBKG, RELA, NFKBIA, ACTB, XIAP, TP53, RAF1, CTSB, BIRC3, PIK3R1, FOS, NFKB1, FAS, AKT1, MAPK1, IKBKB, CTSD, TUBA1A, BCL2L11, TRAF2, ITPR3, JUN, FASLG, ACTG1
NF-κB signaling pathway	100	5.82	23	8.40E-09	1.03E-07	ZAP70, TAB1	TRAF6, UBE2I, BIRC2, TNFRSF1A, TRADD, IKBKG, NFKB2, RELA, NFKBIA, XIAP, BTK, IRAK1, MYD88, BIRC3, NFKB1, IKBKB, PLCG1, LCK, TRAF2, CSNK2B, LYN
Fluid shear stress and atherosclerosis	139	8.08	26	8.57E-08	8.01E-07	NQO1, GSTO2	SUMO3, TNFRSF1A, IKBKG, RELA, CAV1, ACTB, TP53, HSP90AA1, SQSTM1, PIK3R1, CTNNB1, FOS, NFKB1, AKT1, SRC, PLAT, IKBKB, RAC1, SUMO1, SUMO2, HSP90AB1, VEGFA, JUN, ACTG1
TNF signaling pathway	110	6.4	22	2.55E-07	2.19E-06	CASP7, TAB1, BCL3, DNM1L	BIRC2, TNFRSF1A, TRADD, IKBKG, RELA, NFKBIA, BIRC3, PIK3R1, FOS, NFKB1, FAS, AKT1, MAPK1, IKBKB, CREB1, TRAF2, CEBPB, JUN
Osteoclast differentiation	128	7.44	23	1.01E-06	7.32E-06	TAB1, LCP2, FHL2	TRAF6, TNFRSF1A, IKBKG, NFKB2, RELA, NFKBIA, BTK, SQSTM1, PIK3R1, FOS, NFKB1, AKT1, GRB2, MAPK1, IKBKB, RAC1, CREB1, LCK, TRAF2, JUN
IL-17 signaling pathway	93	5.41	19	1.20E-06	8.31E-06	CSF3	TRAF6, TRADD, IKBKG, RELA, NFKBIA, HSP90AA1, FOS, NFKB1, MAPK1, IKBKB, MAPK6, GSK3B, ELAVL1, S100A9, HSP90AB1, TRAF2, CEBPB, JUN
HIF-1 signaling pathway	100	5.82	19	3.78E-06	2.36E-05	LDHA	GAPDH, VHL, CREBBP, RELA, STAT3, ERBB2, EGFR, PIK3R1, NFKB1, HIF1A, AKT1, MAPK1, CDKN1A, EP300, CUL2, PLCG1, ENO3, VEGFA
Regulation of actin cytoskeleton	214	12.4	29	1.56E-05	8.69E-05	MYL2, PAK6, LIMK1, F2R, FGD1, FGD3	MYL12A, ACTB, WAS, PPP1CA, RAF1, CRK, EGFR, PIK3R1, SRC, MAPK1, IQGAP1, SOS1, RAC1, CDC42, FN1, WASL, CRKL, PPP1CC, PAK2, GIT1, VAV2, ACTG1, ARHGEF7
VEGF signaling pathway	59	3.43	10	0.00192	0.00671	NA	RAF1, PIK3R1, AKT1, SRC, MAPK1, RAC1, CDC42, PLCG1, PLA2G4A, VEGFA
TGFB signaling pathway	92	5.35	13	0.00241	0.00817	RGMA, RBL1, SMAD3, E2F4, HAMP	CREBBP, MYC, SMAD2, SMAD7, MAPK1, SP1, EP300, CUL1

a *“Tot” refers to the total number of genes that belong to the particular KEGG pathway as per the chosen reference list of genes, which is hg19 in our case*,

b *“Exp” denotes the number of genes to be expected in our gene list for the particular KEGG pathway*,

c *“Hits” describes the number of genes in our list that map to the particular KEGG pathway*,

d *“Pval” is equivalent to the enrichment p-value computed using the hypergeometric test (see Methods)*,

e *“FDR” stands for false discovery rate, which is the method used to correct the corresponding p-value for multiple testing*,

f *“Seed genes” represent genes associated with significantly differentially hyper- and hypomethylated promoters and*

g *“Other genes” denote genes that are part of the minimum non-seed genes that are necessarily required to connect the seed genes to construct the network. The methylation direction of the seed genes can be found in Supplementary Table 1*.

When the genes were classified by their molecular function, 69.5% of the genes had a binding and catalytic activity including key genes such as Nitric Oxide Synthase 1 (NOS1) (q-value = 0.04, meth.diff = 17.9 %). Other genes exhibited transcription regulator-, translation regulator- and molecular function regulator activities. The latter category included relevant genes such as Apolipoprotein A5 (APOA5) (q-value = 0.037, meth.diff = 15.3%) and Natriuretic Peptide B (NPPB) (q-value = 0.0027, meth.diff = 15.58%) (Figure 3B, Supplementary Table 3).

Among the relevant biological processes categorizing the genes were “biological regulation” and “cellular component organization or biogenesis.” The former included notable genes such as Metallothionein 1F (MT1F) (q-value = 0.004, meth.diff = 17.07%) and the latter included two pertinent ones namely Hyaluronan Synthase 1 (HAS1) (*q*-value = 0.00015, meth.diff = 22.15%) as well as Actin Alpha Cardiac Muscle 1 (ACTC1) (q-value= 0.049, meth.diff = 11.23%) (Figure 3C, Supplementary Table 4).

Genes were also grouped into relevant protein classes such as gene-specific transcriptional regulator proteins, which included SMAD Family Member 3 (SMAD3) (*q*-value = 0.0019, meth.diff = 10.25%), cytoskeletal proteins, which contained Myosin Light Chain 2 (MYL2) (q-value = 0.013, meth.diff = 11.14%) and chromatin-binding protein, which encompassed RB Transcriptional Corepressor Like 1 (RBL1) (q-value = 9.5e-06, meth.diff = 22.98%) (Figure 3D, Supplementary Table 5).

Finally, the cellular component categories into which the genes could be classified were membrane- and extracellular regions among others. Relevant genes such as C-Type Lectin Domain Containing 11A (CLEC11A) (q-value = 0.002, meth.diff = 24.72%) were included in the latter and Solute Carrier Family 16 Member 3 (SLC16A3) (q-value = 1.78e-07, meth.diff = 13.4%) in the former category (Figure 3E, Supplementary Table 6).

### Network Analysis Enabled a Systems-Based Assessment and Revealed Additional Valve-Related Pathways Such as Hypoxia-Inducible Factor 1 and Vascular Endothelial Growth Factor Signaling

After functionally categorizing the genes, whose promoters were significantly DM between the aortic and mitral valves on a genome-wide level, we wanted to assess whether we can predict significantly enriched PPIs among those genes as well as the proteins that are necessary to interconnect them to enable a systems-level analysis.

**Figure 4:**
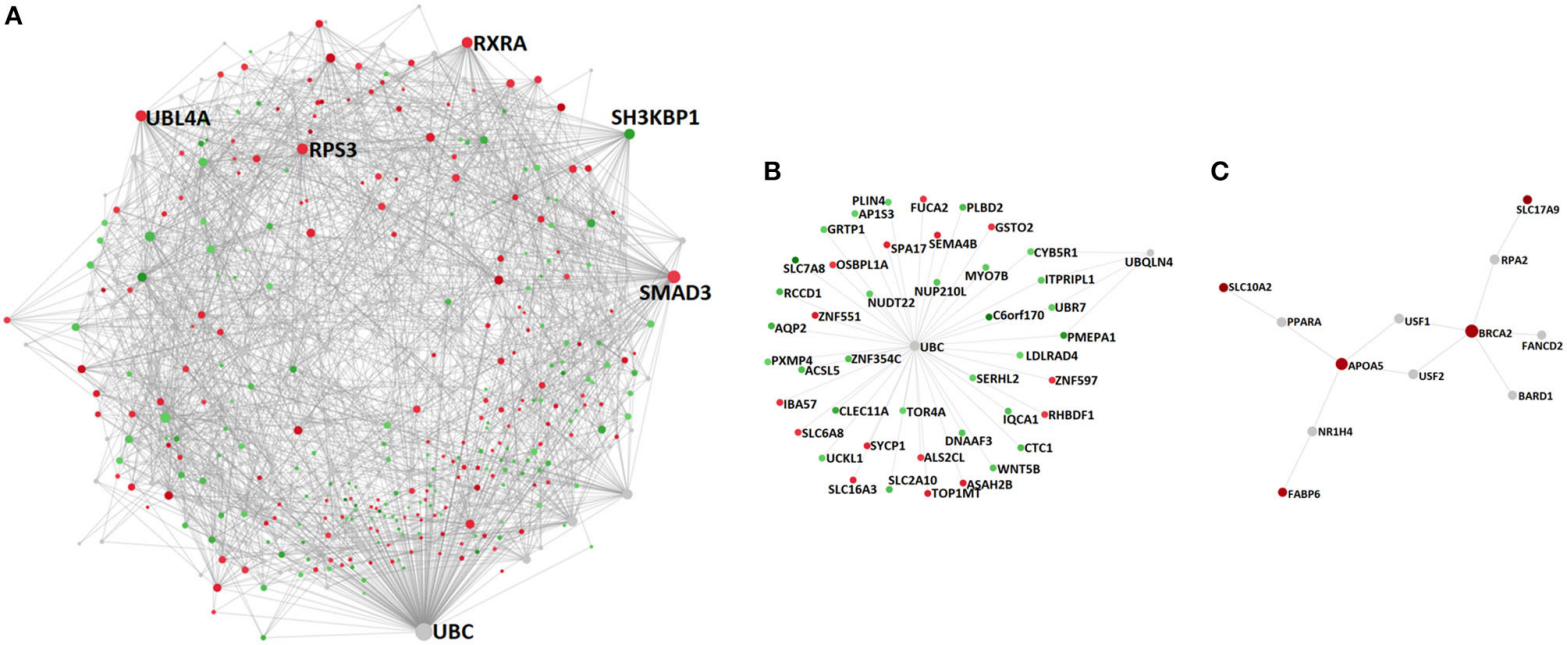
PPI networks constructed upon the genes associated with differentially methylated promoters between non-diseased aortic and mitral valve tissue and the proteins that are necessary to interconnect them: **(A)** complete network, **(B)** subnetwork 1 (*p*-value 3.04e-13) and **(C)** subnetwork 2 (*p*-value 0.047). Depicted nodes are classified according to their methylation direction: nodes representing genes showing increased methylation in mitral compared to aortic tissue (red), nodes depicting increased methylation in aortic vs. mitral tissue (green) and nodes representing genes that are not part of the input dataset (gray). Node sizes are proportional to their betweenness centrality values. Betweenness centrality reflects the number of shortest paths passing through a node, while degree refers to the number of connections/edges/PPIs that a node has to other nodes. Hub nodes **(A)** UBC, SMAD3, UBL4A, RPS3, RXRA, **(B)** UBC and **(C)** BRCA2 and APOA5 can be clearly identified on the respective networks.

For that purpose, we mapped those DM genes onto a generic PPI database (see Methods) and constructed a PPI network, which ultimately comprised 715 nodes (genes) of which 308 were seed genes and 2,131 edges (PPIs) (Figure 4A, Supplementary Table 7). Among the network's constituent proteins were several hub nodes, the most connected of which were Ubiquitin C (UBC) (Betweenness centrality = 129,377.98; Degree = 200), SMAD3 (Betweenness centrality = 27,152.04; Degree = 75), Ubiquitin Like 4A (UBL4A) (Betweenness centrality = 13,019.92; Degree = 50), Ribosomal Protein S3 (RPS3) (Betweenness centrality = 9,508.1; Degree = 50), Retinoid X Receptor Alpha (RXRA) (Betweenness centrality = 12,661.72; Degree = 47) and SH3 Domain Containing Kinase Binding Protein 1 (SH3KBP1) (Betweenness centrality = 13,005.65; Degree = 44) (Figure 4A, Supplementary Table 7).

In addition to inspecting the topological properties of the constructed network, we performed functional enrichment analysis of both the hyper- and hypomethylated proteins of the subnetwork as well as them connecting these proteins. This analysis showed significant enrichment of pathways and functions.

Many pathways, which are pertinent to valvular mechanisms, were identified *via* KEGG pathway enrichment analysis including apoptosis, Nuclear Factor Kappa-light-chain-enhancer of activated B cells (NF-κB) signaling pathway, fluid shear stress and atherosclerosis, Tumor necrosis factor (TNF) signaling pathway, osteoclast differentiation, Interleukin 17 (IL-17) signaling pathway, HIF-1 signaling pathway, regulation of actin cytoskeleton, VEGF signaling pathway and TGFB signaling pathway (FDR < 0.05, Table 2).

To investigate whether relevant significantly enriched pathways are present independent of the choice of the pathway database, we furthermore performed Reactome enrichment analysis, which exclusively highlighted additional key valve-related pathways such as immune response, regulation of lipid metabolism, PDGF, NOTCH1, Fibroblast Growth Factor Receptor (FGFR) and transcription (FDR < 0.05, Supplementary Table 8). Similar to KEGG, it showed enrichment for apoptosis-, TFGB-, interleukin- and hypoxia- related pathways (Table 2, Supplementary Table 8).

After inspecting significant valve-related pathways, we checked whether there are enriched biological processes, molecular functions and cellular components related to the molecular regulation of valve-related processes based on GO databases. Relevant biological processes included transcription, apoptosis, growth factor signaling as well as regulation of cellular component organization (FDR < 0.05, Supplementary Table 9). Molecular functions associated with transcription as well as histone acyltransferase activity such as chromatin-, histone deacetylase-, TF-, SMAD- and NF-κB-binding were among the pertinent significantly enriched molecular functions (FDR < 0.05, Supplementary Table 10). Finally, the most relevant significantly enriched cellular components were Transcription Factor II D (TFIID) complex, spliceosomal complex, chromatin, histone deacetylase complex as well as actin cytoskeleton (FDR < 0.05, Supplementary Table 11).

In addition to examining the network structure as a whole, we performed a module analysis (see Methods), to identify subnetworks that show a significantly increased connection density compared to other modules of the parent network. The first identified subnetwork (*p*-value 3.04e-13) comprised 45 nodes, of which 43 were seed genes and contained the following hub nodes: UBC, also identified as a hub protein of the parent network (Betweenness centrality = 43; Degree = 939) and Ubiquilin 4 (UBQLN4), not identified previously (Betweenness centrality = 3; Degree = 4) (Figure 4B, Supplementary Tables 12, 13).

The second subnetwork (*p*-value 0.047) contained 12 nodes, of which 5 were seed genes with Breast And Ovarian Cancer Susceptibility Protein 2 (BRCA2) (Betweenness centrality = 33.5; Degree = 5) as well as APOA5 (Betweenness centrality = 32.5; Degree = 4) being its main hub proteins. Interestingly, all of the module's constituent seed nodes exhibited increased methylation in mitral compared to aortic tissue (Figure 4C, Supplementary Table 14). Relevant enriched pathways in this module included homologous recombination (KEGG/Reactome) and Peroxisome Proliferator-Activated Receptor (PPAR) signaling pathway (KEGG) (FDR < 0.05, Supplementary Table 15). The module's biological processes contained lipid homeostasis and cellular response to external stimulus (FDR < 0.05, Supplementary Table 15). Finally, the most relevant enriched molecular functions encompassed DNA-, enzyme- and TF- binding (FDR < 0.05, Supplementary Table 15).

### Global Analysis of Detected Pathways and Genes Shows the Role of Methylation in EMT and ECM Remodeling

All the utilized analysis methods of genes and pathways show that DNA methylation plays a crucial role in valve development and disease. Indeed, some of the detected genes and pathways are involved in developmental processes such as cardiogenesis, EMT and protein QC and/or diseases such as myxomatous mitral valve (MMV), BAV and AD (Tables 3, 4).

**Table 3:** Genes associated with differentially methylated promoters between aortic and mitral valve tissue and their involvement in valve development and disease.

**Gene**	**Description**	**Development**	**Disease**
NOS1	Nitric oxide synthase 1 (neuronal)	Heart (25)	BAV^a^ (25)
ACTC1	Actin, alpha, cardiac muscle 1	Heart (26)	MMV^b^ (27)
MYL2	Myosin, light chain 2, regulatory, cardiac	Heart (28)	
MT1F	Metallothionein 1F		MMV (29)
CLEC11A	C-Type lectin domain containing 11A		MMV (30)
RBL1	Retinoblastoma-like 1 (p107)		BAV (31), AS (32)
SLC16A3	Solute carrier family 16, member 3 (monocarboxylate transporter)		BAV (33), AS^c^ (34)
NPPB	Natriuretic peptide B	EMT^d^ (35)	MR^e^ (36)
CDH4	Cadherin 4, type 1, R-cadherin (retinal)	Valve (37)	
HAS1	Hyaluronan synthase 1		CAVD^f^ (38)
WNT5B	Wingless-type MMTV integration site family, member 5B		CAVD (39)
Ubiquitin-related genes		Protein QC^g^ in the heart (40)	BAVs (41), atherosclerosis (42)
SMAD3	SMAD family member 3	Cardio-genesis (43)	AD^h^ (44)
RXRA	Retinoid X receptor, alpha		Valve malformation (45)
SH3KBP1	SH3-domain kinase binding protein 1		CAVD (46)
APOA5	Apolipoprotein A-V		AS (47–49)

a *BAV, bicuspid aortic valve*;

b *MMV, myxomatous mitral valve*;

c *AS, aortic valve stenosis*;

d *EMT, endothelial mesenchymal trans-differentiation*;

e *MR, mitral valve regurgitation*;

f *CAVD, calcific aortic valve disease*;

g *QC, quality control*;

h *AD, thoracic aortic aneurysm and dissection*.

**Table 4:** Pathways involving the genes associated with the differentially methylated promoters between aortic and mitral valve tissue and their implication in valve development and disease.

**Pathway**	**Description**	**Development**	**Disease**
TGFB signaling	Transforming growth factor beta signaling	EMT^a^ (50)	ECM^b^ remodeling (51), MVP^c^ (52), AS^d^ (53, 54)
NOTCH signaling		EMT (50), (55)	BAV^e^ (55, 56), AS (55) and fetal cardiac defects (57)
FGF signaling	Fibroblast growth factor signaling	EMT (58)	Valve malformation (58)
WNT signaling		heart, EMT (39, 50)	
Cadherin signaling		EMT (59)	
PDGF signaling	Platelet-derived growth factor	heart (60)	
VEGF signaling		EMT (50)	
Integrin signaling		EMT (50), cell-ECM (61, 62)	
HIF-1 signaling			RMV^f^, MMV^g^ (63)
Angiogenesis			RMV (64)
IL-17 signaling			IE^h^ (65)
NF-κB signaling			AS (66)
TNF signaling			AS (67)
Osteoclast differentiation pathways			AS (68)
Endothelin signaling		VICs^i^ regulation by VECs^j^ (69)	AS (70)
Apoptotic pathways			AS (71)
PPAR signaling		lipid metabolism (72)	

a *EMT, endothelial mesenchymal trans-differentiation*;

b *ECM, extracellular matrix*;

c *MVP, mitral valve prolapse*;

d *AS, aortic valve stenosis*;

e *BAV, bicuspid aortic valve*;

f *RMV, rheumatic mitral valve*;

g *MMV, myxomatous mitral valve*;

h *IE, infective endocarditis*;

i *VICs, valve interstitial cells*;

j *VECs, valve endothelial cells*.

**Figure 5:**
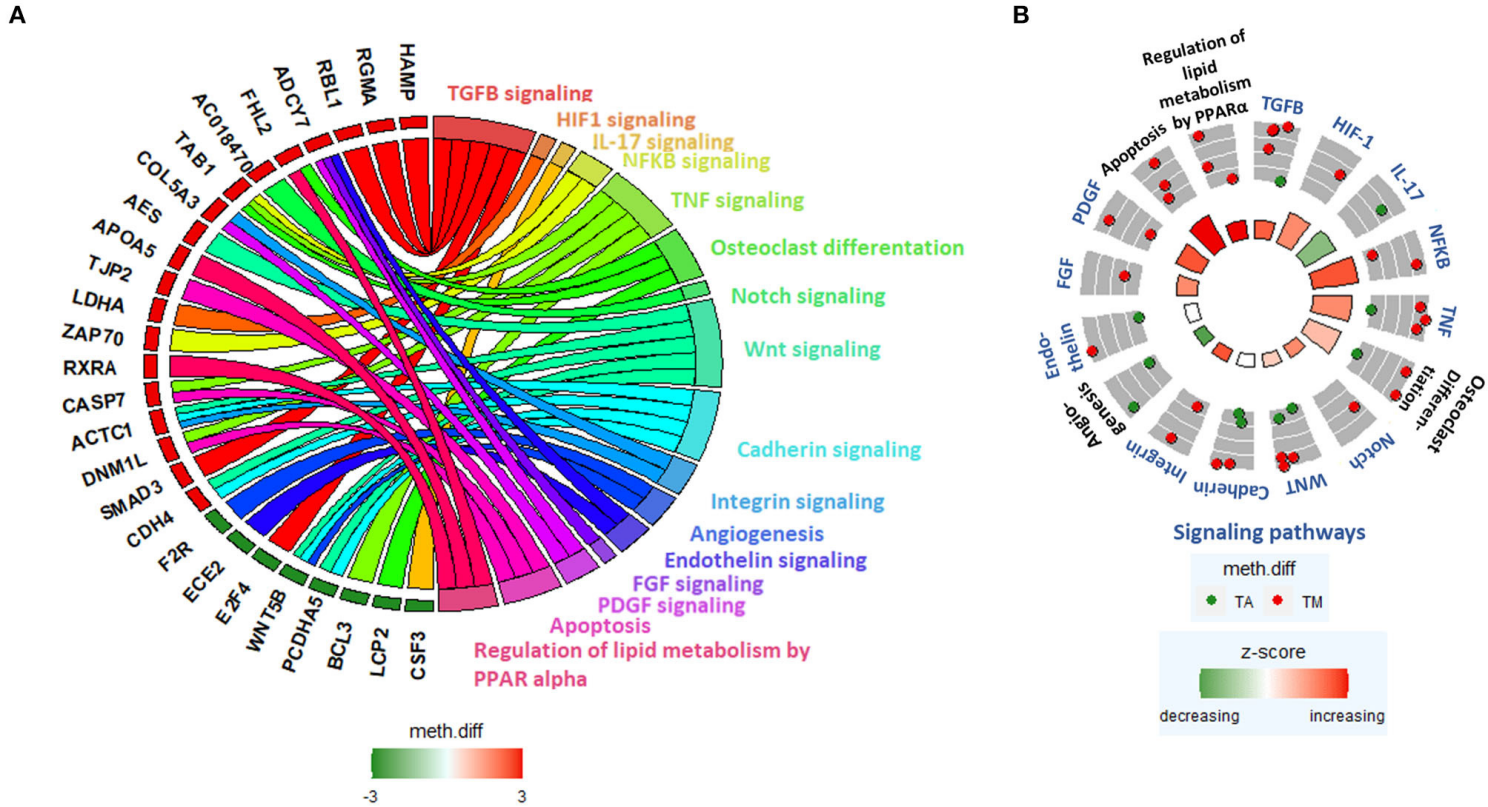
**(A)** GOChord plot linking selected pathways to their constituent genes, which are associated with DM promoters. Green-to-red colors next to the selected genes reflect their meth.diff values. **(B)** GOCircle plot representing the relevant pathways and their constituent genes that are associated with DM promoters. The inner circle consists of bar plots, whose heights reflect the significance of the pathway and whose color reflect the z-score, which approximates the overall direction of change in methylation for each pathway. The outer circle shows scatterplots of the meth.diff values of each of the pathway's constituent genes.

We further generated a GOChord plot to link the pathways in Table 4 to their constituent genes, which are associated with DM promoters (Figure 5A). The plot revealed several pathways sharing the same genes, such as NF-κB signaling, TNF signaling and osteoclast differentiation, which contain TGFB Activated Kinase 1 Binding Protein 1 (TAB1) and Integrin- and PDGF signaling, which include Collagen Type V Alpha 3 (COL5A3) (Figure 5A).

A GOCircle plot visualizes the significance of each pathway in Table 4 and to show the direction of promoter methylation of their constituent genes (Figure 5B). The most significant pathways in Table 4 were NF-κB, TNF signaling and osteoclast differentiation, which share TAB1 (Figure 5A) and are implicated in aortic stenosis (AS) (Table 4).

## Discussion

This study provides new information regarding the DNA methylation landscape in human non-diseased heart valves. Heart valves have a complex structure and function that are sensitive to their environment and exhibit characteristic phenotypic and functional differences. Valve-type specific differences begin to appear during valve formation and development and are expressed in the valve's distinct anatomical structures, environmental milieus and susceptibility to disease. In this work we focus on two of the four valves, the mitral and aortic valves and their epigenetic profiles.

While there are several studies exploring the epigenetics of abnormal valves in the literature (57, 73, 74), they are not standardized and do not include methylation of normal valves. Previous studies have addressed how DNA methylation affects valvular disease processes. These studies investigated DNA methylation mechanisms that can transform VICs of stenotic aortic valves into leukotriene-producing immune-like cells *via* targeted promoter methylation measurement of 5-lipoxygenase (5-LO) (75), and those that lead to the disruption of both the organization of the ECM and the communication between the cells and the ECM in bicuspid aortic valve (BAV) by investigating miR-29 expression level utilizing qRT-PCR (76). Other studies focused on uncovering associations between methylation changes and the development of rheumatic heart valve disease using ELISA (77), and on detecting the genome-wide DNA methylation landscape underpinning BAV and aortic dissection (AD) *via* methylation array (78).

We used RRBS to measure DNA methylation as it targets CpG-rich islands and promoters genome-wide (79). It is crucial to couple this sequencing method with suited bioinformatics workflows, that rely on rigorous QC of RRBS-characteristic issues as well as of bisulfite sequencing-specific parameters such as the efficiency of bisulfite conversion (80). RRBS thus can allow for a comprehensive view not limited by predefined sets of CpG loci probes (81).

In this study, 584 of 16,101 promoters were found to be DM between aortic and mitral tissue and their associated genes associated were found to be implicated in valvular health and disease mechanisms. RGMA was associated with the most significantly DM promoter. It is a member the RGM protein family, the first known BMP selective co-receptor family able to induce BMP signaling that is dysregulated in CAVD (82, 83). TBC1D32, the gene associated with the second most DM promoter, is implicated in the pathogenesis of ciliopathies in humans (84), which are caused by defects in the human primary cilium known to play a role in establishing left-right asymmetry during heart development (85), to restrain ECM production during physiological aortic valve development and to play a role in the etiology of BAV in humans (86). Finally, BCL3, the gene associated with the third most DM promoter, is known to play a role in atherosclerosis (87), with atherosclerosis-like lesions potentially leading to AS (88).

Further genes, that were associated with DM promoters (summarized in Table 3) include NOS1, ACTC1 and MYL2, which play key roles in heart development (25, 26, 28), with ACTC1 additionally being implicated in MMV and NOS1 in BAV (25, 27). MT1F and CLEC11 further contribute to MMV (29, 30), while RBL1 and SLC16A3 to BAV (31, 33). NPPB is involved in EMT by exhibiting excessive synthesis of the cardiac jelly, a precursor of the cushions, in zebrafish and is overexpressed in the ventricles of patients with chronic volume overload caused by regurgitant mitral valve lesions (35, 36). Cadherin-4 (CDH4) is both significantly DM and expressed during the development of embryonic mice (37). Finally, HAS1 and Wingless/Integrated 5B (WNT5B) contribute to CAVD (38, 39), and SLC16A3 to AS (34).

The network constructed upon the genes linked to DM promoters identified hub proteins known to be involved in different aortic and mitral valve mechanisms. UBC and UBL4A, the two most connected hub genes of the network, belong to the ubiquitin family, whose members play a role in BAVs as well as in atherosclerosis (41, 42). The network's first submodule was additionally entirely centered around UBC further highlighting the importance of the ubiquitin system, which is key to performing protein QC in the heart (40). SMAD3, the second most connected hub node, plays an important role in cardiogenesis (43), and is linked to thoracic aortic aneurysm and dissection (44). RXRA, a member of the RA signaling pathway and the network's third hub node, is linked to OFT and AV canal malformations, which influence proper aortic and mitral valve development (45). Finally, the hub node SH3KBP1 is implicated in CAVD (46). The second submodule of the network contained only two hub nodes APOA5 and BRCA2. Dyslipidemia linked to Lipoprotein a (LPA)-associated APOA5 has been detected in AS (74), with the reduction of LPA levels *via* PSCK9 inhibitors constituting promising therapeutic avenues for AS treatment (47–49). BRCA2, has not been associated with valvular mechanisms in the literature and thus should be further investigated.

Detected pathways were relevant to valvular development and disease (Table 4). For example, TGFB- and NOTCH signaling pathways activate EMT by downregulating VE-Cadherin (50, 55), which decreases cell adhesion of the transforming endocardial cells enabling them to break away from the endocardium and to migrate into the cardiac jelly, where they can transform into mesenchyme cells creating cushions that expand and fuse to ultimately form cardiac valves (50). TGFB signaling is also implicated in pathological ECM remodeling (51), MVP (52), AS (53), and NOTCH pathways in BAV and AS (55). FGF signaling further promotes OFT myocardial cell invasion to the cardiac cushion during EMT, with its disruption leading to malformed OFT valves in mice (58). Canonical WNT- and Cadherin signaling add to cushion development and remodeling during EMT (50, 59), with WNT pathways being additionally implicated in valve stratification and well as in the patterning of the heart forming field (50). Similar to WNT- and Cadherin-, the PDGF signaling pathway is also involved in cardiogenesis, particularly in the formation of the primordial heart tube (60). Both the VEGF- and Integrin signaling pathways contribute to post-EMT maturation, in that the former establishes an equilibrium between proliferation and differentiation of cells in the cushion (50), and the latter enables ECM remodeling through the generation of a mechano-transducing network that connects the cells to the ECM providing a link that relays external metabolic and hemodynamic factors (61, 62). HIF-1 signaling is involved in pathological ECM remodeling associated with RMV and MMV disease (63). Aberrant angiogenesis and IL-17 signaling are also implicated in RMV and infective endocarditis (IE), respectively (64, 65). NF-κB-, TNF-, Osteoclast differentiation-, Endothelin and Apoptotic pathways are involved in AS (66–68, 70, 71), with the Endothelin pathway additionally being implicated in the regulation of VICs by VECs (69). Finally, the detected PPAR signaling pathway is linked to lipid metabolism and is enriched among other lipid-related genes in the second mitral valve-specific subnetwork (72). The enrichment of PPAR signaling, the uniform increased methylation of the subnetwork's constituent genes in mitral compared to aortic valves and APOA5 being a hub node, indicate that this subnetwork exhibits major methylation alterations related to the metabolism of lipids. The regulation and expression of lipid-related genes need to be further dissected as it has been reported in a previous study that increased fatty infiltration of valves is observed in MVP (89).

One very interesting aspect of this dataset is having 4 matched pairs (Table 1). Comparisons within individuals that have both aortic and mitral samples (Supplementary Figures 6A–F) revealed that promoters identified by our original non-matched analysis have been re-captured in the matched analysis with additional promoters identified by the matched analysis (Supplementary Figures 6A–F). Matched analysis can provide additional insight particularly when powered by sufficient replicates as it eliminates inherent genetic differences between samples which needs to be integrated in the design of the future large-scale study.

Our analysis provided a comprehensive catalog of genes and pathways that are differentially regulated between aortic and mitral valves, establishing the basis for upcoming whole-genome bisulfite sequencing (WGBS) studies to additionally interrogate methylation of gene-bodies and other non-promoter regions. Additional insights can be obtained from histone-modification and RNA-based experiments as well as the interaction of such epigenetic mechanisms with DNA methylation. Functional validation of genes and pathways of interest on the transcriptomic and proteomic level will confirm candidate DM biomarkers, which can serve as potential drug targets. Additionally, the same analysis on the VIC level will be done to confirm cell-type specific signals that might have been affected by the tissue's intrinsic cell heterogeneity. Such analysis will provide novel mechanistic insights into the distinct roles of the individual components.

Limitations-In this study, due to the scarcity of human non-diseased donor heart valves, a relatively small number of valves was examined (*n* = 12) and two of the four heart valves were studied. Further validation is required to evaluate the clinical significance of the methylation markers identified. An enhancement of the methodology used in this manuscript will be utilized in future studies for example by performing WGBS.

## Conclusion

To conclude, this is the first study that explores the genome-wide DNA methylation landscape characterizing human non-diseased aortic and mitral valves. By investigating genes that are linked to DM promoters and their associated pathways, we discovered that the cells as well as the ECM of the aortic and mitral valve have different methylation signatures. The detected pathways included TGFB-, NOTCH-, FGF-, WNT-, Cadherin- and VEGF signaling pathways associated with EMT, Integrin- and HIF-1 signaling linked to ECM remodeling and NF-κB-, TNF-, osteoclast differentiation, Endothelin- and IL-17 signaling observed in aortic and mitral valve disease. Especially with the increasing incidence and prevalence of valve disease worldwide due to the world's increasing population age in developed and the failure to address RHVD in low and middle-income countries (LMICs) (5), it is very important to acquire a better understanding of the genetic and epigenetic make-up of cardiac valves and how they are influenced by local conditions, environmental factors and ethnicities as this will affect the development of preventative and therapeutic strategies.

## Data Availability Statement

The datasets presented in this study can be found in online repositories. The names of the repository/repositories and accession number(s) can be found below: https://ega-archive.org/datasets/EGAD00001006303, EGAD00001006303; https://ega-archive.org/studies/EGAS00001004559, EGAS00001004559.

## Ethics Statement

The studies involving human participants were reviewed and approved by Royal Brompton Hospital Ethics Review Board / Brompton and Harefield trust Ethics Committee (REC approval 10/H0724/18). The patients/participants provided their written informed consent to participate in this study.

## Author Contributions

NL, YA, and MY: conceptualization. SH: data curation, formal analysis, validation, writing–original draft, and software. YA and MY: funding acquisition. SH, NL, Y-TT, and AHC: investigation. SH, NL, AM, YA, and MY: methodology. NL and YA: project administration. NL, AMI, AHC, and YA: resources. AM, YA, and MY: supervision. SH, AM, and YA: visualization. NL, AM, YA, and MY: writing–review and editing. All authors contributed to the article and approved the submitted version.

## Funding

This research was funded by Magdi Yacoub Institute (MYI) and Magdi Yacoub Foundation (MYF). SH was partially supported by Al Alfi Foundation (Al Alfi PhD Fellowship in Applied Sciences and Engineering).

## Conflict of Interest

The authors declare that the research was conducted in the absence of any commercial or financial relationships that could be construed as a potential conflict of interest.

## Publisher's Note

All claims expressed in this article are solely those of the authors and do not necessarily represent those of their affiliated organizations, or those of the publisher, the editors and the reviewers. Any product that may be evaluated in this article, or claim that may be made by its manufacturer, is not guaranteed or endorsed by the publisher.
