# Supplementary material for: Profiling Genome-Wide DNA Methylation Patterns in Human Aortic and Mitral Valves
Source: Front Cardiovasc Med. 2022 Apr 6;9:840647. doi: 10.3389/fcvm.2022.840647 (PMC9019152; doi:10.3389/fcvm.2022.840647)
Supplement: Supplementary file 1 [file Data_Sheet_1.pdf]

## 1 Supplementary Figures

### 1.1 Figure S1. Total no. of sequences for all samples before and after trimming

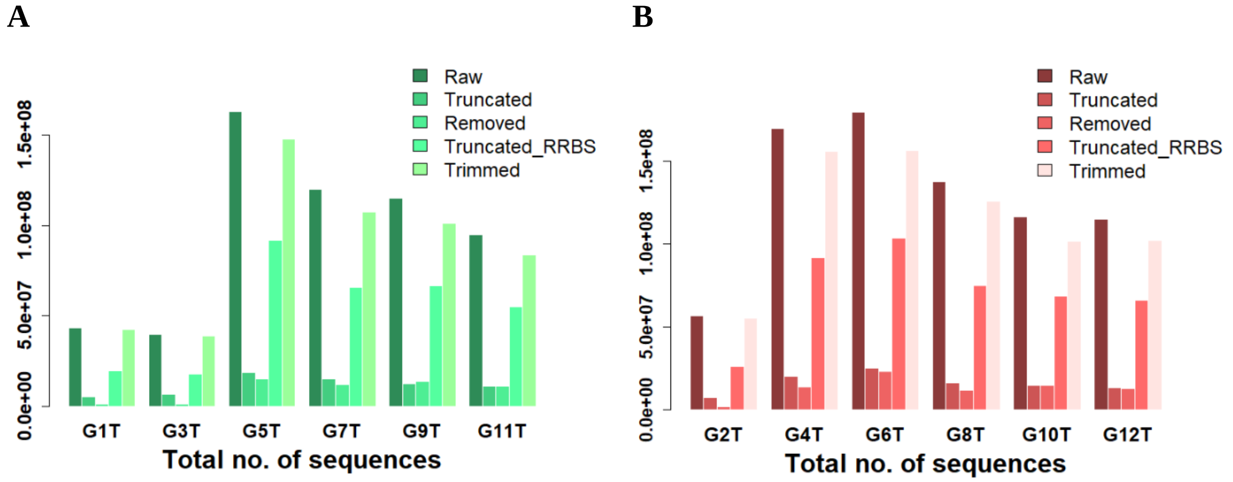


**Supplementary Figure 1.** Total no. of sequences for (**A**) aortic tissue (TA) samples G1T, G3T, G5T, G7T, G9T and G11T and (**B**) mitral tissue (TM) samples G2T, G4T, G6T, G8T, G10T and G12T. "Raw" refers to the total no. of sequences processed before trimming; "Truncated" denotes sequences that were truncated to a varying degree because of deteriorating qualities (Phred score < 20); "Removed" represents sequences that were removed as they became shorter than 20 bps after trimming; "Truncated_RRBS" represents sequences trimmed by an additional 2 bp characteristic of RRBS when adapter contamination was detected; "Trimmed" refers to the total no. of sequences processed after trimming. The total number of sequences processed for samples G1T, G3T and G2T before and after trimming were significantly less compared to the rest of the samples. The threshold set to remove technical artifacts (**see Methods**), automatically removed these samples from our analysis ensuring that no technical bias is introduced.

## 1.2 Figure S2. Detailed data analysis workflow.

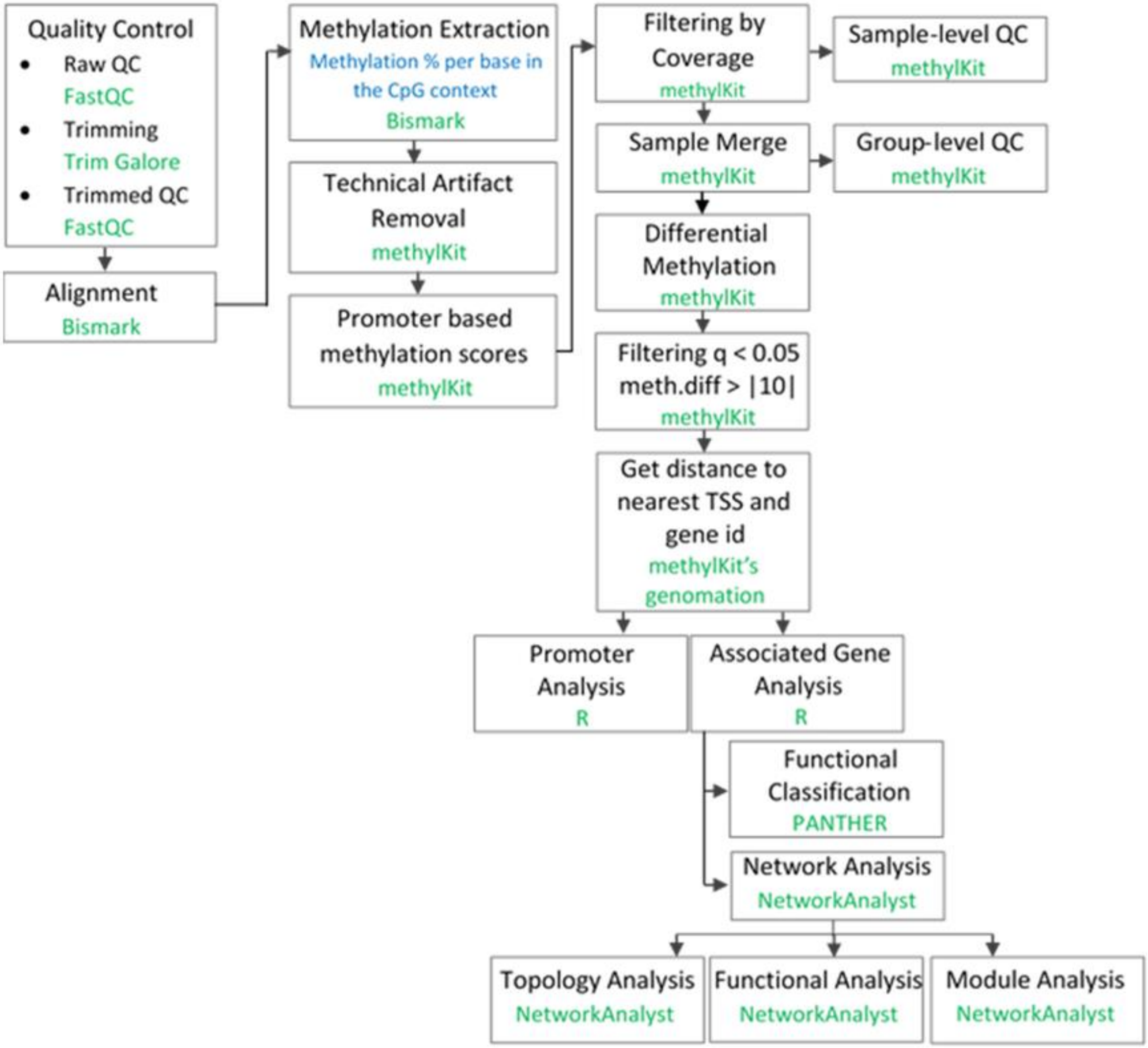


**Supplementary Figure 2.** Analyses are indicated in black, software utilized in green and software specifications in blue.

### 1.3 Figure S3. Methylation percentage and read coverage distribution per promoter for aortic and mitral tissue samples

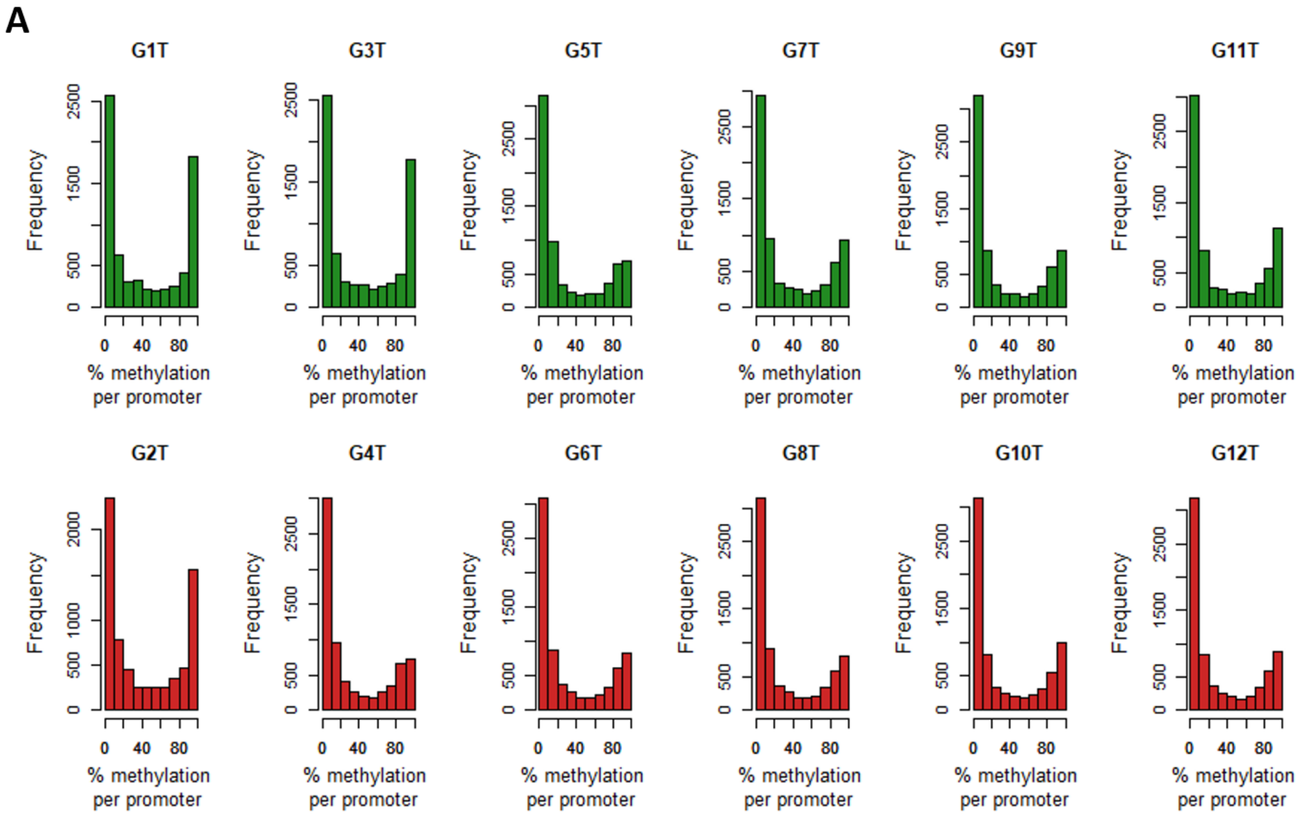

B

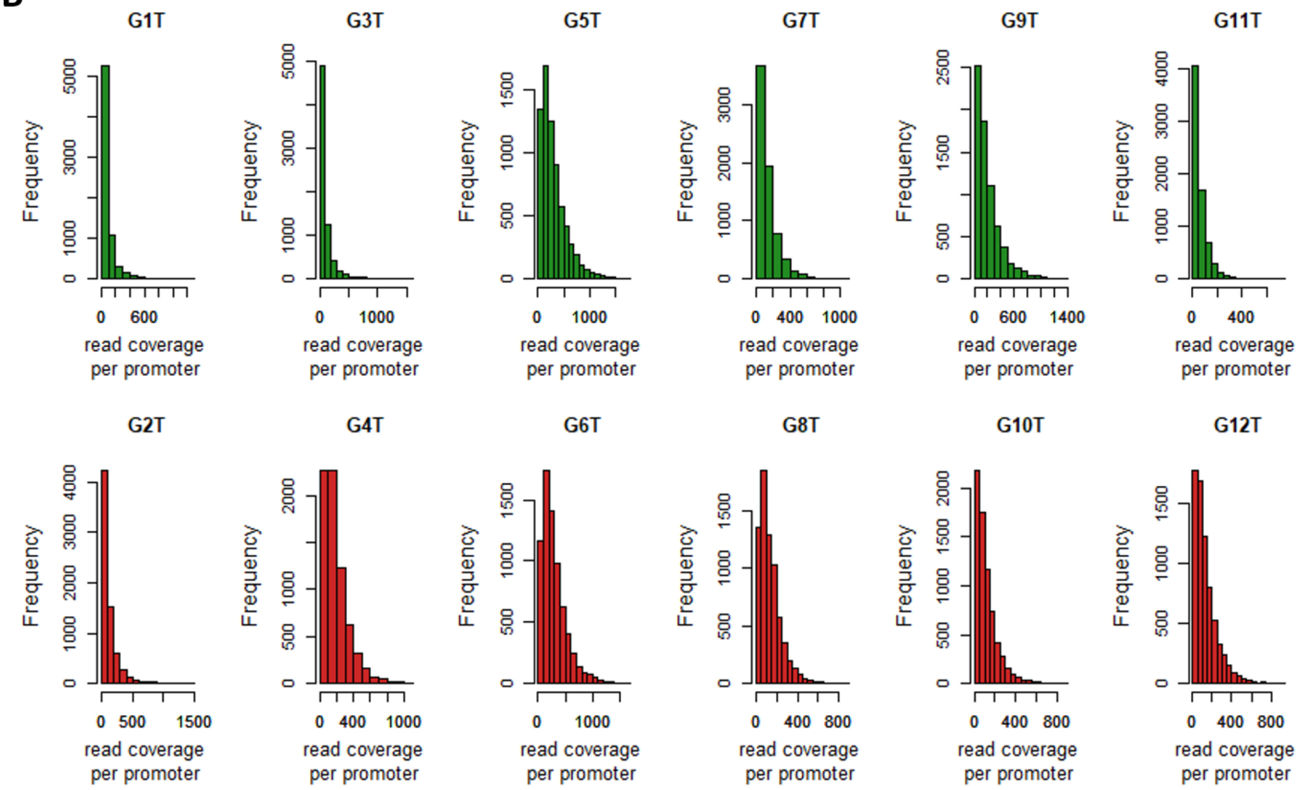


**Supplementary Figure 3.** (**A**) Methylation percentage- and (**B**) read coverage distribution per promoter for aortic (G1T, G3T, G5T, G7T, G9T, G11T) and mitral (G2T, G4T, G6T, G8T, G10T, G12T) tissue samples. The promoter percent methylation per sample exhibited a bimodal distribution, which is expected as reads of normal cells are either methylated (100%) or unmethylated (0%) (1). Promoter read coverage distribution did not show a secondary peak to the right, reflecting low PCR duplication levels (1). Both distributions reflect sufficient sample quality.

## 1.4 Figure S4. Boxplots of % methylation per sample, clustering, heatmap and PCA based on significant differential promoter methylation for aortic and mitral tissue samples

### (A) Boxplots of promoter % methylation per sample

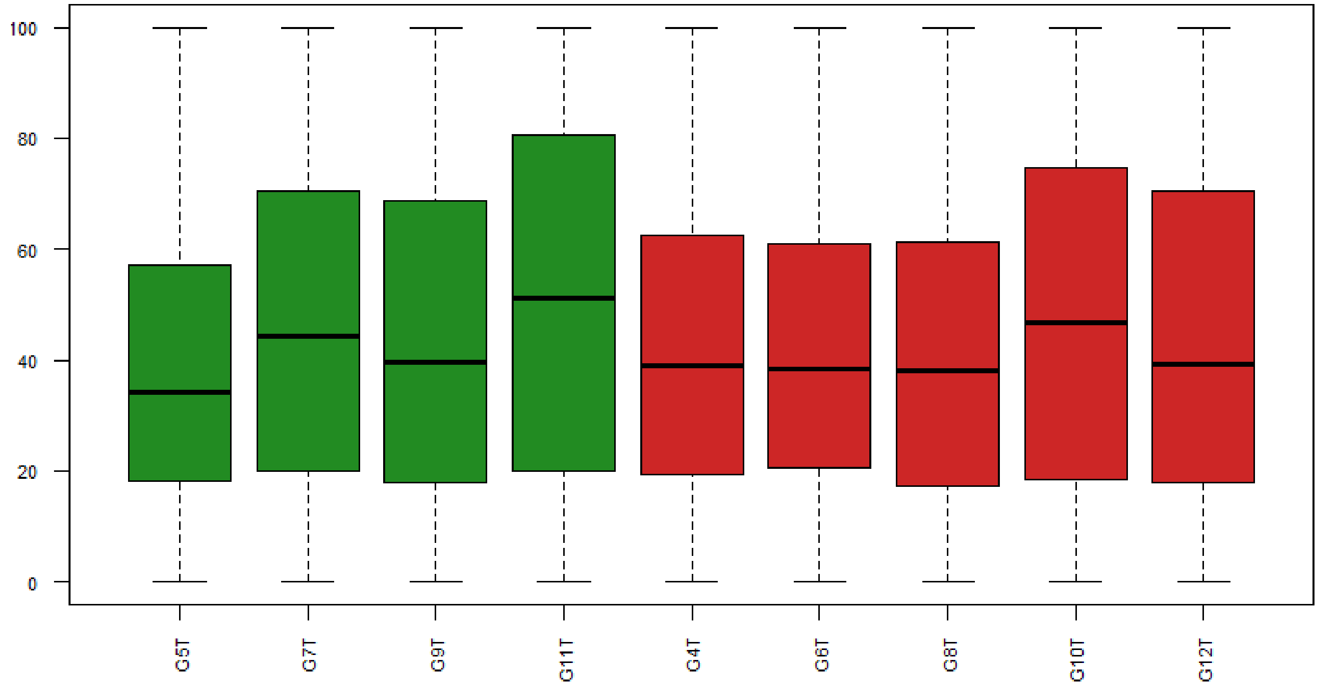


### (B) Clustering

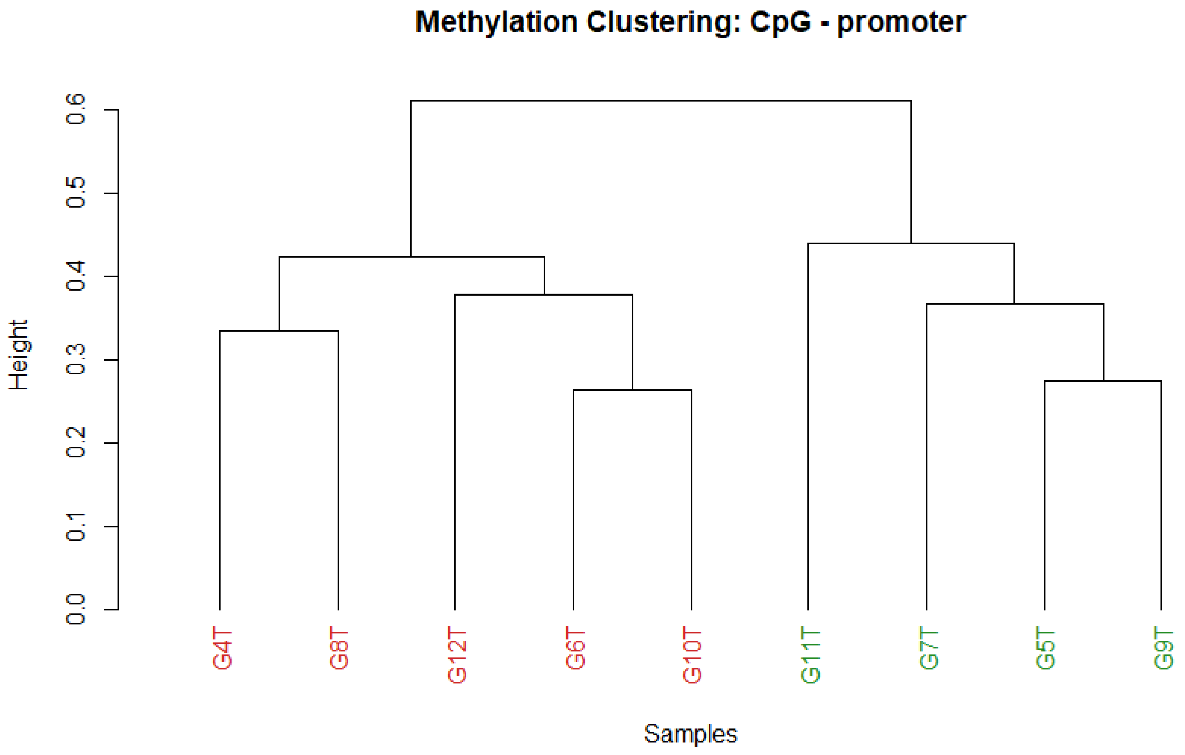

**(C) PCA**

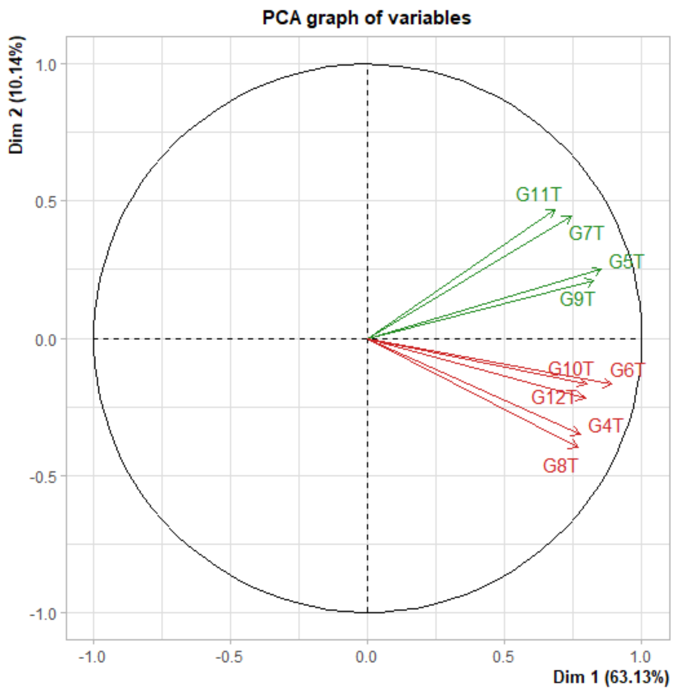


**(D) Heatmap**

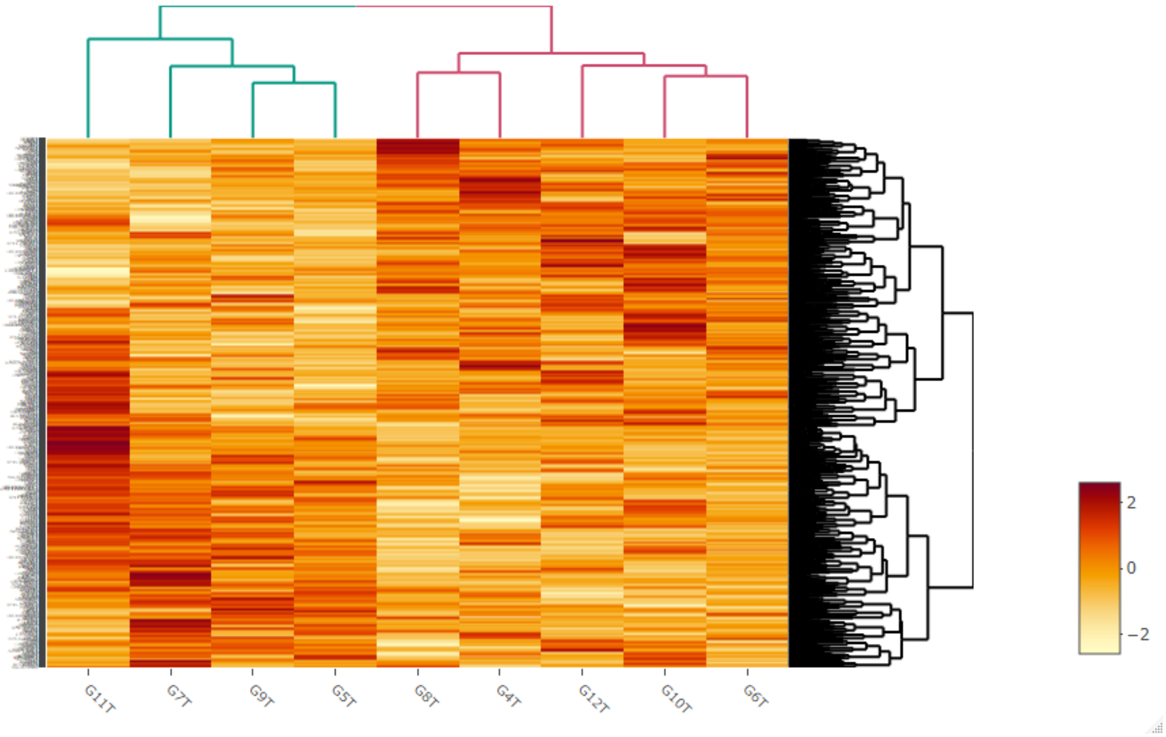


**Supplementary Figure 4.** (**A**) Boxplots, (**B**) Clustering, (**C**) PCA and (**D**) Heatmap analysis reflect the grouping of aortic and mitral tissue samples based on promoter percent methylation. Each row and column in the heatmap represent a gene linked to a significantly differentially methylated promoter

and sample. Samples and genes are clustered according to the correlation among the genes and among the samples, respectively. The percent methylation values of the promoters linked to the genes are color-coded and scaled for each gene (ie, by row). **Figures S3A-D** reflect meaningful sample grouping with aortic tissue samples (G5T, G7T, G9T and G11T) being clustered together and mitral tissue samples (G4T, G6T, G8T, G10T and G12T) being grouped together.

## 1.5 Figure S5. Additional functional categories that classify the genes associated with differentially methylated promoters in aortic vs. mitral tissue according to the PANTHER's Pathway, Molecular Function, Biological Process, Protein Class and Cellular Component annotation sets

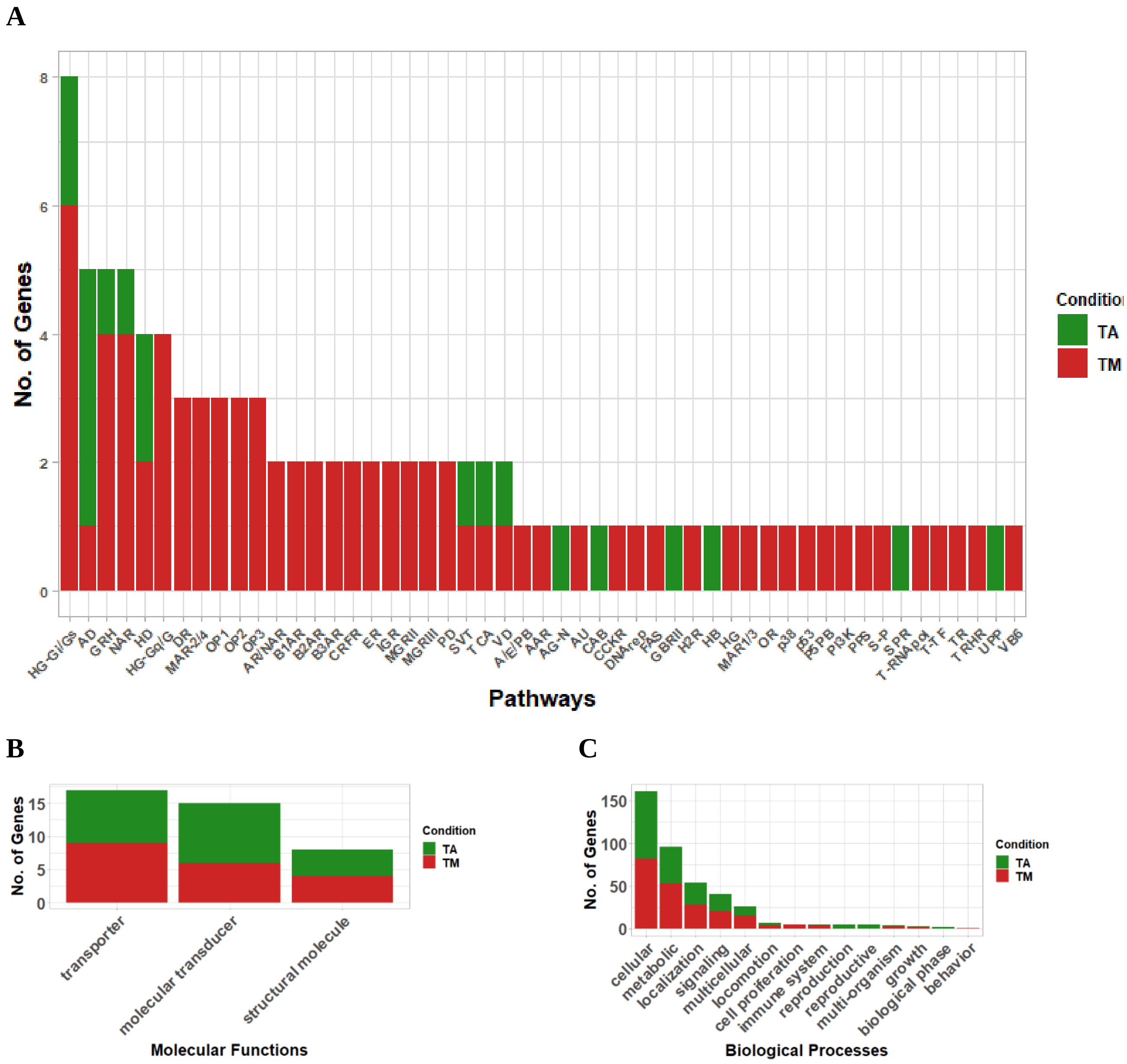

**D**

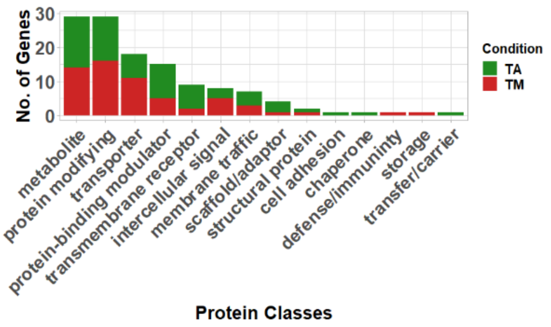


**E**

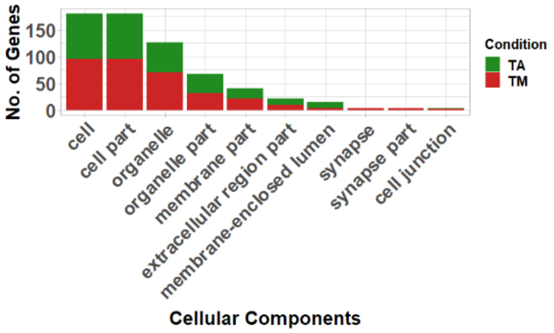


**Supplementary Figure 5.** Additional pathways/functions that result from the functional classification of genes that are associated with differentially methylated promoters (q-valuealue < 0.05 & meth.diff > |10|) between aortic and mitral tissue according to PANTHER (**A**) Pathways, (**B**) Molecular Functions, (**C**) Biological Processes, (**D**) Protein Classes and (**E**) Cellular Components. HG-Gi/Gs: "Heterotrimeric G-protein signaling pathway-Gi alpha and Gs alpha mediated pathway (P00026)"; AD: "Alzheimer disease-presenilin pathway (P00004)"; NAR: "Nicotinic acetylcholine receptor signaling pathway (P00044)"; GRH: "Gonadotropin-releasing hormone receptor pathway (P06664)"; HD: "Huntington disease (P00029)"; HG-Gq/G: "Heterotrimeric G-protein signaling pathway-Gq alpha and Go alpha mediated pathway (P00027)"; OP1: "Opioid proopiomelanocortin pathway (P05917)" ; OP2: "Opioid prodynorphin pathway (P05916)" ; OP3: "Opioid proenkephalin pathway (P05915)"; MAR-2/4: "Muscarinic acetylcholine receptor 2 and 4 signaling pathway (P00043)"; DR: "Dopamine receptor mediated signaling pathway (P05912)"; B3AR: "Beta3 adrenergic receptor signaling pathway (P04379)"; MGRIII: "Metabotropic glutamate receptor group III pathway (P00039)"; B2AR: "Beta2 adrenergic receptor signaling pathway (P04378)"; B1AR: "Beta1 adrenergic receptor signaling pathway (P04377)"; IGR: "Ionotropic glutamate receptor pathway (P00037)"; AR/NAR: "Adrenaline and noradrenaline biosynthesis (P00001)"; SVT: "Synaptic vesicle trafficking (P05734)"; VD: "Vitamin D metabolism and pathway (P04396)"; TCA: "T cell activation (P00053)"; PD: "Parkinson disease (P00049)"; ER: "Enkephalin release (P05913)"; CRFR: "Cortocotropin releasing factor receptor signaling pathway (P04380)"; MGRII: "Metabotropic glutamate receptor group II pathway (P00040)"; AG-N: "Axon guidance mediated by netrin (P00009)"; CAB: "Coenzyme A biosynthesis (P02736)"; AAR: "Alpha adrenergic receptor signaling pathway (P00002)"; UPP: "Ubiquitin proteasome pathway (P00060)"; GBRII: "GABA-B receptor II signaling (P05731)"; HG: "Heterotrimeric G-protein signaling pathway-rod outer segment phototransduction (P00028)"; P53: "p53 pathway feedback loops 2 (P04398)"; P5PB: "Pyridoxal-5-phosphate biosynthesis (P02759)"; A/E/PB: "Androgen/estrogene/progesterone biosynthesis (P02727)"; T-TF: "Transcription regulation by bZIP transcription factor (P00055)"; TRHR: "Thyrotropin-releasing hormone receptor signaling pathway (P04394)"; VB6: "Vitamin B6 metabolism (P02787)"; TR: "Toll receptor signaling pathway (P00054)"; T-RNApol: "General transcription by RNA polymerase I (P00022)"; AU: "Acetate utilization (P02722)"; OR: "Oxytocin receptor mediated signaling pathway (P04391)"; FAS: "FAS signaling pathway (P00020)"; p38: "p38 MAPK pathway (P05918)"; DNArep: "DNA replication (P00017)"; PI3K: "PI3 kinase pathway (P00048)"; H2R: "Histamine H2 receptor mediated signaling pathway (P04386)"; HB: "Heme biosynthesis (P02746)"; S-P: "Succinate to proprionate conversion (P02777)"; SPR: "Salvage pyrimidine ribonucleotides (P02775)"; MAR1/3: "Muscarinic acetylcholine receptor 1 and 3 signaling pathway (P00042)"; CCKR: "CCKR signaling map (P06959)"; PPS: "Pyridoxal phosphate salvage pathway (P02770)". In the stacked bar graphs, TA (green) labels genes with promoters that show increased methylation in the aortic compared to the mitral valves and TM

(red) shows genes whose promoters exhibit increased methylation in the mitral compared to the aortic valves.

## 1.6 Figure S6. Promoters assessed in unmatched versus matched comparisons

**Total**

**A**

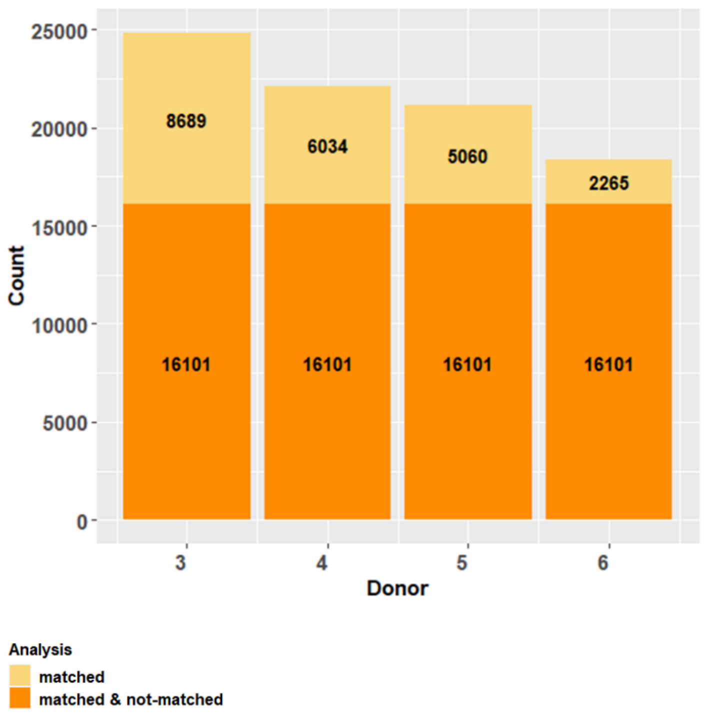

**By Chromosome**

B

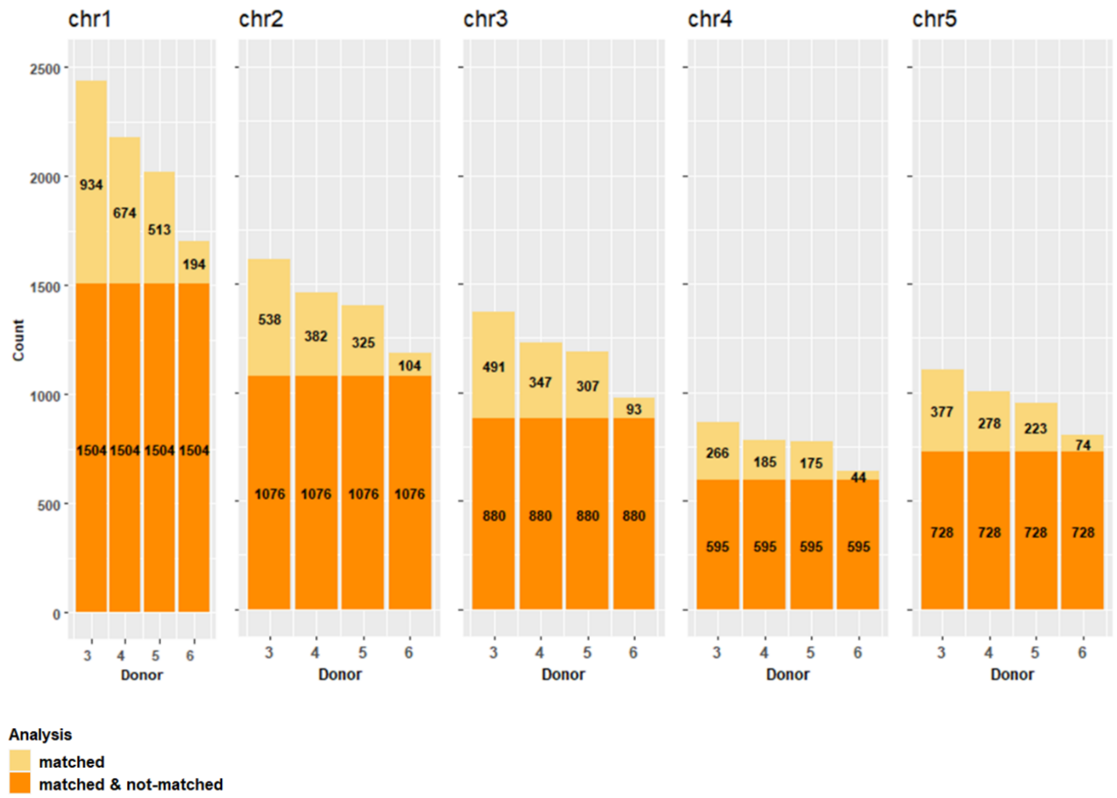


C

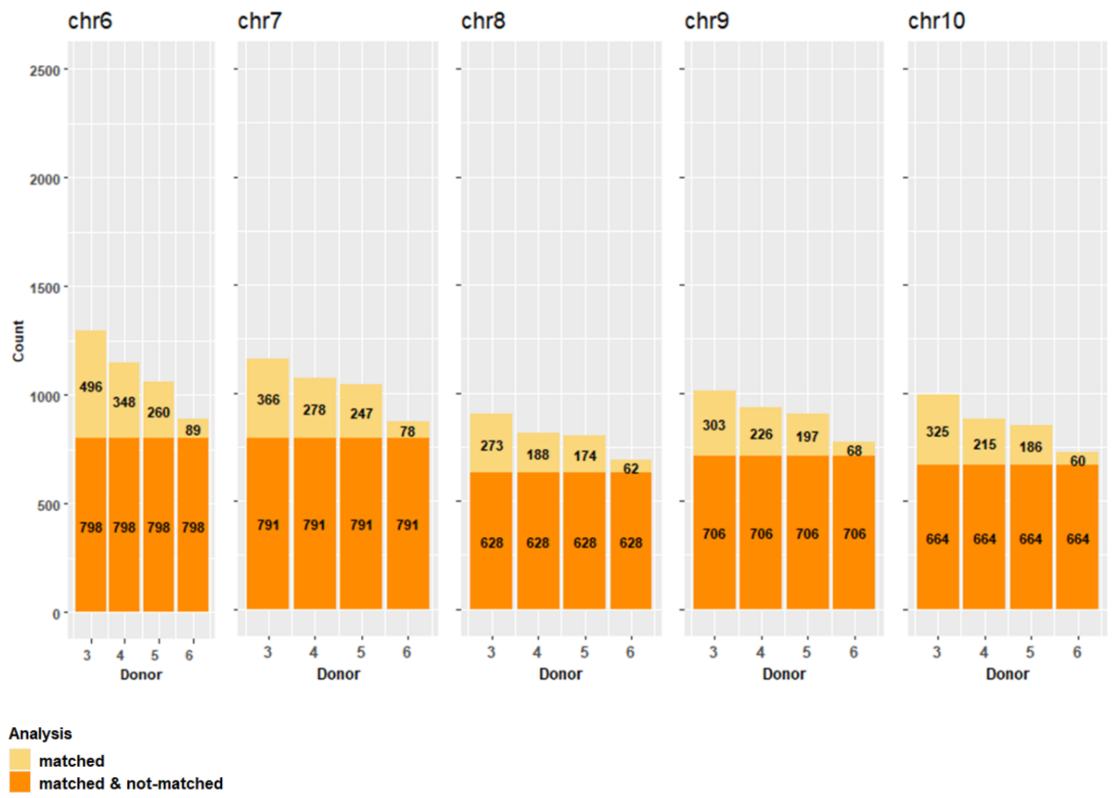

D

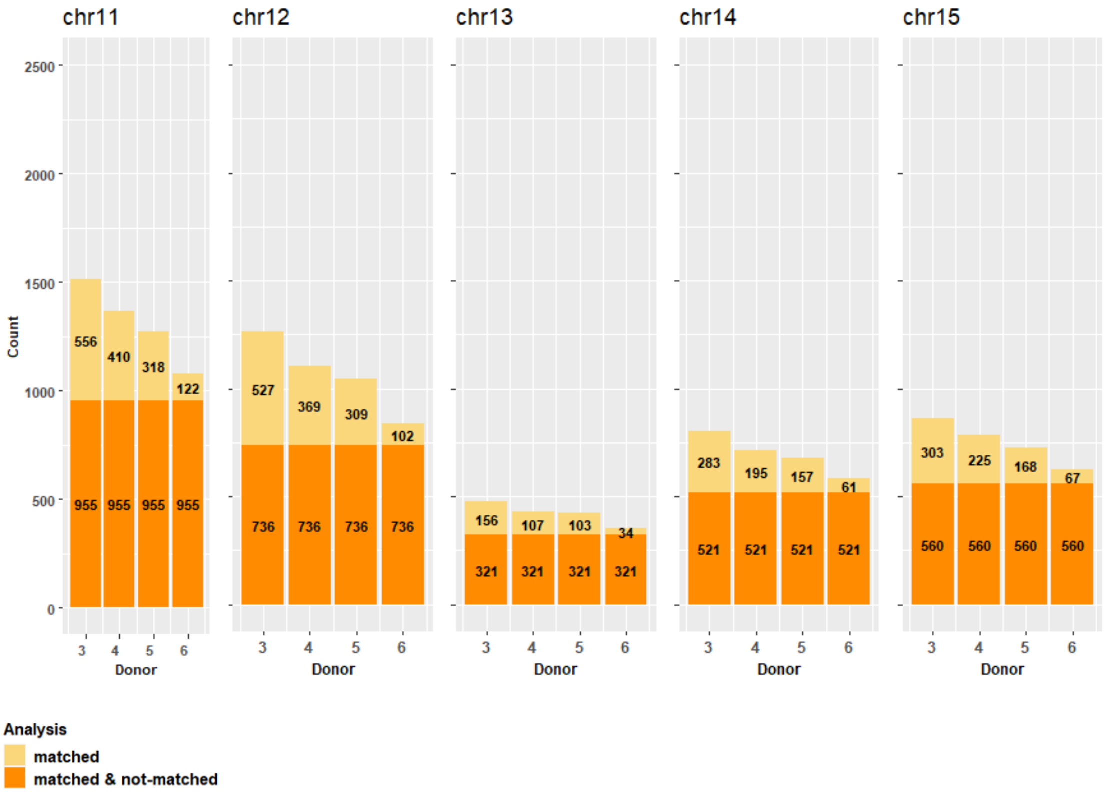
chr11
chr12
chr13
chr14
chr15
Count
2500
2000
1500
1000
500
0
556 410 318 122
955 955 955 955
527 369 309 102
736 736 736 736
156 107 103 34
321 321 321 321
283 195 157 61
521 521 521 521
303 225 168 67
560 560 560 560
3 4 5 6
Donor
Analysis
matched
matched & not-matched

E

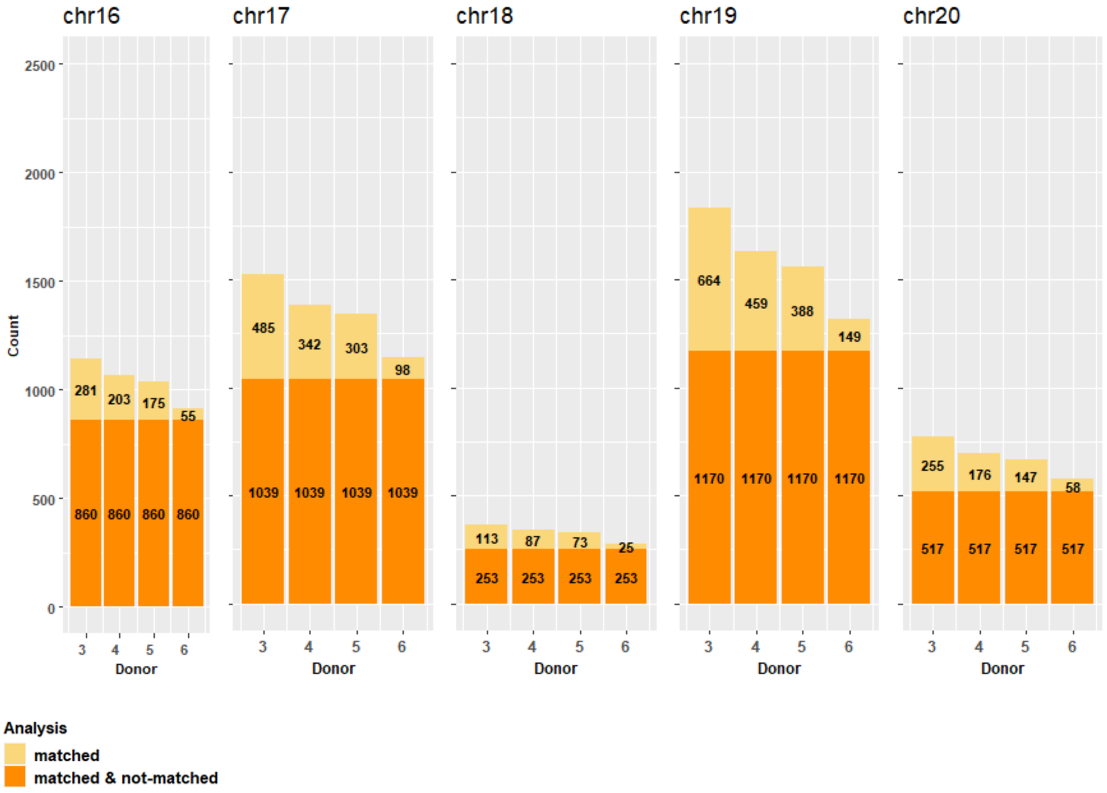
chr16
chr17
chr18
chr19
chr20
Count
2500
2000
1500
1000
500
0
281 203 175 55
860 860 860 860
485 342 303 98
1039 1039 1039 1039
113 87 73 25
253 253 253 253
664 459 388 149
1170 1170 1170 1170
255 176 147 58
517 517 517 517
3 4 5 6
Donor
Analysis
matched
matched & not-matched

**F**

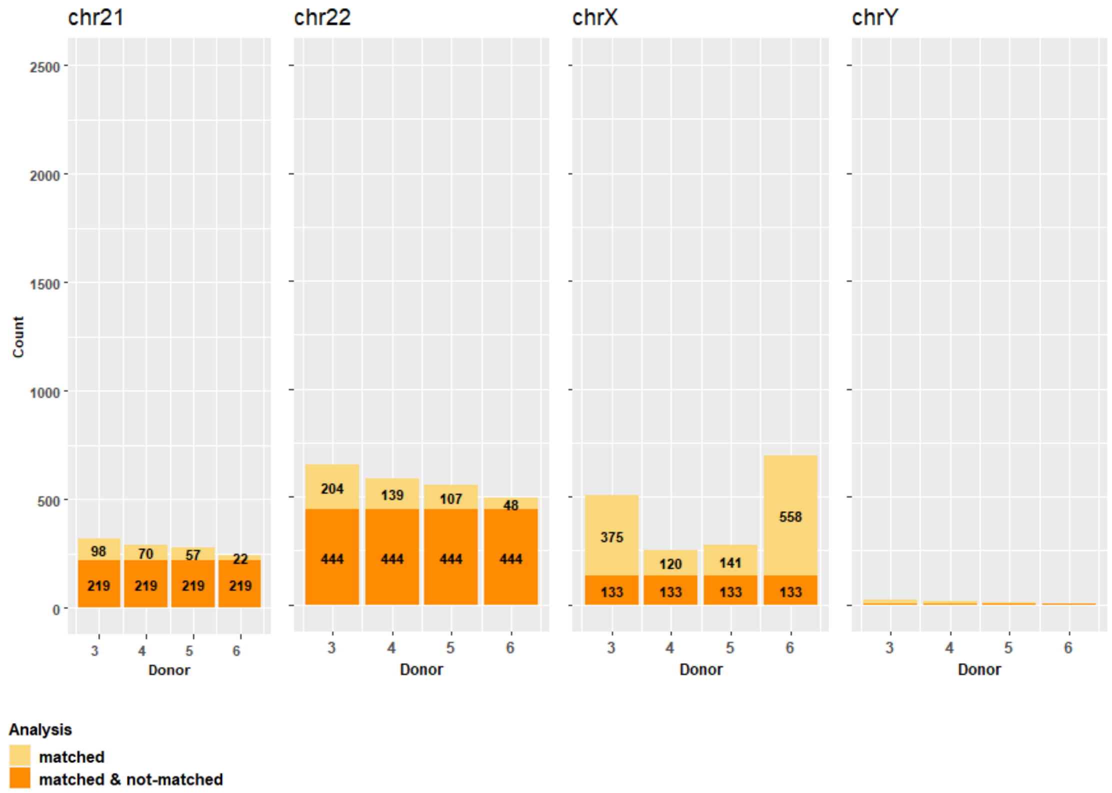


**Supplementary Figure 6.** Promoter count (A) total per donor and (B-F) total per chromosome per donor.

## 2 Supplementary Tables

### 2.1 Table S1. Differentially methylated promoters between aortic and mitral valve tissue (q value < 0.05 and a meth.diff > |10|) and their associated genes

| No | prom. chr | prom. start | prom. end | meth.diff | qvalue | assoc.gene-Symbol | gene.descr | dir |
|---|---|---|---|---|---|---|---|---|
| 1 | chr15 | 93616216 | 93618216 | 28.14311 | 1.66E-24 | RGMA | repulsive guidance molecule family member a | TM |
| 2 | chr6 | 121654646 | 121656646 | -41.2808 | 2.36E-18 | TBC1D32 | TBC1 domain family, member 32 | TA |
| 3 | chr19 | 45260913 | 45262913 | -22.2115 | 6.74E-16 | BCL3 | B-cell CLL/lymphoma 3 | TA |
| 4 | chr8 | 23081680 | 23083680 | -23.3939 | 1.13E-15 | RP11-1149O23.3 | lncRNA RP11-1149O23.3 | TA |
| 5 | chr8 | 23081733 | 23083733 | -23.3939 | 1.13E-15 | RP11-1149O23.3 | lncRNA RP11-1149O23.3 | TA |
| 6 | chr1 | 205818276 | 205820276 | 28.39292 | 2.66E-15 | PM20D1 | peptidase M20 domain containing 1 | TM |
| 7 | chr4 | 135121903 | 135123903 | 51.02311 | 5.10E-14 | PABPC4L | poly(A) binding protein, cytoplasmic 4-like | TM |
| 8 | chr4 | 1005251 | 1007251 | 16.31575 | 5.58E-14 | FGFRL1 | fibroblast growth factor receptor-like 1 | TM |
| 9 | chr7 | 65234797 | 65236797 | 29.14172 | 9.29E-14 | LOC441242 | Uncharacterized lncRNA LOC441242 | TM |
| 10 | chr5 | 28925976 | 28927976 | 22.90971 | 1.14E-12 | LSP1P3 | lymphocyte-specific protein 1 pseudogene 3 | TM |
| 11 | chr14 | 75421467 | 75423467 | 17.0601 | 1.14E-12 | PGF | placental growth factor | TM |
| 12 | chr2 | 106014575 | 106016575 | 20.47804 | 1.34E-12 | FHL2 | four and a half LIM domains 2 | TM |
| 13 | chr19 | 3061964 | 3063964 | 15.34849 | 1.44E-12 | AES | amino-terminal enhancer of split | TM |
| 14 | chr15 | 93615939 | 93617939 | 22.89901 | 1.64E-12 | RGMA | repulsive guidance molecule family member a | TM |
| 15 | chr2 | 106014681 | 106016681 | 19.94949 | 5.65E-12 | FHL2 | four and a half LIM domains 2 | TM |
| 16 | chr15 | 93615389 | 93617389 | 21.8204 | 8.55E-12 | RGMA | repulsive guidance molecule family member a | TM |
| 17 | chr3 | 57529071 | 57531071 | -54.9545 | 8.97E-12 | DNAH12 | dynein, axonemal, heavy chain 12 | TA |
| 18 | chr4 | 89298890 | 89300890 | -22.4831 | 8.97E-12 | HERC6 | HECT and RLD domain containing | TA |

| | | | | | | | | |
|---|---|---|---|---|---|---|---|---|
| | | | | | | | E3 ubiquitin protein ligase family member 6 | |
| 19 | chr17 | 62777117 | 62779117 | -25.8995 | 1.11E-11 | hsa-mir-6080 | hsa-mir-6080 | TA |
| 20 | chr12 | 113795370 | 113797370 | -17.4555 | 2.42E-11 | PLBD2 | phospholipase B domain containing 2 | TA |
| 21 | chr12 | 50355705 | 50357705 | -17.1681 | 2.84E-11 | LOC101927318 | Novel Transcript, Antisense To AQP5 | TA |
| 22 | chr12 | 50355707 | 50357707 | -17.1681 | 2.84E-11 | LOC101927318 | Novel Transcript, Antisense To AQP5 | TA |
| 23 | chr2 | 3621852 | 3623852 | -31.7708 | 2.96E-11 | RPS7 | ribosomal protein S7 | TA |
| 24 | chr17 | 37823233 | 37825233 | 16.11642 | 2.96E-11 | PNMT | phenylethanolamine N-methyltransferase | TM |
| 25 | chr7 | 131593978 | 131595978 | 17.91091 | 5.00E-11 | LOC101928782 | Uncharacterized lncRNA LOC101928782 | TM |
| 26 | chrX | 19904744 | 19906744 | -27.6015 | 6.95E-11 | SH3KBP1 | SH3-domain kinase binding protein 1 | TA |
| 27 | chr15 | 40530291 | 40532291 | -29.1746 | 7.01E-11 | PAK6 | p21 protein (Cdc42/Rac)-activated kinase 6 | TA |
| 28 | chr7 | 99868855 | 99870855 | 17.23618 | 7.78E-11 | GATS | CASTOR Family Member 3 | TM |
| 29 | chr7 | 154861033 | 154863033 | -15.1473 | 7.78E-11 | HTR5A | 5-hydroxytryptamine (serotonin) receptor 5A, G protein-coupled | TA |
| 30 | chr1 | 193074187 | 193076187 | 16.09079 | 1.15E-10 | GLRX2 | glutaredoxin 2 | TM |
| 31 | chr1 | 193074244 | 193076244 | 16.09079 | 1.15E-10 | GLRX2 | glutaredoxin 2 | TM |
| 32 | chr2 | 224701319 | 224703319 | -10.957 | 1.84E-10 | AP1S3 | adaptor-related protein complex 1, sigma 3 subunit | TA |
| 33 | chr9 | 95708600 | 95710600 | -27.3259 | 3.15E-10 | FGD3 | FYVE, RhoGEF and PH domain containing 3 | TA |
| 34 | chr21 | 15351765 | 15353765 | 23.3534 | 4.53E-10 | ANKRD20A11P | ankyrin repeat domain 20 family, member A11, pseudogene | TM |
| 35 | chr12 | 123463606 | 123465606 | 12.77897 | 4.94E-10 | ARL6IP4 | ADP-ribosylation-like factor 6 interacting protein 4 | TM |
| 36 | chr12 | 111050911 | 111052911 | 25.19245 | 7.93E-10 | TCTN1 | tectonic family member 1 | TM |
| 37 | chr20 | 45337278 | 45339278 | -17.9246 | 9.04E-10 | SLC2A10 | solute carrier family 2 (facilitated glucose transporter), member 10 | TA |

| | | | | | | | | |
|---|---|---|---|---|---|---|---|---|
| 38 | chr19 | 58873214 | 58875214 | -10.2772 | 1.30E-09 | ZNF497 | zinc finger protein 497 | TA |
| 39 | chr7 | 1125442 | 1127442 | -25.5795 | 4.25E-09 | GPER1 | G Protein-Coupled Estrogen Receptor 1 | TA |
| 40 | chr7 | 23570660 | 23572660 | 23.11953 | 4.84E-09 | TRA2A | transformer 2 alpha homolog (Drosophila) | TM |
| 41 | chr20 | 36792700 | 36794700 | 23.42375 | 4.84E-09 | TGM2 | transglutaminase 2 | TM |
| 42 | chr16 | 1582657 | 1584657 | 16.97891 | 5.56E-09 | TMEM204 | transmembrane protein 204 | TM |
| 43 | chr10 | 135339866 | 135341866 | 17.71944 | 5.67E-09 | CYP2E1 | cytochrome P450, family 2, subfamily E, polypeptide 1 | TM |
| 44 | chr13 | 52732670 | 52734670 | 11.21173 | 5.67E-09 | NEK3 | NIMA-related kinase 3 | TM |
| 45 | chr13 | 52732996 | 52734996 | 11.21173 | 5.67E-09 | NEK3 | NIMA-related kinase 3 | TM |
| 46 | chr15 | 40530997 | 40532997 | -21.4367 | 6.20E-09 | PAK6 | p21 protein (Cdc42/Rac)-activated kinase 6 | TA |
| 47 | chr19 | 58192336 | 58194336 | 17.70154 | 6.20E-09 | ZNF551 | zinc finger protein 551 | TM |
| 48 | chr3 | 13113617 | 13115617 | -15.2565 | 9.83E-09 | IQSEC1 | IQ motif and Sec7 domain 1 | TA |
| 49 | chr19 | 3800810 | 3802810 | -27.0836 | 1.10E-08 | MATK | megakaryocyte-associated tyrosine kinase | TA |
| 50 | chr12 | 111050831 | 111052831 | 23.61542 | 1.16E-08 | TCTN1 | tectonic family member 1 | TM |
| 51 | chr9 | 117348993 | 117350993 | -14.9785 | 1.35E-08 | ATP6V1G1 | ATPase, H+ transporting, lysosomal 13kDa, V1 subunit G1 | TA |
| 52 | chr19 | 19051041 | 19053041 | 10.61533 | 1.44E-08 | HOMER3 | homer homolog 3 (Drosophila) | TM |
| 53 | chr17 | 37823705 | 37825705 | 13.8831 | 1.95E-08 | PNMT | phenylethanolamine N-methyltransferase | TM |
| 54 | chr16 | 86611114 | 86613114 | 13.67516 | 2.52E-08 | FOXL1 | forkhead box L1 | TM |
| 55 | chr16 | 2847485 | 2849485 | 12.20137 | 3.13E-08 | PRSS41 | protease, serine, 41 | TM |
| 56 | chr4 | 1165999 | 1167999 | -17.0739 | 3.74E-08 | SPON2 | spondin 2, extracellular matrix protein | TA |
| 57 | chr16 | 1145244 | 1147244 | 13.73378 | 4.24E-08 | C1QTNF8 | C1q and tumor necrosis factor related protein 8 | TM |
| 58 | chr7 | 148717 | 150717 | -14.877 | 4.81E-08 | LOC100507642 | Uncharacterized ncRNA LOC100507642 | TA |

| | | | | | | | | |
|---|---|---|---|---|---|---|---|---|
| 59 | chr16 | 1727277 | 1729277 | 15.51686 | 5.44E-08 | HN1L | hematological and neurological expressed 1-like | TM |
| 60 | chr7 | 154862267 | 154864267 | -11.8912 | 6.55E-08 | HTR5A-AS1 | HTR5A antisense RNA 1 | TA |
| 61 | chr15 | 91497105 | 91499105 | -19.0998 | 1.12E-07 | RCCD1 | RCC1 domain containing 1 | TA |
| 62 | chr7 | 27160537 | 27162537 | 40.16618 | 1.19E-07 | HOXA-AS2 | HOXA cluster antisense RNA 2 | TM |
| 63 | chr16 | 89265529 | 89267529 | 22.03084 | 1.67E-07 | SLC22A31 | solute carrier family 22, member 31 | TM |
| 64 | chr17 | 80185281 | 80187281 | 13.40508 | 1.78E-07 | SLC16A3 | solute carrier family 16 (monocarboxylate transporter), member 3 | TM |
| 65 | chr1 | 149286450 | 149288450 | -15.861 | 2.23E-07 | LOC388692 | Uncharacterized LOC388692 (LOC388692), mRNA. | TA |
| 66 | chr5 | 178486606 | 178488606 | -17.1657 | 2.47E-07 | ZNF354C | zinc finger protein 354C | TA |
| 67 | chr11 | 63438446 | 63440446 | -12.325 | 2.94E-07 | ATL3 | atlastin GTPase 3 | TA |
| 68 | chr18 | 21976833 | 21978833 | 10.37672 | 3.14E-07 | OSBPL1A | oxysterol binding protein-like 1A | TM |
| 69 | chr20 | 62611430 | 62613430 | 22.41379 | 3.36E-07 | PRPF6 | pre-mRNA processing factor 6 | TM |
| 70 | chr11 | 62413198 | 62415198 | -14.0199 | 4.16E-07 | GANAB | glucosidase, alpha; neutral AB | TA |
| 71 | chr15 | 85196521 | 85198521 | 17.49581 | 5.71E-07 | WDR73 | WD repeat domain 73 | TM |
| 72 | chr11 | 124608828 | 124610828 | -19.3274 | 5.95E-07 | NRGN | neurogranin (protein kinase C substrate, RC3) | TA |
| 73 | chr13 | 76209130 | 76211130 | 12.25364 | 7.02E-07 | LMO7-AS1 | LMO7 Antisense RNA 1 | TM |
| 74 | chr17 | 7530194 | 7532194 | 27.33811 | 8.64E-07 | SHBG | sex hormone-binding globulin | TM |
| 75 | chr19 | 707952 | 709952 | 11.65544 | 1.11E-06 | PALM | paralemmin | TM |
| 76 | chrX | 107068083 | 107070083 | -15.7236 | 1.11E-06 | MID2 | midline 2 | TA |
| 77 | chr15 | 91446419 | 91448419 | 23.64103 | 1.14E-06 | MAN2A2 | mannosidase, alpha, class 2A, member 2 | TM |
| 78 | chr9 | 71735179 | 71737179 | 14.32164 | 1.15E-06 | TJP2 | tight junction protein 2 | TM |
| 79 | chr5 | 1633120 | 1635120 | -18.7866 | 1.28E-06 | LOC728613 | Programmed Cell Death 6 Pseudogene | TA |
| 80 | chr1 | 228352213 | 228354213 | 10.60424 | 1.46E-06 | IBA57 | IBA57, iron-sulfur cluster assembly | TM |

| | | | | | | | | |
|---|---|---|---|---|---|---|---|---|
| | | | | | | | homolog (S. cerevisiae) | |
| 81 | chr11 | 115629918 | 115631918 | 17.28773 | 1.47E-06 | LINC00900 | long intergenic non-protein coding RNA 900 | TM |
| 82 | chr17 | 79138306 | 79140306 | -12.2283 | 1.50E-06 | AATK-AS1 | AATK antisense RNA 1 | TA |
| 83 | chr2 | 85838225 | 85840225 | -19.2001 | 1.51E-06 | C2orf68 | chromosome 2 open reading frame 68 | TA |
| 84 | chr21 | 46828980 | 46830980 | 10.76715 | 1.75E-06 | COL18A1-AS2 | COL18A1 antisense RNA 2 | TM |
| 85 | chr11 | 63438202 | 63440202 | -10.8075 | 1.90E-06 | ATL3 | atlastin GTPase 3 | TA |
| 86 | chr20 | 57581309 | 57583309 | 12.42827 | 1.93E-06 | CTSZ | cathepsin Z | TM |
| 87 | chr17 | 44319929 | 44321929 | 17.53909 | 1.94E-06 | AC139677.11 | Homo sapiens chromosome 17, clone RP11-1070B7 | TM |
| 88 | chr1 | 149286129 | 149288129 | -14.6303 | 2.16E-06 | LOC388692 | Uncharacterized LOC388692 (LOC388692), mRNA. | TA |
| 89 | chr22 | 19703742 | 19705742 | 10.05661 | 2.46E-06 | SEPT5-GP1BB | CDNA FLJ60582, Highly Similar To Septin-5 | TM |
| 90 | chr22 | 46662860 | 46664860 | 14.57203 | 2.59E-06 | TTC38 | tetratricopeptide repeat domain 38 | TM |
| 91 | chr3 | 126112641 | 126114641 | 23.22841 | 2.72E-06 | RP11-71E19.5 | Uncharacterized antisense RNA LOC100506907 | TM |
| 92 | chr1 | 1368953 | 1370953 | -12.51 | 3.21E-06 | LINC01770 | Long Intergenic Non-Protein Coding RNA 1770 | TA |
| 93 | chr16 | 12009519 | 12011519 | 12.36027 | 3.36E-06 | GSPT1 | G1 to S phase transition 1 | TM |
| 94 | chr19 | 4182350 | 4184350 | -25.1634 | 3.36E-06 | SIRT6 | sirtuin 6 | TA |
| 95 | chr4 | 1165657 | 1167657 | -12.4642 | 3.39E-06 | SPON2 | spondin 2, extracellular matrix protein | TA |
| 96 | chr10 | 115437920 | 115439920 | 12.29278 | 3.44E-06 | CASP7 | caspase 7, apoptosis-related cysteine peptidase | TM |
| 97 | chr10 | 102789995 | 102791995 | -20.2387 | 3.49E-06 | PDZD7 | PDZ domain containing 7 | TA |
| 98 | chr7 | 150496853 | 150498853 | -13.2631 | 3.68E-06 | TMEM176B | transmembrane protein 176B | TA |
| 99 | chr4 | 122853268 | 122855268 | -10.1457 | 3.91E-06 | TRPC3 | transient receptor potential cation channel, subfamily C, member 3 | TA |
| 100 | chr10 | 52498687 | 52500687 | 15.99461 | 3.91E-06 | ASAH2B | N-acylsphingosine amidohydrolase | TM |

| | | | | | | | | |
|---|---|---|---|---|---|---|---|---|
| | | | | | | | (non-lysosomal ceramidase) 2B | |
| 101 | chr13 | 32888616 | 32890616 | 16.35677 | 3.91E-06 | BRCA2 | breast cancer 2, early onset | TM |
| 102 | chr1 | 24741244 | 24743244 | -24.5803 | 3.93E-06 | STPG1 | sperm-tail PG-rich repeat containing 1 | TA |
| 103 | chr7 | 99101272 | 99103272 | -22.365 | 4.17E-06 | ZKSCAN5 | zinc finger with KRAB and SCAN domains 5 | TA |
| 104 | chr5 | 170287895 | 170289895 | 10.46477 | 4.29E-06 | RANBP17 | RAN binding protein 17 | TM |
| 105 | chr12 | 50355566 | 50357566 | -12.3252 | 4.45E-06 | LOC101927318 | Novel Transcript, Antisense To AQP5 | TA |
| 106 | chr8 | 144416061 | 144418061 | 15.78471 | 4.52E-06 | TOP1MT | topoisomerase (DNA) I, mitochondrial | TM |
| 107 | chr1 | 12676737 | 12678737 | 11.88141 | 4.80E-06 | DHRS3 | dehydrogenase/reductase (SDR family) member 3 | TM |
| 108 | chr9 | 95819969 | 95821969 | 13.07512 | 5.40E-06 | SUSD3 | sushi domain containing 3 | TM |
| 109 | chr3 | 150420742 | 150422742 | 17.0898 | 5.44E-06 | ERICH6-AS1 | ERICH6 Antisense RNA 1 | TM |
| 110 | chr3 | 150420761 | 150422761 | 17.0898 | 5.44E-06 | ERICH6-AS1 | ERICH6 Antisense RNA 1 | TM |
| 111 | chr3 | 126112781 | 126114781 | 22.19131 | 5.55E-06 | RP11-71E19.5 | Uncharacterized antisense RNA LOC100506907 | TM |
| 112 | chr17 | 7530286 | 7532286 | 25.47182 | 5.93E-06 | SHBG | sex hormone-binding globulin | TM |
| 113 | chr4 | 111396228 | 111398228 | 15.28657 | 6.42E-06 | ENPEP | glutamyl aminopeptidase (aminopeptidase A) | TM |
| 114 | chr11 | 46401333 | 46403333 | 18.33024 | 6.42E-06 | MDK | midkine (neurite growth-promoting factor 2) | TM |
| 115 | chr11 | 18414935 | 18416935 | 14.12136 | 6.58E-06 | LDHA | lactate dehydrogenase A | TM |
| 116 | chr7 | 143058840 | 143060840 | -14.5264 | 6.80E-06 | FAM131B | family with sequence similarity 131, member B | TA |
| 117 | chr22 | 45704784 | 45706784 | -11.1274 | 6.85E-06 | FAM118A | family with sequence similarity 118, member A | TA |
| 118 | chr2 | 133173146 | 133175146 | 18.21649 | 7.04E-06 | GPR39 | G protein-coupled receptor 39 | TM |
| 119 | chr19 | 19728725 | 19730725 | -18.8354 | 7.34E-06 | PBX4 | pre-B-cell leukemia homeobox 4 | TA |

| | | | | | | | | |
|---|---|---|---|---|---|---|---|---|
| 120 | chr5 | 70882114 | 70884114 | 10.53042 | 7.88E-06 | MCCC2 | methylcrotonoyl-CoA carboxylase 2 (beta) | TM |
| 121 | chr15 | 96875568 | 96877568 | 13.56675 | 7.88E-06 | NR2F2 | nuclear receptor subfamily 2, group F, member 2 | TM |
| 122 | chr19 | 38754097 | 38756097 | -12.5734 | 7.88E-06 | SPINT2 | serine peptidase inhibitor, Kunitz type, 2 | TA |
| 123 | chr22 | 45704080 | 45706080 | -12.1701 | 7.88E-06 | FAM118A | family with sequence similarity 118, member A | TA |
| 124 | chrX | 152989201 | 152991201 | -12.6222 | 7.88E-06 | ABCD1 | ATP-binding cassette, sub-family D (ALD), member 1 | TA |
| 125 | chr2 | 96675298 | 96677298 | -27.0275 | 8.19E-06 | FAHD2CP | fumarylacetoacetate hydrolase domain containing 2C, pseudogene | TA |
| 126 | chr9 | 130711993 | 130713993 | -16.0597 | 8.34E-06 | FAM102A | family with sequence similarity 102, member A | TA |
| 127 | chr16 | 4302790 | 4304790 | -13.9405 | 8.42E-06 | RP11-95P2.1 | Uncharacterized lncRNA LOC100507501 | TA |
| 128 | chr19 | 29217601 | 29219601 | 14.7972 | 8.42E-06 | LOC100420587 | SHC Binding And Spindle Associated 1 Pseudogene | TM |
| 129 | chr12 | 124154659 | 124156659 | -19.7495 | 8.51E-06 | TCTN2 | tectonic family member 2 | TA |
| 130 | chr20 | 35723403 | 35725403 | 22.97564 | 9.46E-06 | RBL1 | retinoblastoma-like 1 (p107) | TM |
| 131 | chr16 | 56671577 | 56673577 | -15.9538 | 9.67E-06 | MT1A | metallothionein 1A | TA |
| 132 | chr5 | 131592350 | 131594350 | 12.58185 | 1.19E-05 | PDLIM4 | PDZ and LIM domain 4 | TM |
| 133 | chr4 | 128801015 | 128803015 | 19.96967 | 1.20E-05 | PLK4 | polo-like kinase 4 | TM |
| 134 | chr9 | 140171279 | 140173279 | -10.1032 | 1.23E-05 | TOR4A | torsin family 4, member A | TA |
| 135 | chr16 | 85721588 | 85723588 | -13.764 | 1.33E-05 | GINS2 | GINS complex subunit 2 (Psf2 homolog) | TA |
| 136 | chr22 | 42948867 | 42950867 | -11.0445 | 1.39E-05 | SERHL2 | serine hydrolase-like 2 | TA |
| 137 | chrX | 41781287 | 41783287 | 16.94494 | 1.53E-05 | CASK | calcium/calmodulin-dependent serine protein kinase (MAGUK family) | TM |
| 138 | chr15 | 96875489 | 96877489 | 11.04891 | 1.67E-05 | NR2F2 | nuclear receptor subfamily 2, group F, member 2 | TM |

| | | | | | | | | |
|---|---|---|---|---|---|---|---|---|
| 139 | chr13 | 114017463 | 114019463 | -11.8419 | 1.93E-05 | GRTP1 | growth hormone regulated TBC protein 1 | TA |
| 140 | chr1 | 244623672 | 244625672 | -20.1678 | 1.95E-05 | C1orf101 | chromosome 1 open reading frame 101 | TA |
| 141 | chr8 | 41385724 | 41387724 | -20.5911 | 1.98E-05 | GINS4 | GINS complex subunit 4 (Sld5 homolog) | TA |
| 142 | chr2 | 85838179 | 85840179 | -17.2037 | 2.19E-05 | C2orf68 | chromosome 2 open reading frame 68 | TA |
| 143 | chr20 | 56285592 | 56287592 | -31.0166 | 2.25E-05 | PMEPA1 | prostate transmembrane protein, androgen induced 1 | TA |
| 144 | chr16 | 68876508 | 68878508 | -24.1921 | 2.40E-05 | TANGO6 | transport and golgi organization 6 homolog (Drosophila) | TA |
| 145 | chr19 | 10120147 | 10122147 | 16.65573 | 2.40E-05 | COL5A3 | collagen, type V, alpha 3 | TM |
| 146 | chr17 | 74098652 | 74100652 | 15.42378 | 2.42E-05 | EXOC7 | exocyst complex component 7 | TM |
| 147 | chr14 | 75388145 | 75390145 | -15.4658 | 2.53E-05 | RPS6KL1 | ribosomal protein S6 kinase-like 1 | TA |
| 148 | chr6 | 88181642 | 88183642 | 12.37947 | 2.65E-05 | SLC35A1 | solute carrier family 35 (CMP-sialic acid transporter), member A1 | TM |
| 149 | chr15 | 90357072 | 90359072 | -17.3052 | 2.67E-05 | ANPEP | alanyl (membrane) aminopeptidase | TA |
| 150 | chr11 | 124628029 | 124630029 | 12.65104 | 2.88E-05 | LOC101929340 | Uncharacterized lncRNA LOC101929340 | TM |
| 151 | chr20 | 61582998 | 61584998 | 18.99397 | 2.88E-05 | SLC17A9 | solute carrier family 17 (vesicular nucleotide transporter), member 9 | TM |
| 152 | chr17 | 26552588 | 26554588 | -15.2789 | 3.27E-05 | PYY2 | peptide YY, 2 (pseudogene) | TA |
| 153 | chr15 | 89181038 | 89183038 | 26.67957 | 3.29E-05 | ISG20 | interferon stimulated exonuclease gene 20kDa | TM |
| 154 | chr3 | 73045118 | 73047118 | 14.40733 | 3.57E-05 | PPP4R2 | protein phosphatase 4, regulatory subunit 2 | TM |
| 155 | chr22 | 18592452 | 18594452 | 10.36429 | 3.63E-05 | TUBA8 | tubulin, alpha 8 | TM |
| 156 | chr18 | 13640756 | 13642756 | -10.4618 | 3.72E-05 | LDLRAD4 | low density lipoprotein receptor | TA |

| | | | | | | | | |
|---|---|---|---|---|---|---|---|---|
| | | | | | | | class A domain containing 4 | |
| 157 | chr14 | 24657124 | 24659124 | 12.19301 | 3.78E-05 | IPO4 | importin 4 | TM |
| 158 | chr5 | 76010867 | 76012867 | -10.0456 | 3.95E-05 | F2R | coagulation factor II (thrombin) receptor | TA |
| 159 | chr11 | 64862586 | 64864586 | 18.47006 | 3.98E-05 | VPS51 | vacuolar protein sorting 51 homolog (S. cerevisiae) | TM |
| 160 | chr20 | 47803904 | 47805904 | 13.74715 | 4.33E-05 | STAU1 | staufen double-stranded RNA binding protein 1 | TM |
| 161 | chr14 | 23425351 | 23427351 | 17.86834 | 4.37E-05 | MIR4707 | microRNA 4707 | TM |
| 162 | chr1 | 10092040 | 10094040 | 14.35139 | 4.79E-05 | UBE4B | ubiquitination factor E4B | TM |
| 163 | chr3 | 139107522 | 139109522 | 18.85098 | 5.08E-05 | LOC100507291 | Uncharacterized lncRNA LOC100507291 | TM |
| 164 | chr3 | 139107644 | 139109644 | 18.85098 | 5.08E-05 | LOC100507291 | Uncharacterized lncRNA LOC100507291 | TM |
| 165 | chr17 | 40713091 | 40715091 | -19.2769 | 5.22E-05 | COASY | CoA synthase | TA |
| 166 | chr10 | 73610082 | 73612082 | -20.4424 | 5.42E-05 | PSAP | prosaposin | TA |
| 167 | chr10 | 134777793 | 134779793 | -12.021 | 5.47E-05 | LINC01166 | long intergenic non-protein coding RNA 1166 | TA |
| 168 | chr16 | 14396823 | 14398823 | -20.0739 | 5.54E-05 | MIR193B | microRNA 193b | TA |
| 169 | chr19 | 40323873 | 40325873 | 15.87205 | 5.56E-05 | DYRK1B | dual-specificity tyrosine-(Y)-phosphorylation regulated kinase 1B | TM |
| 170 | chr17 | 38170613 | 38172613 | -30.7312 | 5.71E-05 | CSF3 | colony stimulating factor 3 (granulocyte) | TA |
| 171 | chr2 | 201449730 | 201451730 | -17.8466 | 6.10E-05 | AOX1 | aldehyde oxidase 1 | TA |
| 172 | chr19 | 49519347 | 49521347 | -15.5705 | 6.10E-05 | LHB | luteinizing hormone beta polypeptide | TA |
| 173 | chr17 | 46017770 | 46019770 | 16.87888 | 6.43E-05 | PNPO | pyridoxamine 5'-phosphate oxidase | TM |
| 174 | chr17 | 46017888 | 46019888 | 16.87888 | 6.43E-05 | PNPO | pyridoxamine 5'-phosphate oxidase | TM |
| 175 | chr9 | 132082294 | 132084294 | -19.5545 | 6.82E-05 | C9orf106 | chromosome 9 open reading frame 106 | TA |
| 176 | chr12 | 8379214 | 8381214 | 19.62302 | 6.87E-05 | FAM90A1 | family with sequence similarity 90, member A1 | TM |
| 177 | chr22 | 45808500 | 45810500 | -13.5464 | 7.61E-05 | RIBC2 | RIB43A domain with coiled-coils 2 | TA |
| 178 | chr8 | 22959326 | 22961326 | 16.4231 | 8.36E-05 | TNFRSF10C | tumor necrosis factor receptor | TM |

| | | | | | | | | |
|---|---|---|---|---|---|---|---|---|
| | | | | | | | superfamily, member 10c, decoy without an intracellular domain | |
| 179 | chr16 | 68032421 | 68034421 | -19.6122 | 9.29E-05 | DPEP2 | dipeptidase 2 | TA |
| 180 | chr19 | 10542110 | 10544110 | -18.6557 | 9.33E-05 | PDE4A | phosphodiesterase 4A, cAMP-specific | TA |
| 181 | chr1 | 20125410 | 20127410 | 13.29795 | 9.47E-05 | TMCO4 | transmembrane and coiled-coil domains 4 | TM |
| 182 | chr2 | 71679752 | 71681752 | -12.6866 | 9.47E-05 | DYSF | dysferlin | TA |
| 183 | chr12 | 6579065 | 6581065 | 12.39162 | 9.92E-05 | VAMP1 | vesicle-associated membrane protein 1 (synaptobrevin 1) | TM |
| 184 | chr6 | 31238913 | 31240913 | 14.75902 | 0.000105 | HLA-C | major histocompatibility complex, class I, C | TM |
| 185 | chr22 | 18957010 | 18959010 | -11.4021 | 0.000111 | DGCR5 | DiGeorge syndrome critical region gene 5 (non-protein coding) | TA |
| 186 | chr1 | 12078298 | 12080298 | 23.4642 | 0.000113 | MIIP | migration and invasion inhibitory protein | TM |
| 187 | chr10 | 102026437 | 102028437 | -17.8845 | 0.000117 | CWF19L1 | CWF19-like 1, cell cycle control (S. pombe) | TA |
| 188 | chr19 | 55677090 | 55679090 | -14.7451 | 0.000118 | DNAAF3 | dynein, axonemal, assembly factor 3 | TA |
| 189 | chr19 | 47633079 | 47635079 | -19.1342 | 0.00012 | SAE1 | SUMO1 activating enzyme subunit 1 | TA |
| 190 | chr19 | 47633114 | 47635114 | -19.1342 | 0.00012 | SAE1 | SUMO1 activating enzyme subunit 1 | TA |
| 191 | chr17 | 42091345 | 42093345 | -19.3123 | 0.000122 | TMEM101 | transmembrane protein 101 | TA |
| 192 | chr2 | 98329030 | 98331030 | 12.89195 | 0.000123 | ZAP70 | zeta-chain (TCR) associated protein kinase 70kDa | TM |
| 193 | chrX | 152952751 | 152954751 | 11.05677 | 0.000127 | SLC6A8 | solute carrier family 6 (neurotransmitter transporter), member 8 | TM |
| 194 | chrX | 153714009 | 153716009 | 13.04122 | 0.00013 | UBL4A | ubiquitin-like 4A | TM |
| 195 | chr16 | 85169755 | 85171755 | -16.6253 | 0.000138 | LINC02139 | Long Intergenic Non-Protein Coding RNA 2139 | TA |
| 196 | chr16 | 121629 | 123629 | 10.29471 | 0.000141 | RHBDF1 | rhomboid 5 homolog 1 (Drosophila) | TM |

| | | | | | | | | |
|---|---|---|---|---|---|---|---|---|
| 197 | chr12 | 50343523 | 50345523 | -21.134 | 0.000142 | AQP2 | aquaporin 2 (collecting duct) | TA |
| 198 | chr19 | 11545603 | 11547603 | -23.8105 | 0.000142 | PRKCSH | protein kinase C substrate 80K-H | TA |
| 199 | chr1 | 18956499 | 18958499 | -11.7985 | 0.000148 | PAX7 | paired box 7 | TA |
| 200 | chr18 | 43305917 | 43307917 | -19.1652 | 0.000149 | SLC14A1 | solute carrier family 14 (urea transporter), member 1 (Kidd blood group) | TA |
| 201 | chr19 | 52226245 | 52228245 | -22.1536 | 0.00015 | HAS1 | hyaluronan synthase 1 | TA |
| 202 | chr4 | 2365604 | 2367604 | 11.95305 | 0.00016 | ZFYVE28 | zinc finger, FYVE domain containing 28 | TM |
| 203 | chr2 | 197503355 | 197505355 | 14.84426 | 0.000161 | CCDC150 | coiled-coil domain containing 150 | TM |
| 204 | chr8 | 21910080 | 21912080 | -20.3027 | 0.000161 | DMTN | dematin actin binding protein | TA |
| 205 | chr17 | 77680074 | 77682074 | 11.94744 | 0.000166 | CTD-2116F7.1 | Long Intergenic Non-Protein Coding RNA 2078 | TM |
| 206 | chr22 | 45808571 | 45810571 | -12.9984 | 0.000178 | RIBC2 | RIB43A domain with coiled-coils 2 | TA |
| 207 | chr17 | 39596596 | 39598596 | 18.20972 | 0.000184 | KRT38 | keratin 38 | TM |
| 208 | chrX | 152989322 | 152991322 | -10.2904 | 0.000192 | ABCD1 | ATP-binding cassette, sub-family D (ALD), member 1 | TA |
| 209 | chr15 | 44068293 | 44070293 | 10.78098 | 0.000216 | SERF2 | small EDRK-rich factor 2 | TM |
| 210 | chr3 | 170587045 | 170589045 | -18.7738 | 0.000232 | RPL22L1 | ribosomal protein L22-like 1 | TA |
| 211 | chr16 | 34403762 | 34405762 | 14.08228 | 0.000235 | UBE2MP1 | ubiquitin-conjugating enzyme E2M pseudogene 1 | TM |
| 212 | chr19 | 55590759 | 55592759 | -10.982 | 0.000245 | EPS8L1 | EPS8-like 1 | TA |
| 213 | chr2 | 238767186 | 238769186 | -11.374 | 0.000249 | RAMP1 | receptor (G protein-coupled) activity modifying protein 1 | TA |
| 214 | chr19 | 52955828 | 52957828 | 32.07625 | 0.000249 | ZNF578 | zinc finger protein 578 | TM |
| 215 | chr14 | 101520023 | 101522023 | 19.92582 | 0.000258 | MIR485 | microRNA 485 | TM |
| 216 | chr1 | 161067150 | 161069150 | -10.4153 | 0.000265 | KLHDC9 | kelch domain containing 9 | TA |
| 217 | chr1 | 161067180 | 161069180 | -10.4153 | 0.000265 | KLHDC9 | kelch domain containing 9 | TA |
| 218 | chr1 | 24512765 | 24514765 | -13.2818 | 0.000267 | IFNLR1 | interferon, lambda receptor 1 | TA |

| | | | | | | | | |
|---|---|---|---|---|---|---|---|---|
| 219 | chr17 | 77680058 | 77682058 | 11.37794 | 0.00027 | CTD-2116F7.1 | Long Intergenic Non-Protein Coding RNA 2078 | TM |
| 220 | chr6 | 143832020 | 143834020 | 11.09653 | 0.000275 | FUCA2 | fucosidase, alpha-L-2, plasma | TM |
| 221 | chr7 | 135346220 | 135348220 | 14.94687 | 0.000276 | C7orf73 | chromosome 7 open reading frame 73 | TM |
| 222 | chr7 | 100463949 | 100465949 | -13.5053 | 0.000283 | TRIP6 | thyroid hormone receptor interactor 6 | TA |
| 223 | chr14 | 101519642 | 101521642 | 19.66746 | 0.000307 | MIR668 | MicroRNA 668 | TM |
| 224 | chr19 | 49989810 | 49991810 | -12.3628 | 0.000308 | RPL13A | ribosomal protein L13a | TA |
| 225 | chrX | 153638876 | 153640876 | -15.9405 | 0.000311 | DNASE1L1 | deoxyribonuclease I-like 1 | TA |
| 226 | chrX | 153639427 | 153641427 | -15.9405 | 0.000311 | DNASE1L1 | deoxyribonuclease I-like 1 | TA |
| 227 | chr10 | 70479900 | 70481900 | -10.1892 | 0.000313 | CCAR1 | cell division cycle and apoptosis regulator 1 | TA |
| 228 | chr11 | 67209983 | 67211983 | -16.6003 | 0.000313 | CORO1B | coronin, actin binding protein, 1B | TA |
| 229 | chr17 | 63132455 | 63134455 | 14.68599 | 0.000331 | RGS9 | regulator of G-protein signaling 9 | TM |
| 230 | chr19 | 2014702 | 2016702 | 14.78213 | 0.000335 | BTBD2 | BTB (POZ) domain containing 2 | TM |
| 231 | chr19 | 55314066 | 55316066 | 13.07365 | 0.000342 | KIR2DL4 | killer cell immunoglobulin-like receptor, two domains, long cytoplasmic tail, 4 | TM |
| 232 | chr12 | 114844999 | 114846999 | 17.03896 | 0.000346 | TBX5-AS1 | TBX5 antisense RNA 1 | TM |
| 233 | chr14 | 23651869 | 23653869 | -40.7436 | 0.000353 | SLC7A8 | solute carrier family 7 (amino acid transporter light chain, L system), member 8 | TA |
| 234 | chr1 | 6549640 | 6551640 | 10.6383 | 0.000355 | PLEKHG5 | pleckstrin homology domain containing, family G (with RhoGef domain) member 5 | TM |
| 235 | chr1 | 6549654 | 6551654 | 10.6383 | 0.000355 | PLEKHG5 | pleckstrin homology domain containing, family G (with RhoGef domain) member 5 | TM |
| 236 | chr14 | 23425238 | 23427238 | 15.50245 | 0.000357 | MIR4707 | microRNA 4707 | TM |
| 237 | chr5 | 169723822 | 169725822 | -24.2105 | 0.000359 | LCP2 | lymphocyte cytosolic protein 2 | TA |

| | | | | | | | | |
|---|---|---|---|---|---|---|---|---|
| | | | | | | | (SH2 domain containing leukocyte protein of 76kDa) | |
| 238 | chr17 | 1181956 | 1183956 | 12.28963 | 0.000361 | TUSC5 | tumor suppressor candidate 5 | TM |
| 239 | chr14 | 103654365 | 103656365 | -15.0979 | 0.000417 | LINC00605 | long intergenic non-protein coding RNA 605 | TA |
| 240 | chr19 | 1131273 | 1133273 | 11.57252 | 0.000423 | SBNO2 | strawberry notch homolog 2 (Drosophila) | TM |
| 241 | chr3 | 50272588 | 50274588 | 16.0433 | 0.000442 | GNAI2 | Guanine Nucleotide Binding Protein (G Protein), Alpha Inhibiting Activity | TM |
| 242 | chr1 | 220219000 | 220221000 | -11.5031 | 0.000444 | EPRS | glutamyl-prolyl-tRNA synthetase | TA |
| 243 | chr19 | 56047435 | 56049435 | -14.1007 | 0.000445 | SBK2 | SH3 domain binding kinase family, member 2 | TA |
| 244 | chr15 | 52262998 | 52264998 | 14.16123 | 0.000474 | LEO1 | Leo1, Paf1/RNA polymerase II complex component, homolog (S. cerevisiae) | TM |
| 245 | chr6 | 30850860 | 30852860 | 24.4129 | 0.00051 | DDR1 | discoidin domain receptor tyrosine kinase 1 | TM |
| 246 | chr7 | 155325539 | 155327539 | 10.92188 | 0.000519 | CNPY1 | canopy FGF signaling regulator 1 | TM |
| 247 | chr11 | 67170383 | 67172383 | 22.5974 | 0.000521 | TBC1D10C | TBC1 domain family, member 10C | TM |
| 248 | chr17 | 73779688 | 73781688 | -15.3648 | 0.000554 | UNK | unkempt family zinc finger | TA |
| 249 | chr17 | 16119508 | 16121508 | -19.4778 | 0.000595 | PIGL | phosphatidylinositol glycan anchor biosynthesis, class L | TA |
| 250 | chr3 | 195942382 | 195944382 | 14.35947 | 0.000608 | SLC51A | solute carrier family 51, alpha subunit | TM |
| 251 | chr13 | 113776112 | 113778112 | -13.1739 | 0.000612 | F10 | coagulation factor X | TA |
| 252 | chr17 | 56768933 | 56770933 | -13.9093 | 0.000687 | RAD51C | RAD51 paralog C | TA |
| 253 | chr19 | 49338934 | 49340934 | 12.72056 | 0.000697 | HSD17B14 | hydroxysteroid (17-beta) dehydrogenase 14 | TM |
| 254 | chr20 | 44538633 | 44540633 | -21.9858 | 0.000732 | PLTP | phospholipid transfer protein | TA |
| 255 | chr3 | 46734194 | 46736194 | 10.70251 | 0.000734 | ALS2CL | ALS2 C-terminal like | TM |

| | | | | | | | | |
|---|---|---|---|---|---|---|---|---|
| 256 | chrX | 54521599 | 54523599 | 13.42415 | 0.000739 | FGD1 | FYVE, RhoGEF and PH domain containing 1 | TM |
| 257 | chr4 | 154124565 | 154126565 | -22.5228 | 0.000747 | TRIM2 | tripartite motif containing 2 | TA |
| 258 | chr11 | 63257680 | 63259680 | -15.3228 | 0.000749 | HRASLS5 | HRAS-like suppressor family, member 5 | TA |
| 259 | chr17 | 75446325 | 75448325 | 11.70022 | 0.000772 | 9-Sep | septin 9 | TM |
| 260 | chr6 | 6003277 | 6005277 | 10.22646 | 0.000801 | NRN1 | neuritin 1 | TM |
| 261 | chr1 | 28695092 | 28697092 | 14.90113 | 0.000818 | PHACTR4 | phosphatase and actin regulator 4 | TM |
| 262 | chr7 | 65337256 | 65339256 | 12.57696 | 0.000818 | VKORC1L1 | vitamin K epoxide reductase complex, subunit 1-like 1 | TM |
| 263 | chr1 | 27960727 | 27962727 | 10.67886 | 0.000831 | FGR | feline Gardner-Rasheed sarcoma viral oncogene homolog | TM |
| 264 | chr20 | 3139556 | 3141556 | -17.923 | 0.000845 | UBOX5 | U-box domain containing 5 | TA |
| 265 | chr17 | 73779919 | 73781919 | -14.9291 | 0.000869 | UNK | unkempt family zinc finger | TA |
| 266 | chr3 | 150420350 | 150422350 | 16.12591 | 0.000878 | ERICH6-AS1 | ERICH6 Antisense RNA 1 | TM |
| 267 | chr16 | 2891752 | 2893752 | 10.85568 | 0.000927 | PRSS30P | protease, serine, 30, pseudogene | TM |
| 268 | chr20 | 3761102 | 3763102 | 24.88764 | 0.00094 | SPEF1 | sperm flagellar 1 | TM |
| 269 | chr1 | 36548675 | 36550675 | 17.63048 | 0.000971 | TEKT2 | tektin 2 (testicular) | TM |
| 270 | chr4 | 466998 | 468998 | 19.93379 | 0.000972 | ABCA11P | ATP-binding cassette, sub-family A (ABC1), member 11, pseudogene | TM |
| 271 | chr19 | 11545560 | 11547560 | -21.2864 | 0.000972 | PRKCSH | protein kinase C substrate 80K-H | TA |
| 272 | chr17 | 74098868 | 74100868 | 12.58592 | 0.000973 | EXOC7 | exocyst complex component 7 | TM |
| 273 | chr2 | 27885337 | 27887337 | -11.8543 | 0.000974 | SUPT7L | suppressor of Ty 7 (S. cerevisiae)-like | TA |
| 274 | chr3 | 127293174 | 127295174 | 13.06528 | 0.001045 | MIR6825 | MicroRNA 6825 | TM |
| 275 | chr19 | 35772409 | 35774409 | 28.14539 | 0.001053 | HAMP | hepcidin antimicrobial peptide | TM |
| 276 | chr22 | 17488112 | 17490112 | 12.51904 | 0.00113 | GAB4 | GRB2-associated binding protein family, member 4 | TM |
| 277 | chr11 | 76570916 | 76572916 | 12.32268 | 0.001131 | ACER3 | alkaline ceramidase 3 | TM |

| | | | | | | | | |
|---|---|---|---|---|---|---|---|---|
| 278 | chr22 | 39794758 | 39796758 | 17.0131 | 0.001141 | TAB1 | TGF-beta activated kinase 1/MAP3K7 binding protein 1 | TM |
| 279 | chr17 | 73936119 | 73938119 | 12.48031 | 0.001185 | FBF1 | Fas (TNFRSF6) binding factor 1 | TM |
| 280 | chr19 | 12404714 | 12406714 | 21.20105 | 0.001205 | ZNF44 | zinc finger protein 44 | TM |
| 281 | chr15 | 90727151 | 90729151 | 14.38911 | 0.001207 | SEMA4B | sema domain, immunoglobulin domain (Ig), transmembrane domain (TM) and short cytoplasmic domain, (semaphorin) 4B | TM |
| 282 | chr19 | 49954115 | 49956115 | -20.1308 | 0.001207 | PIH1D1 | PIH1 domain containing 1 | TA |
| 283 | chr20 | 61146659 | 61148659 | 10.48776 | 0.001213 | MIR1-1HG | MIR1-1 Host Gene | TM |
| 284 | chr19 | 49989864 | 49991864 | -11.2688 | 0.001228 | RPL13A | ribosomal protein L13a | TA |
| 285 | chr17 | 33813758 | 33815758 | -17.454 | 0.001229 | SLFN12L | schlafen family member 12-like | TA |
| 286 | chr20 | 42142075 | 42144075 | -10.3181 | 0.001229 | L3MBTL1 | l(3)mbt-like 1 (Drosophila) | TA |
| 287 | chr1 | 27560006 | 27562006 | 13.39718 | 0.001235 | WDTC1 | WD and tetratricopeptide repeats 1 | TM |
| 288 | chr11 | 63996752 | 63998752 | -18.4865 | 0.001259 | DNAJC4 | DnaJ (Hsp40) homolog, subfamily C, member 4 | TA |
| 289 | chr1 | 40203516 | 40205516 | 13.61707 | 0.001307 | PPIE | peptidylprolyl isomerase E (cyclophilin E) | TM |
| 290 | chr5 | 176783843 | 176785843 | 11.14789 | 0.001307 | RGS14 | regulator of G-protein signaling 14 | TM |
| 291 | chr10 | 103602677 | 103604677 | -12.926 | 0.001307 | KCNIP2 | Kv channel interacting protein 2 | TA |
| 292 | chr1 | 27708823 | 27710823 | -11.6899 | 0.001338 | CD164L2 | CD164 sialomucin-like 2 | TA |
| 293 | chr17 | 7337761 | 7339761 | 13.90491 | 0.001373 | TMEM102 | transmembrane protein 102 | TM |
| 294 | chr19 | 36000391 | 36002391 | -14.0408 | 0.001373 | DMKN | dermokine | TA |
| 295 | chr1 | 231154670 | 231156670 | 14.40972 | 0.00138 | MIR1182 | microRNA 1182 | TM |
| 296 | chr6 | 28863306 | 28865306 | 14.25183 | 0.001475 | HCG14 | HLA complex group 14 (non-protein coding) | TM |
| 297 | chr6 | 43483728 | 43485728 | 10.54313 | 0.001475 | POLR1C | polymerase (RNA) I polypeptide C, 30kDa | TM |
| 298 | chr17 | 42274529 | 42276529 | 13.65551 | 0.001485 | ATXN7L3 | ataxin 7-like 3 | TM |

| | | | | | | | | |
|---|---|---|---|---|---|---|---|---|
| 299 | chr19 | 36000412 | 36002412 | -14.0217 | 0.001511 | DMKN | dermokine | TA |
| 300 | chr6 | 41702997 | 41704997 | 10.38316 | 0.001522 | TFEB | transcription factor EB | TM |
| 301 | chr1 | 1355824 | 1357824 | -11.8637 | 0.001553 | ANKRD65 | ankyrin repeat domain 65 | TA |
| 302 | chr12 | 53342650 | 53344650 | 11.90578 | 0.001624 | KRT8 | keratin 8 | TM |
| 303 | chr11 | 70934842 | 70936842 | 16.65019 | 0.001657 | SHANK2 | SH3 and multiple ankyrin repeat domains 2 | TM |
| 304 | chr19 | 51225604 | 51227604 | -24.7239 | 0.001686 | CLEC11A | C-type lectin domain family 11, member A | TA |
| 305 | chr6 | 134209258 | 134211258 | -14.2044 | 0.0018 | TCF21 | transcription factor 21 | TA |
| 306 | chr6 | 134209259 | 134211259 | -14.2044 | 0.0018 | TCF21 | transcription factor 21 | TA |
| 307 | chr20 | 3139532 | 3141532 | -16.682 | 0.001809 | UBOX5 | U-box domain containing 5 | TA |
| 308 | chr19 | 10696991 | 10698991 | 17.4369 | 0.001825 | AP1M2 | adaptor-related protein complex 1, mu 2 subunit | TM |
| 309 | chr4 | 698572 | 700572 | 12.46448 | 0.001856 | PCGF3 | polycomb group ring finger 3 | TM |
| 310 | chr15 | 67457492 | 67459492 | 10.24798 | 0.00186 | SMAD3 | SMAD family member 3 | TM |
| 311 | chr19 | 7693439 | 7695439 | -18.5554 | 0.001925 | XAB2 | XPA binding protein 2 | TA |
| 312 | chr12 | 54426733 | 54428733 | 11.95233 | 0.002017 | MIR615 | microRNA 615 | TM |
| 313 | chr17 | 39295739 | 39297739 | 11.09814 | 0.002071 | KRTAP4-6 | keratin associated protein 4-6 | TM |
| 314 | chr5 | 60239905 | 60241905 | -12.4476 | 0.002117 | ERCC8 | excision repair cross-complementing rodent repair deficiency, complementation group 8 | TA |
| 315 | chr6 | 43970387 | 43972387 | -11.4486 | 0.002163 | LOC101929705 | Uncharacterized lncRNA LOC101929705 | TA |
| 316 | chr5 | 175664369 | 175666369 | -12.6952 | 0.002201 | SIMC1 | SUMO-interacting motifs containing 1 | TA |
| 317 | chr1 | 53703281 | 53705281 | -11.7801 | 0.002305 | MAGOH | mago-nashi homolog, proliferation-associated (Drosophila) | TA |
| 318 | chr1 | 53703282 | 53705282 | -11.7801 | 0.002305 | MAGOH | mago-nashi homolog, | TA |

| | | | | | | | | |
|---|---|---|---|---|---|---|---|---|
| | | | | | | | proliferation-associated (Drosophila) | |
| 319 | chr1 | 202136178 | 202138178 | -26.7227 | 0.00233 | PTPRVP | protein tyrosine phosphatase, receptor type, V, pseudogene | TA |
| 320 | chr2 | 3641421 | 3643421 | 14.12858 | 0.002333 | COLEC11 | collectin sub-family member 11 | TM |
| 321 | chr7 | 73497106 | 73499106 | 19.58291 | 0.002365 | LIMK1 | LIM domain kinase 1 | TM |
| 322 | chr17 | 75445612 | 75447612 | 12.63949 | 0.002365 | 9-Sep | septin 9 | TM |
| 323 | chr2 | 175189754 | 175191754 | 18.15453 | 0.002394 | AC018470.4 | long intergenic non-protein coding RNA 1305 | TM |
| 324 | chr6 | 134209144 | 134211144 | -14.5438 | 0.002412 | TCF21 | transcription factor 21 | TA |
| 325 | chr12 | 114845247 | 114847247 | 12.61802 | 0.002412 | TBX5-AS1 | TBX5 antisense RNA 1 | TM |
| 326 | chr11 | 63992341 | 63994341 | -11.9831 | 0.002422 | NUDT22 | nudix (nucleoside diphosphate linked moiety X)-type motif 22 | TA |
| 327 | chr5 | 60239955 | 60241955 | -12.1293 | 0.002461 | ERCC8 | excision repair cross-complementing rodent repair deficiency, complementation group 8 | TA |
| 328 | chr20 | 62583736 | 62585736 | 12.93398 | 0.002577 | UCKL1-AS1 | Uridine-Cytidine Kinase 1-Like 1 Opposite Strand | TM |
| 329 | chr17 | 56768416 | 56770416 | -12.132 | 0.002578 | RAD51C | RAD51 paralog C | TA |
| 330 | chr6 | 82956471 | 82958471 | -10.8012 | 0.002601 | IBTK | inhibitor of Bruton agammaglobulinemia tyrosine kinase | TA |
| 331 | chr7 | 128501856 | 128503856 | 11.53754 | 0.002601 | ATP6V1F | ATPase, H+ transporting, lysosomal 14kDa, V1 subunit F | TM |
| 332 | chr12 | 6755580 | 6757580 | 10.31926 | 0.002688 | ACRBP | acrosin binding protein | TM |
| 333 | chr1 | 11917992 | 11919992 | 15.58047 | 0.002737 | NPPB | natriuretic peptide B | TM |
| 334 | chr17 | 3818960 | 3820960 | -11.2617 | 0.002753 | P2RX1 | purinergic receptor P2X, ligand-gated ion channel, 1 | TA |
| 335 | chr21 | 41238346 | 41240346 | 25.13228 | 0.002767 | PCP4 | Purkinje cell protein 4 | TM |
| 336 | chr11 | 75110434 | 75112434 | 13.1239 | 0.002795 | RPS3 | ribosomal protein S3 | TM |

| | | | | | | | | |
|---|---|---|---|---|---|---|---|---|
| 337 | chr19 | 8407146 | 8409146 | 14.46009 | 0.002798 | KANK3 | KN motif and ankyrin repeat domains 3 | TM |
| 338 | chr19 | 49557997 | 49559997 | 11.25661 | 0.002827 | CGB7 | chorionic gonadotropin, beta polypeptide 7 | TM |
| 339 | chr3 | 10804877 | 10806877 | 14.70725 | 0.002857 | LINC00606 | long intergenic non-protein coding RNA 606 | TM |
| 340 | chr10 | 114134955 | 114136955 | -16.8043 | 0.002871 | ACSL5 | acyl-CoA synthetase long-chain family member 5 | TA |
| 341 | chr1 | 35543963 | 35545963 | 14.52109 | 0.002895 | ZMYM1 | zinc finger, MYM-type 1 | TM |
| 342 | chr13 | 97635426 | 97637426 | -24.506 | 0.002947 | LINC00359 | long intergenic non-protein coding RNA 359 | TA |
| 343 | chr17 | 47571654 | 47573654 | -10.6905 | 0.002967 | NGFR | nerve growth factor receptor | TA |
| 344 | chr14 | 73392039 | 73394039 | -11.4017 | 0.003029 | DCAF4 | DDB1 and CUL4 associated factor 4 | TA |
| 345 | chr4 | 1302578 | 1304578 | 11.85714 | 0.003061 | MAEA | macrophage erythroblast attacher | TM |
| 346 | chr13 | 110052042 | 110054042 | -30.4521 | 0.003084 | LINC00399 | long intergenic non-protein coding RNA 399 | TA |
| 347 | chr7 | 154001296 | 154003296 | -13.3974 | 0.003137 | DPP6 | dipeptidyl-peptidase 6 | TA |
| 348 | chr16 | 30995518 | 30997518 | 14.91577 | 0.003164 | HSD3B7 | hydroxy-delta-5-steroid dehydrogenase, 3 beta- and steroid delta-isomerase 7 | TM |
| 349 | chr10 | 71903484 | 71905484 | 11.10034 | 0.003167 | TYSND1 | trypsin domain containing 1 | TM |
| 350 | chr15 | 40632168 | 40634168 | 35.86635 | 0.003262 | C15orf52 | chromosome 15 open reading frame 52 | TM |
| 351 | chr13 | 100150560 | 100152560 | 14.52733 | 0.003291 | LINC01232 | Long Intergenic Non-Protein Coding RNA 1232 | TM |
| 352 | chr17 | 62776622 | 62778622 | -10.3273 | 0.003292 | hsa-mir-6080 | hsa-mir-6080 | TA |
| 353 | chr9 | 97020547 | 97022547 | 14.47194 | 0.003389 | ZNF169 | zinc finger protein 169 | TM |
| 354 | chr15 | 25469349 | 25471349 | 17.44365 | 0.003454 | SNORD115-30 | small nucleolar RNA, C/D box 115-30 | TM |
| 355 | chr19 | 36427021 | 36429021 | 12.27521 | 0.003539 | LRFN3 | leucine rich repeat and fibronectin type | TM |

| | | | | | | | | |
|---|---|---|---|---|---|---|---|---|
| | | | | | | | III domain containing 3 | |
| 356 | chr20 | 62581527 | 62583527 | -11.8067 | 0.003539 | UCKL1 | uridine-cytidine kinase 1-like 1 | TA |
| 357 | chr7 | 56018610 | 56020610 | -15.8956 | 0.003656 | MRPS17 | 28S ribosomal protein S17, mitochondrial; HCG1984214, isoform CRA_a | TA |
| 358 | chr7 | 143598172 | 143600172 | 19.41143 | 0.003656 | FAM115A | family with sequence similarity 115, member A | TM |
| 359 | chr7 | 143598278 | 143600278 | 19.41143 | 0.003656 | FAM115A | family with sequence similarity 115, member A | TM |
| 360 | chr16 | 8961869 | 8963869 | 11.42266 | 0.003735 | CARHSP1 | calcium regulated heat stable protein 1, 24kDa | TM |
| 361 | chr14 | 24562339 | 24564339 | 18.05556 | 0.003814 | PCK2 | phosphoenolpyruvate carboxykinase 2 (mitochondrial) | TM |
| 362 | chr12 | 52714182 | 52716182 | -17.007 | 0.003843 | KRT83 | keratin 83 | TA |
| 363 | chr10 | 134778037 | 134780037 | -11.3927 | 0.003864 | LINC01166 | long intergenic non-protein coding RNA 1166 | TA |
| 364 | chr20 | 60173816 | 60175816 | 10.00651 | 0.003886 | CDH4 | cadherin 4, type 1, R-cadherin (retinal) | TM |
| 365 | chr12 | 185541 | 187541 | -11.706 | 0.004034 | IQSEC3 | IQ motif and Sec7 domain 3 | TA |
| 366 | chr16 | 56690854 | 56692854 | 17.07041 | 0.004035 | MT1F | metallothionein 1F | TM |
| 367 | chr17 | 18758611 | 18760611 | -21.9978 | 0.004163 | PRPSAP2 | phosphoribosyl pyrophosphate synthetase-associated protein 2 | TA |
| 368 | chr17 | 75282972 | 75284972 | -13.5262 | 0.004204 | 9-Sep | septin 9 | TA |
| 369 | chr9 | 130692076 | 130694076 | 13.75349 | 0.004287 | PIP5KL1 | phosphatidylinositol-4-phosphate 5-kinase-like 1 | TM |
| 370 | chr11 | 113257512 | 113259512 | 10.87553 | 0.004338 | ANKK1 | ankyrin repeat and kinase domain containing 1 | TM |
| 371 | chr11 | 3646689 | 3648689 | -10.9217 | 0.004353 | TRPC2 | transient receptor potential cation channel, subfamily C, member 2, pseudogene | TA |
| 372 | chr2 | 120435742 | 120437742 | 10.78137 | 0.004354 | TMEM177 | transmembrane protein 177 | TM |
| 373 | chr12 | 56861300 | 56863300 | 15.09637 | 0.004368 | SPRYD4 | SPRY domain containing 4 | TM |

| | | | | | | | | |
|---|---|---|---|---|---|---|---|---|
| 374 | chr8 | 22088851 | 22090851 | -18.6134 | 0.004371 | PHYHIP | phytanoyl-CoA 2-hydroxylase interacting protein | TA |
| 375 | chr11 | 35158579 | 35160579 | -23.9574 | 0.004372 | CD44 | CD44 molecule (Indian blood group) | TA |
| 376 | chr20 | 25387322 | 25389322 | -16.2449 | 0.004473 | GINS1 | GINS complex subunit 1 (Psf1 homolog) | TA |
| 377 | chr6 | 43275530 | 43277530 | 20.88509 | 0.004549 | CRIP3 | cysteine-rich protein 3 | TM |
| 378 | chr15 | 34628045 | 34630045 | -19.505 | 0.004601 | SLC12A6 | solute carrier family 12 (potassium/chloride transporter), member 6 | TA |
| 379 | chr20 | 23585610 | 23587610 | -21.9986 | 0.004623 | CST9 | cystatin 9 (testatin) | TA |
| 380 | chr19 | 49928687 | 49930687 | -26.1588 | 0.004698 | GFY | golgi-associated, olfactory signaling regulator | TA |
| 381 | chr9 | 116839752 | 116841752 | -20.2306 | 0.004791 | AMBP | alpha-1-microglobulin/bikunin precursor | TA |
| 382 | chr20 | 48908256 | 48910256 | 12.71843 | 0.004817 | RP11-290F20.1 | Uncharacterized LOC284751 | TM |
| 383 | chr16 | 30621096 | 30623096 | 10.64867 | 0.004901 | ZNF689 | zinc finger protein 689 | TM |
| 384 | chr8 | 67089846 | 67091846 | 10.87427 | 0.00493 | CRH | corticotropin releasing hormone | TM |
| 385 | chr8 | 133686863 | 133688863 | -16.1293 | 0.004994 | LRRC6 | leucine rich repeat containing 6 | TA |
| 386 | chr1 | 2283100 | 2285100 | -12.1487 | 0.005083 | RP4-740C4.6 | DNA sequence from clone RP4-740C4.67 on chromosome 1 | TA |
| 387 | chr1 | 43734664 | 43736664 | -20.3902 | 0.005188 | TMEM125 | transmembrane protein 125 | TA |
| 388 | chr16 | 30022669 | 30024669 | 10.76773 | 0.005286 | DOC2A | double C2-like domains, alpha | TM |
| 389 | chr19 | 2281181 | 2283181 | 14.59343 | 0.005531 | C19orf35 | chromosome 19 open reading frame 35 | TM |
| 390 | chr11 | 67382135 | 67384135 | -14.3312 | 0.005535 | DOC2GP | double C2-like domains, gamma, pseudogene | TA |
| 391 | chr12 | 53011343 | 53013343 | 15.59735 | 0.005647 | KRT73 | keratin 73 | TM |
| 392 | chrX | 47478256 | 47480256 | -11.5391 | 0.005647 | SYN1 | synapsin I | TA |
| 393 | chr7 | 73255855 | 73257855 | 14.87455 | 0.00567 | WBSCR27 | Williams Beuren syndrome | TM |

| | | | | | | | | |
|---|---|---|---|---|---|---|---|---|
| | | | | | | | chromosome region 27 | |
| 394 | chr9 | 35859270 | 35861270 | 20.63095 | 0.005701 | LINC00950 | Long Intergenic Non-Protein Coding RNA 950 | TM |
| 395 | chr16 | 77755388 | 77757388 | -13.0214 | 0.005825 | NUDT7 | nudix (nucleoside diphosphate linked moiety X)-type motif 7 | TA |
| 396 | chr10 | 121136483 | 121138483 | -12.6676 | 0.006033 | MIR4681 | microRNA 4681 | TA |
| 397 | chr12 | 32831133 | 32833133 | 11.23387 | 0.006154 | DNM1L | dynamin 1-like | TM |
| 398 | chr4 | 142252771 | 142254771 | -15.7284 | 0.006221 | LINC02432 | Long Intergenic Non-Protein Coding RNA 2432 | TA |
| 399 | chr11 | 67170446 | 67172446 | 18.43995 | 0.006221 | TBC1D10C | TBC1 domain family, member 10C | TM |
| 400 | chr20 | 32307136 | 32309136 | -10.516 | 0.006576 | PXMP4 | peroxisomal membrane protein 4, 24kDa | TA |
| 401 | chr1 | 155289639 | 155291639 | -18.9879 | 0.006821 | RUSC1 | RUN and SH3 domain containing 1 | TA |
| 402 | chr17 | 38443145 | 38445145 | 10.73009 | 0.006821 | CDC6 | cell division cycle 6 | TM |
| 403 | chr1 | 20616411 | 20618411 | -22.6006 | 0.006889 | VWA5B1 | von Willebrand factor A domain containing 5B1 | TA |
| 404 | chr9 | 116326233 | 116328233 | 14.64135 | 0.006898 | RGS3 | regulator of G-protein signaling 3 | TM |
| 405 | chr16 | 15743082 | 15745082 | -10.8377 | 0.006969 | NDE1 | nudE neurodevelopment protein 1 | TA |
| 406 | chr17 | 4462891 | 4464891 | -12.062 | 0.007095 | GGT6 | gamma-glutamyltransferase 6 | TA |
| 407 | chr19 | 49140295 | 49142295 | -18.8843 | 0.007095 | DBP | D site of albumin promoter (albumin D-box) binding protein | TA |
| 408 | chr11 | 118900616 | 118902616 | 10.36137 | 0.00719 | SLC37A4 | solute carrier family 37 (glucose-6-phosphate transporter), member 4 | TM |
| 409 | chr4 | 123357 | 125357 | -10.2693 | 0.007205 | ZNF718 | zinc finger protein 718 | TA |
| 410 | chr6 | 159184785 | 159186785 | -10.1498 | 0.007248 | MIR3918 | microRNA 3918 | TA |
| 411 | chr1 | 227750219 | 227752219 | 15.74628 | 0.007251 | ZNF678 | zinc finger protein 678 | TM |
| 412 | chr1 | 161574848 | 161576848 | -11.4265 | 0.007315 | HSPA7 | heat shock 70kDa protein 7 (HSP70B) | TA |

| | | | | | | | | |
|---|---|---|---|---|---|---|---|---|
| 413 | chr6 | 34481648 | 34483648 | 13.28454 | 0.007407 | PACSIN1 | protein kinase C and casein kinase substrate in neurons 1 | TM |
| 414 | chr20 | 25012342 | 25014342 | 16.32035 | 0.007477 | ACSS1 | acyl-CoA synthetase short-chain family member 1 | TM |
| 415 | chr9 | 135229372 | 135231372 | -11.8024 | 0.007588 | SETX | senataxin | TA |
| 416 | chr2 | 96990934 | 96992934 | -12.7256 | 0.007602 | ITPRIPL1 | inositol 1,4,5-trisphosphate receptor interacting protein-like 1 | TA |
| 417 | chr2 | 96990944 | 96992944 | -12.7256 | 0.007602 | ITPRIPL1 | inositol 1,4,5-trisphosphate receptor interacting protein-like 1 | TA |
| 418 | chr17 | 38217445 | 38219445 | 13.74375 | 0.007664 | THRA | thyroid hormone receptor, alpha | TM |
| 419 | chr1 | 213187851 | 213189851 | -10.2297 | 0.007685 | ANGEL2 | angel homolog 2 (Drosophila) | TA |
| 420 | chr1 | 213188217 | 213190217 | -10.2297 | 0.007685 | ANGEL2 | angel homolog 2 (Drosophila) | TA |
| 421 | chr1 | 2487450 | 2489450 | 14.52923 | 0.00779 | RP3-395M20.8 | Uncharacterized lncRNA LOC100996583 | TM |
| 422 | chr6 | 29941891 | 29943891 | 16.00103 | 0.007828 | HCG9 | HLA complex group 9 (non-protein coding) | TM |
| 423 | chr17 | 42991920 | 42993920 | -11.3621 | 0.007858 | GFAP | glial fibrillary acidic protein | TA |
| 424 | chr19 | 11545465 | 11547465 | -17.9402 | 0.007933 | PRKCSH | protein kinase C substrate 80K-H | TA |
| 425 | chr22 | 20136431 | 20138431 | 10.77494 | 0.008253 | CCDC188 | Coiled-Coil Domain Containing 188 | TM |
| 426 | chr20 | 44485048 | 44487048 | 10.13341 | 0.008282 | ZSWIM3 | zinc finger, SWIM-type containing 3 | TM |
| 427 | chr6 | 31773761 | 31775761 | -11.7983 | 0.008304 | LSM2 | LSM2 homolog, U6 small nuclear RNA associated (S. cerevisiae) | TA |
| 428 | chr7 | 142959521 | 142961521 | -15.2584 | 0.00831 | GSTK1 | glutathione S-transferase kappa 1 | TA |
| 429 | chr4 | 15963859 | 15965859 | -17.3064 | 0.008392 | FGFBP2 | fibroblast growth factor binding protein 2 | TA |
| 430 | chr4 | 141418531 | 141420531 | 15.3645 | 0.008433 | RP11-542P2.1 | Glycosyltransferase 54 domain-containing protein | TM |

| | | | | | | | | |
|---|---|---|---|---|---|---|---|---|
| 431 | chr1 | 202935404 | 202937404 | -10.7196 | 0.008616 | CYB5R1 | cytochrome b5 reductase 1 | TA |
| 432 | chr11 | 116657739 | 116659739 | -20.501 | 0.008825 | ZNF259 | zinc finger protein 259 | TA |
| 433 | chr17 | 34078897 | 34080897 | 17.50334 | 0.009176 | GAS2L2 | growth arrest-specific 2 like 2 | TM |
| 434 | chr11 | 298526 | 300526 | 11.40773 | 0.009271 | IFITM5 | interferon induced transmembrane protein 5 | TM |
| 435 | chr14 | 101520594 | 101522594 | 17.4333 | 0.009417 | MIR323B | microRNA 323b | TM |
| 436 | chr5 | 138724605 | 138726605 | 14.0925 | 0.009581 | MZB1 | marginal zone B and B1 cell-specific protein | TM |
| 437 | chr7 | 150651917 | 150653917 | 11.70187 | 0.009683 | KCNH2 | potassium voltage-gated channel, subfamily H (eag-related), member 2 | TM |
| 438 | chrX | 153045455 | 153047455 | 13.3666 | 0.010347 | SRPK3 | SRSF protein kinase 3 | TM |
| 439 | chr22 | 29976085 | 29978085 | -12.7895 | 0.010357 | NIPSNAP1 | nipsnap homolog 1 (C. elegans) | TA |
| 440 | chr22 | 29976326 | 29978326 | -12.7895 | 0.010357 | NIPSNAP1 | nipsnap homolog 1 (C. elegans) | TA |
| 441 | chr1 | 228351549 | 228353549 | 12.55147 | 0.010725 | IBA57 | IBA57, iron-sulfur cluster assembly homolog (S. cerevisiae) | TM |
| 442 | chr4 | 103939896 | 103941896 | 10.60606 | 0.010725 | SLC9B1 | solute carrier family 9, subfamily B (NHA1, cation proton antiporter 1), member 1 | TM |
| 443 | chr13 | 111521655 | 111523655 | -14.3305 | 0.010729 | LINC00346 | long intergenic non-protein coding RNA 346 | TA |
| 444 | chr14 | 91709852 | 91711852 | -20.6573 | 0.010949 | GPR68 | G protein-coupled receptor 68 | TA |
| 445 | chr3 | 149955486 | 149957486 | -13.9535 | 0.011095 | RP11-167H9.6 | lncRNA RP11-167H9.6 | TA |
| 446 | chr2 | 241563661 | 241565661 | -10.2644 | 0.011169 | GPR35 | G protein-coupled receptor 35 | TA |
| 447 | chr3 | 9943295 | 9945295 | 22.87611 | 0.01117 | IL17RE | interleukin 17 receptor E | TM |
| 448 | chr2 | 239139318 | 239141318 | -11.3793 | 0.011262 | LOC643387 | TAR DNA Binding Protein Pseudogene | TA |
| 449 | chr2 | 239139326 | 239141326 | -11.3793 | 0.011262 | LOC643387 | TAR DNA Binding Protein Pseudogene | TA |
| 450 | chr15 | 21939739 | 21941739 | -21.4194 | 0.011262 | RP11-32B5.1 | LincRNA RP11-32B5.1 | TA |

| | | | | | | | | |
|---|---|---|---|---|---|---|---|---|
| 451 | chr19 | 7693670 | 7695670 | -15.8337 | 0.011546 | XAB2 | XPA binding protein 2 | TA |
| 452 | chr5 | 10307168 | 10309168 | 10.29807 | 0.011637 | CMBL | carboxymethylenebutenolidase homolog (Pseudomonas) | TM |
| 453 | chr5 | 270639 | 272639 | 13.54642 | 0.011904 | PDCD6 | programmed cell death 6 | TM |
| 454 | chr12 | 50296760 | 50298760 | 15.4851 | 0.011908 | FAIM2 | Fas apoptotic inhibitory molecule 2 | TM |
| 455 | chr11 | 17564963 | 17566963 | -15.0957 | 0.011965 | USH1C | Usher syndrome 1C (autosomal recessive, severe) | TA |
| 456 | chr8 | 10382055 | 10384055 | 18.13581 | 0.012218 | PRSS55 | protease, serine, 55 | TM |
| 457 | chr3 | 183992798 | 183994798 | -10.8052 | 0.01231 | ECE2 | endothelin converting enzyme 2 | TA |
| 458 | chr10 | 103985034 | 103987034 | -13.1778 | 0.01296 | ELOVL3 | ELOVL fatty acid elongase 3 | TA |
| 459 | chr12 | 111357404 | 111359404 | 11.13942 | 0.013008 | MYL2 | myosin, light chain 2, regulatory, cardiac, slow | TM |
| 460 | chr2 | 26623783 | 26625783 | 10.48454 | 0.013216 | DRC1 | dynein regulatory complex subunit 1 homolog (Chlamydomonas) | TM |
| 461 | chr11 | 124542739 | 124544739 | 14.31302 | 0.013288 | SPA17 | sperm autoantigenic protein 17 | TM |
| 462 | chr11 | 124542777 | 124544777 | 14.31302 | 0.013288 | SPA17 | sperm autoantigenic protein 17 | TM |
| 463 | chr20 | 61667380 | 61669380 | 14.29952 | 0.0134 | LINC00029 | long intergenic non-protein coding RNA 29 | TM |
| 464 | chr17 | 27223715 | 27225715 | -10.1928 | 0.013499 | FLOT2 | flotillin 2 | TA |
| 465 | chr13 | 103718196 | 103720196 | 19.11392 | 0.013537 | SLC10A2 | solute carrier family 10 (sodium/bile acid cotransporter), member 2 | TM |
| 466 | chr17 | 43509282 | 43511282 | -13.7495 | 0.013788 | ARHGAP27 | Rho GTPase activating protein 27 | TA |
| 467 | chr2 | 242822513 | 242824513 | 18.09524 | 0.013988 | AC131097.3 | Lnc-RTP5-10 | TM |
| 468 | chr17 | 4872066 | 4874066 | -12.2891 | 0.014215 | MIR6864 | MicroRNA 6864 | TA |
| 469 | chr16 | 70322412 | 70324412 | -10.4788 | 0.014544 | DDX19B | DEAD (Asp-Glu-Ala-Asp) box polypeptide 19B | TA |
| 470 | chr5 | 159625047 | 159627047 | 14.57521 | 0.015176 | FABP6 | fatty acid binding protein 6, ileal | TM |

| | | | | | | | | |
|---|---|---|---|---|---|---|---|---|
| 471 | chr19 | 47735023 | 47737023 | -18.2591 | 0.015511 | BBC3 | BCL2 binding component 3 | TA |
| 472 | chr19 | 52429687 | 52431687 | 12.80945 | 0.015873 | ZNF613 | zinc finger protein 613 | TM |
| 473 | chr5 | 140200360 | 140202360 | -18.6032 | 0.015932 | PCDHA5 | protocadherin alpha 5 | TA |
| 474 | chr2 | 152145430 | 152147430 | 11.6639 | 0.015936 | NMI | N-myc (and STAT) interactor | TM |
| 475 | chr16 | 30750511 | 30752511 | -10.8739 | 0.016061 | TMEM265 | Transmembrane Protein 265 | TA |
| 476 | chr16 | 19534178 | 19536178 | 13.90348 | 0.016605 | CCP110 | centriolar coiled coil protein 110kDa | TM |
| 477 | chr17 | 7831753 | 7833753 | 10.49175 | 0.016605 | KCNAB3 | potassium voltage-gated channel, shaker-related subfamily, beta member 3 | TM |
| 478 | chr19 | 58280019 | 58282019 | 14.64212 | 0.016742 | ZNF586 | zinc finger protein 586 | TM |
| 479 | chr2 | 106681112 | 106683112 | 10.67008 | 0.016743 | C2orf40 | chromosome 2 open reading frame 40 | TM |
| 480 | chr10 | 116443414 | 116445414 | -26.2642 | 0.016743 | ABLIM1 | actin binding LIM protein 1 | TA |
| 481 | chr2 | 237415178 | 237417178 | -15.9873 | 0.016766 | IQCA1 | IQ motif containing with AAA domain 1 | TA |
| 482 | chrX | 139172825 | 139174825 | -12.4628 | 0.017057 | LOC389895 | Uncharacterized Protein LOC389895 | TA |
| 483 | chr7 | 99062824 | 99064824 | 13.03034 | 0.01707 | ATP5J2 | ATP synthase, H+ transporting, mitochondrial Fo complex, subunit F2 | TM |
| 484 | chr3 | 9943511 | 9945511 | 22.55102 | 0.017102 | IL17RE | interleukin 17 receptor E | TM |
| 485 | chr8 | 144765621 | 144767621 | -10.5052 | 0.017776 | ZNF707 | zinc finger protein 707 | TA |
| 486 | chr8 | 55046780 | 55048780 | 11.08811 | 0.017999 | MRPL15 | mitochondrial ribosomal protein L15 | TM |
| 487 | chr8 | 143822829 | 143824829 | -10.3043 | 0.017999 | SLURP1 | secreted LY6/PLAUR domain containing 1 | TA |
| 488 | chr1 | 24740587 | 24742587 | -13.0601 | 0.018235 | STPG1 | sperm-tail PG-rich repeat containing 1 | TA |
| 489 | chr10 | 98861512 | 98863512 | 24.11235 | 0.018293 | RP11-453E2.2 | Uncharacterized antisense RNA LOC100505540 | TM |
| 490 | chr17 | 38519945 | 38521945 | 10.64665 | 0.018354 | GJD3 | gap junction protein, delta 3, 31.9kDa | TM |

| | | | | | | | | |
|---|---|---|---|---|---|---|---|---|
| 491 | chr9 | 137297427 | 137299427 | 12.61173 | 0.019303 | RXRA | retinoid X receptor, alpha | TM |
| 492 | chr22 | 37607416 | 37609416 | 13.69205 | 0.019303 | SSTR3 | somatostatin receptor 3 | TM |
| 493 | chr7 | 73274488 | 73276488 | 14.13858 | 0.019728 | WBSCR28 | Williams-Beuren syndrome chromosome region 28 | TM |
| 494 | chr10 | 106033886 | 106035886 | 10.83864 | 0.019774 | GSTO2 | glutathione S-transferase omega 2 | TM |
| 495 | chr10 | 71175674 | 71177674 | -13.018 | 0.019903 | TACR2 | tachykinin receptor 2 | TA |
| 496 | chr8 | 145700718 | 145702718 | -10.9324 | 0.020524 | FOXH1 | forkhead box H1 | TA |
| 497 | chr9 | 130880013 | 130882013 | -14.4271 | 0.020663 | RP11-395P17.3 | Uncharacterized LOC100289019 | TA |
| 498 | chr21 | 43482067 | 43484067 | -13.5714 | 0.020903 | UMODL1 | uromodulin-like 1 | TA |
| 499 | chr13 | 100057554 | 100059554 | 15.8803 | 0.020905 | MIR548AN | microRNA 548an | TM |
| 500 | chr2 | 128292377 | 128294377 | -12.8972 | 0.021389 | MYO7B | myosin VIIB | TA |
| 501 | chr14 | 31027328 | 31029328 | 12.0582 | 0.021407 | G2E3 | G2/M-phase specific E3 ubiquitin protein ligase | TM |
| 502 | chr16 | 2835708 | 2837708 | -11.2196 | 0.021509 | PRSS33 | protease, serine, 33 | TA |
| 503 | chr16 | 70206351 | 70208351 | 13.25758 | 0.022178 | LOC400541 | Uncharacterized LOC400541 | TM |
| 504 | chr16 | 70206927 | 70208927 | 13.25758 | 0.022178 | LOC400541 | Uncharacterized LOC400541 | TM |
| 505 | chr6 | 42017250 | 42019250 | 10.96999 | 0.02252 | TAF8 | TAF8 RNA polymerase II, TATA box binding protein (TBP)-associated factor, 43kDa | TM |
| 506 | chr1 | 24017268 | 24019268 | -18.8214 | 0.022786 | RPL11 | ribosomal protein L11 | TA |
| 507 | chr6 | 26224382 | 26226382 | 12.35772 | 0.023018 | HIST1H3E | histone cluster 1, H3e | TM |
| 508 | chr3 | 10435246 | 10437246 | -11.9396 | 0.023923 | MIR885 | microRNA 885 | TA |
| 509 | chr20 | 30134184 | 30136184 | 11.81962 | 0.023923 | RP3-324O17.4 | DNA sequence from clone RP3-324O17 on chromosome 20 | TM |
| 510 | chr12 | 110210292 | 110212292 | -22.5954 | 0.024117 | FAM222A-AS1 | FAM222A antisense RNA 1 | TA |
| 511 | chr1 | 160231277 | 160233277 | 12.2113 | 0.024882 | DCAF8 | DDB1 and CUL4 associated factor 8 | TM |
| 512 | chr1 | 160231318 | 160233318 | 12.2113 | 0.024882 | DCAF8 | DDB1- and CUL4-associated factor 8 | TM |

| | | | | | | | | |
|---|---|---|---|---|---|---|---|---|
| 513 | chr1 | 160231350 | 160233350 | 12.2113 | 0.024882 | DCAF8 | DDB1- and CUL4-associated factor 8 | TM |
| 514 | chr12 | 58002962 | 58004962 | -18.985 | 0.025015 | ARHGEF25 | Rho guanine nucleotide exchange factor (GEF) 25 | TA |
| 515 | chr19 | 4516716 | 4518716 | -10.4404 | 0.025577 | PLIN4 | perilipin 4 | TA |
| 516 | chr18 | 47016717 | 47018717 | 16.76172 | 0.025621 | SNORD58A | small nucleolar RNA, C/D box 58A | TM |
| 517 | chr19 | 4769681 | 4771681 | 26.02496 | 0.025822 | MIR7-3 | microRNA 7-3 | TM |
| 518 | chr1 | 115396423 | 115398423 | 14.5074 | 0.02598 | SYCP1 | synaptonemal complex protein 1 | TM |
| 519 | chr3 | 124302505 | 124304505 | -11.4357 | 0.026425 | KALRN | kalirin, RhoGEF kinase | TA |
| 520 | chr17 | 18011019 | 18013019 | 11.24752 | 0.026434 | MYO15A | myosin XVA | TM |
| 521 | chr19 | 22789817 | 22791817 | 19.42623 | 0.026587 | LOC100996349 | Testis Expressed 264, ER-Phagy Receptor | TM |
| 522 | chr19 | 35835415 | 35837415 | 10.56165 | 0.026728 | MIR5196 | microRNA 5196 | TM |
| 523 | chr1 | 154126592 | 154128592 | -14.658 | 0.027089 | NUP210L | nucleoporin 210kDa-like | TA |
| 524 | chr14 | 101520755 | 101522755 | 13.63332 | 0.027196 | MIR323B | microRNA 323b | TM |
| 525 | chr2 | 162271619 | 162273619 | -13.0461 | 0.027208 | TBR1 | T-box, brain, 1 | TA |
| 526 | chr14 | 23351431 | 23353431 | -25.0196 | 0.027323 | REM2 | RAS (RAD and GEM)-like GTP binding 2 | TA |
| 527 | chr2 | 97172846 | 97174846 | -10.1111 | 0.027418 | NEURL3 | neuralized E3 ubiquitin protein ligase 3 | TA |
| 528 | chr16 | 3492490 | 3494490 | 10.88286 | 0.028267 | ZNF597 | zinc finger protein 597 | TM |
| 529 | chr16 | 3492667 | 3494667 | 10.88286 | 0.028267 | ZNF597 | zinc finger protein 597 | TM |
| 530 | chr1 | 112398527 | 112400527 | -16.3276 | 0.028417 | KCND3-IT1 | KCND3 intronic transcript 1 (non-protein coding) | TA |
| 531 | chr7 | 99690266 | 99692266 | 14.05375 | 0.028417 | MIR93 | microRNA 93 | TM |
| 532 | chr13 | 47470211 | 47472211 | -10.2348 | 0.028528 | HTR2A | 5-hydroxytryptamine (serotonin) receptor 2A, G protein-coupled | TA |
| 533 | chr11 | 133901166 | 133903166 | -16.9268 | 0.029654 | RP11-713P17.3 | Uncharacterized lncRNA LOC100128239 | TA |
| 534 | chr12 | 52280792 | 52282792 | 13.27711 | 0.029654 | ANKRD33 | ankyrin repeat domain 33 | TM |
| 535 | chr1 | 26550810 | 26552810 | -10.2673 | 0.030125 | LOC101928303 | Uncharacterized LOC101928303 pseudogene | TA |

| | | | | | | | | | |
|---|---|---|---|---|---|---|---|---|---|
| 536 | chr15 | 25455838 | 25457838 | -15.2301 | 0.030353 | PWAR4 | Prader Willi/Angelman Region Gene 4 | TA |
| 537 | chr15 | 25455942 | 25457942 | -15.2301 | 0.030353 | PWAR4 | Prader Willi/Angelman Region Gene 4 | TA |
| 538 | chr1 | 120438147 | 120440147 | -12.6512 | 0.031161 | ADAM30 | ADAM metallopeptidase domain 30 | TA |
| 539 | chr14 | 93672459 | 93674459 | -10.9617 | 0.031261 | UBR7 | ubiquitin protein ligase E3 component n-recognin 7 (putative) | TA |
| 540 | chr16 | 56650372 | 56652372 | -13.2511 | 0.031329 | MT1L | metallothionein 1L (gene/pseudogene) | TA |
| 541 | chr16 | 69759533 | 69761533 | 15.32276 | 0.031329 | NQO1 | NAD(P)H dehydrogenase, quinone 1 | TM |
| 542 | chr16 | 69759571 | 69761571 | 15.32276 | 0.031329 | NQO1 | NAD(P)H dehydrogenase, quinone 1 | TM |
| 543 | chr7 | 23220445 | 23222445 | 11.45322 | 0.031601 | NUPL2 | nucleoporin like 2 | TM |
| 544 | chr4 | 25313395 | 25315395 | -12.7752 | 0.032105 | ZCCHC4 | zinc finger, CCHC domain containing 4 | TA |
| 545 | chr1 | 151687792 | 151689792 | 22.17164 | 0.032201 | CELF3 | CUGBP, Elav-like family member 3 | TM |
| 546 | chr5 | 111495222 | 111497222 | 10.8883 | 0.033526 | SNORA13 | small nucleolar RNA, H/ACA box 13 | TM |
| 547 | chr22 | 39708916 | 39710916 | -11.23 | 0.034184 | SNORD83B | small nucleolar RNA, C/D box 83B | TA |
| 548 | chr17 | 56405152 | 56407152 | -10.898 | 0.034647 | BZRAP1-AS1 | BZRAP1 antisense RNA 1 | TA |
| 549 | chr17 | 10706416 | 10708416 | 11.47239 | 0.034795 | LINC00675 | long intergenic non-protein coding RNA 675 | TM |
| 550 | chr14 | 93672400 | 93674400 | -11.2695 | 0.034889 | UBR7 | ubiquitin protein ligase E3 component n-recognin 7 (putative) | TA |
| 551 | chr17 | 56405298 | 56407298 | -11.7383 | 0.035356 | BZRAP1-AS1 | BZRAP1 antisense RNA 1 | TA |
| 552 | chr11 | 117694459 | 117696459 | -15.1296 | 0.03544 | FXYD2 | FXYD domain containing ion transport regulator 2 | TA |
| 553 | chr12 | 123751801 | 123753801 | -11.0233 | 0.035552 | CDK2AP1 | cyclin-dependent kinase 2 associated protein 1 | TA |

| | | | | | | | | |
|---|---|---|---|---|---|---|---|---|
| 554 | chr1 | 248683947 | 248685947 | 11.90989 | 0.03584 | OR2G6 | olfactory receptor, family 2, subfamily G, member 6 | TM |
| 555 | chr11 | 116662136 | 116664136 | 15.30117 | 0.036755 | APOA5 | apolipoprotein A-V | TM |
| 556 | chr10 | 51564107 | 51566107 | -11.3203 | 0.03676 | NCOA4 | nuclear receptor coactivator 4 | TA |
| 557 | chr12 | 54777514 | 54779514 | -15.1393 | 0.036863 | ZNF385A | zinc finger protein 385A | TA |
| 558 | chr6 | 10886063 | 10888063 | -15.2941 | 0.036952 | SYCP2L | synaptonemal complex protein 2-like | TA |
| 559 | chr8 | 143850246 | 143852246 | 11.30636 | 0.038311 | LYNX1 | Ly6/neurotoxin 1 | TM |
| 560 | chr1 | 32280652 | 32282652 | -12.6043 | 0.038458 | SPOCD1 | SPOC domain containing 1 | TA |
| 561 | chr16 | 16461732 | 16463732 | 14.40033 | 0.038661 | MIR6511A2 | MicroRNA 6511a-2 | TM |
| 562 | chr10 | 42969938 | 42971938 | 12.50518 | 0.039583 | LINC00839 | long intergenic non-protein coding RNA 839 | TM |
| 563 | chr12 | 1737411 | 1739411 | -14.1133 | 0.039778 | WNT5B | wingless-type MMTV integration site family, member 5B | TA |
| 564 | chr3 | 157260132 | 157262132 | -10.5148 | 0.039853 | C3orf55 | chromosome 3 open reading frame 55 | TA |
| 565 | chr17 | 41475352 | 41477352 | -12.2316 | 0.039853 | ARL4D | ADP-ribosylation factor-like 4D | TA |
| 566 | chr17 | 8150413 | 8152413 | -17.328 | 0.039888 | CTC1 | CTS telomere maintenance complex component 1 | TA |
| 567 | chr3 | 75678913 | 75680913 | 16.8673 | 0.040121 | MIR1324 | microRNA 1324 | TM |
| 568 | chr12 | 117746215 | 117748215 | 17.85714 | 0.040211 | NOS1 | nitric oxide synthase 1 (neuronal) | TM |
| 569 | chr18 | 595997 | 597997 | -16.463 | 0.040992 | CLUL1 | clusterin-like 1 (retinal) | TA |
| 570 | chr16 | 85338931 | 85340931 | 14.25516 | 0.041762 | MIR5093 | microRNA 5093 | TM |
| 571 | chr19 | 55586220 | 55588220 | -16.5421 | 0.04221 | EPS8L1 | EPS8-like 1 | TA |
| 572 | chr16 | 50299450 | 50301450 | 22.55754 | 0.043474 | ADCY7 | adenylate cyclase 7 | TM |
| 573 | chr5 | 150137657 | 150139657 | 17.75736 | 0.043494 | DCTN4 | dynactin 4 (p62) | TM |
| 574 | chr14 | 72053287 | 72055287 | -15.6464 | 0.045006 | SIPA1L1 | signal-induced proliferation-associated 1 like 1 | TA |
| 575 | chr7 | 99690470 | 99692470 | 13.28321 | 0.045123 | MIR93 | microRNA 93 | TM |
| 576 | chr12 | 116865123 | 116867123 | -13.5337 | 0.046002 | MIR4472-2 | microRNA 4472-2 | TA |
| 577 | chr17 | 46650810 | 46652810 | -14.3978 | 0.046726 | HOXB3 | homeobox B3 | TA |
| 578 | chr2 | 11271302 | 11273302 | -10.1419 | 0.04747 | C2orf50 | chromosome 2 open reading frame 50 | TA |

| | | | | | | | | |
|---|---|---|---|---|---|---|---|---|
| 579 | chr11 | 18746777 | 18748777 | 11.53846 | 0.047866 | IGSF22 | immunoglobulin superfamily, member 22 | TM |
| 580 | chr14 | 23856875 | 23858875 | 10.10697 | 0.048608 | MIR208A | microRNA 208a | TM |
| 581 | chr15 | 35086927 | 35088927 | 11.23474 | 0.04905 | ACTC1 | actin, alpha, cardiac muscle 1 | TM |
| 582 | chr16 | 67225067 | 67227067 | -10.9158 | 0.049669 | E2F4 | E2F transcription factor 4, p107/p130-binding | TA |
| 583 | chr20 | 39968492 | 39970492 | 14.14352 | 0.049669 | LPIN3 | lipin 3 | TM |
| 584 | chr7 | 143078778 | 143080778 | -17.5471 | 0.049833 | MIR6892 | MicroRNA 6892 | TA |

**Supplementary Table 1.** Differentially methylated promoters between aortic and mitral valve tissue (q value < 0.05 and a meth.diff > |10|) and their associated genes ordered by significance (q-value). There is a total of 584 promoters that are differentially methylated between aortic and mitral valve tissue. prom.chr, prom.start and prom.end identify the location of each promoter. meth.diff represents the difference between the weighted average of the percent methylation values of aortic (TA) and mitral (TM) samples (**see Methods**). qvalue denotes the corrected statistical significance of this difference (**see Methods**). assoc.gene-Symbol represents the gene associated with the corresponding differentially methylated promoter. Finally, dir identifies the tissue (aortic (TA)/ mitral (TM) tissue) in which the particular promoter exhibits increased methylation.

## 2.2 Table S2. Functional Classification based on PANTHER Pathways for the genes associated with differentially methylated promoters between the non-diseased aortic and mitral valve tissue – full list

| No. | PANTHER Pathway | No. of genes | genes (Symbol) |
|---|---|---|---|
| 1 | Heterotrimeric G-protein signaling pathway-Gi alpha and Gs alpha mediated pathway (P00026) | 8 | RGS3, RGS9, HTR5A, HTR2A, ADCY7, SSTR3, RGS14, GNAI2 |
| 2 | **Inflammation mediated by chemokine and cytokine signaling pathway (P00031)** | 6 | PAK6, ACTC1, CASK, BCL3, RGS14, GNAI2 |
| 3 | Alzheimer disease-presenilin pathway (P00004) | 5 | TRIM2, ACTC1, WNT5B, CD44, TRPC3 |
| 4 | **Wnt signaling pathway (P00057)** | 5 | AES, ACTC1, PCDHA5, WNT5B, CDH4 |
| 5 | Nicotinic acetylcholine receptor signaling pathway (P00044) | 5 | ACTC1, VAMP1, MYO15A, SLC6A8, MYO7B |
| 6 | Gonadotropin-releasing hormone receptor pathway (P06664) | 5 | LHB, SMAD3, PCP4, NOS1, GNAI2 |
| 7 | Huntington disease (P00029) | 4 | PACSIN1, ARL4D, |

| | | | |
|---|---|---|---|
| | | | ACTC1, KALRN |
| 8 | Heterotrimeric G-protein signaling pathway-Gq alpha and Go alpha mediated pathway (P00027) | 4 | RGS3, RGS9, SSTR3, RGS14 |
| 9 | **Cadherin signaling pathway (P00012)** | 4 | ACTC1, PCDHA5, WNT5B, CDH4 |
| 10 | **5HT1 type receptor mediated signaling pathway (P04373)** | 3 | VAMP1, ADCY7, GNAI2 |
| 11 | **TGF-beta signaling pathway (P00052)** | 3 | SMAD3, FOXH1, TAB1 |
| 12 | Opioid proopiomelanocortin pathway (P05917) | 3 | VAMP1, ADCY7, GNAI2 |
| 13 | **Cytoskeletal regulation by Rho GTPase (P00016)** | 3 | PAK6, ACTC1, LIMK1 |
| 14 | Opioid prodynorphin pathway (P05916) | 3 | VAMP1, ADCY7, GNAI2 |
| 15 | Opioid proenkephalin pathway (P05915) | 3 | VAMP1, ADCY7, GNAI2 |
| 16 | Muscarinic acetylcholine receptor 2 and 4 signaling pathway (P00043) | 3 | VAMP1, SLC6A8, GNAI2 |
| 17 | Dopamine receptor mediated signaling pathway (P05912) | 3 | VAMP1, ADCY7, GNAI2 |
| 18 | Beta3 adrenergic receptor signaling pathway (P04379) | 2 | VAMP1, ADCY7 |
| 19 | Metabotropic glutamate receptor group III pathway (P00039) | 2 | VAMP1, GNAI2 |
| 20 | Beta2 adrenergic receptor signaling pathway (P04378) | 2 | VAMP1, ADCY7 |

| | | | |
|---|---|---|---|
| 21 | Beta1 adrenergic receptor signaling pathway (P04377) | 2 | VAMP1, ADCY7 |
| 22 | **Apoptosis signaling pathway (P00006)** | 2 | CASP7, TNFRSF10C |
| 23 | Ionotropic glutamate receptor pathway (P00037) | 2 | VAMP1, SHANK2 |
| 24 | **5HT4 type receptor mediated signaling pathway (P04376)** | 2 | VAMP1, ADCY7 |
| 25 | **Angiogenesis (P00005)** | 2 | F2R, WNT5B |
| 26 | **5HT2 type receptor mediated signaling pathway (P04374)** | 2 | VAMP1, HTR2A |
| 27 | **Integrin signalling pathway (P00034)** | 2 | ACTC1, COL5A3 |
| 28 | Adrenaline and noradrenaline biosynthesis (P00001) | 2 | VAMP1, PNMT |
| 29 | Synaptic vesicle trafficking (P05734) | 2 | VAMP1, SYN1 |
| 30 | Vitamin D metabolism and pathway (P04396) | 2 | DBP, RXRA |
| 31 | T cell activation (P00053) | 2 | ZAP70, LCP2 |
| 32 | **Endothelin signaling pathway (P00019)** | 2 | ADCY7, ECE2 |
| 33 | Parkinson disease (P00049) | 2 | CASK, FGR |
| 34 | **PDGF signaling pathway (P00047)** | 2 | ARHGAP27, RPS6KL1 |
| 35 | Enkephalin release (P05913) | 2 | ADCY7, GNAI2 |
| 36 | **Blood coagulation (P00011)** | 2 | F2R, F10 |
| 37 | Cortocotropin releasing factor receptor signaling pathway (P04380) | 2 | CRH, VAMP1 |
| 38 | Metabotropic glutamate receptor group II pathway (P00040) | 2 | VAMP1, GNAI2 |
| 39 | Axon guidance mediated by netrin (P00009) | 1 | ABLIM1 |
| 40 | **5HT3 type receptor mediated signaling pathway (P04375)** | 1 | VAMP1 |
| 41 | Coenzyme A biosynthesis (P02736) | 1 | COASY |
| 42 | Alpha adrenergic receptor signaling pathway (P00002) | 1 | VAMP1 |

| | | | |
|---|---|---|---|
| 43 | Ubiquitin proteasome pathway (P00060) | 1 | SAE1 |
| 44 | GABA-B receptor II signaling (P05731) | 1 | ADCY7 |
| 45 | Heterotrimeric G-protein signaling pathway-rod outer segment phototransduction (P00028) | 1 | RGS9 |
| 46 | p53 pathway feedback loops 2 (P04398) | 1 | RBL1 |
| 47 | Pyridoxal-5-phosphate biosynthesis (P02759) | 1 | PNPO |
| 48 | Androgen/estrogene/progesterone biosynthesis (P02727) | 1 | HSD3B7 |
| 49 | **Transcription regulation by bZIP transcription factor (P00055)** | 1 | TAF8 |
| 50 | Thyrotropin-releasing hormone receptor signaling pathway (P04394) | 1 | VAMP1 |
| 51 | **General transcription regulation (P00023)** | 1 | TAF8 |
| 52 | Vitamin B6 metabolism (P02787) | 1 | PNPO |
| 53 | Toll receptor signaling pathway (P00054) | 1 | TAB1 |
| 54 | **Methylmalonyl pathway (P02755)** | 1 | MCCC2 |
| 55 | **General transcription by RNA polymerase I (P00022)** | 1 | TAF8 |
| 56 | Acetate utilization (P02722) | 1 | ACSS1 |
| 57 | Oxytocin receptor mediated signaling pathway (P04391) | 1 | VAMP1 |
| 58 | FAS signaling pathway (P00020) | 1 | CASP7 |
| 59 | p38 MAPK pathway (P05918) | 1 | TAB1 |
| 60 | DNA replication (P00017) | 1 | TOP1MT |
| 61 | PI3 kinase pathway (P00048) | 1 | GNAI2 |
| 62 | Histamine H2 receptor mediated signaling pathway (P04386) | 1 | ADCY7 |
| 63 | **Notch signaling pathway (P00045)** | 1 | AC018470 |
| 64 | Heme biosynthesis (P02746) | 1 | EPRS |
| 65 | Succinate to proprionate conversion (P02777) | 1 | MCCC2 |
| 66 | Salvage pyrimidine ribonucleotides (P02775) | 1 | UCKL1 |
| 67 | Muscarinic acetylcholine receptor 1 and 3 signaling pathway (P00042) | 1 | VAMP1 |
| 68 | CCKR signaling map (P06959) | 1 | NOS1 |
| 69 | Pyridoxal phosphate salvage pathway (P02770) | 1 | PNPO |

**Supplementary Table 2.** PANTHER Pathways classifying the genes associated with differentially methylated promoters between the non-diseased aortic and mitral valve tissue. Pathways of interest

are indicated in bold. The methylation direction of the pathways' constituent genes can be found in **Supplementary Table 1**.

**2.3 Table S3. Functional Classification based on PANTHER Molecular Functions for the genes associated with differentially methylated promoters between the non-diseased aortic and mitral valve tissue – full list**

| No. | PANTHER Molecular Function | No. of genes | genes (Symbol) |
|---|---|---|---|
| 1 | **binding (GO:0005488)** | 103 | NDE1, PACSIN1, HOXB3, AES, GSPT1, AC131097, HLA-C, TOP1MT, RAD51C, HN1L, NR2F2, DNM1L, SEMA4B, ARL4D, RPL22L1, HTR5A, DMTN, ACTC1, EPS8L1, DBP, RBL1, GRTP1, LEO1, TBC1D10C, SETX, ZNF597, ABLIM1, IL17RE, RPL13A, LHB, VAMP1, APOA5, SPA17, REM2, UNK, RXRA, TBR1, THRA, DBP, MT1L, RPL11, LYNX1, CTC1, SEPT9, HTR2A, PPP4R2, UBOX5, ANGEL2, CLUL1, OSBPL1A, PPIE, RPS3, PCK2, DYSF, WNT5B, NGFR, FAM115A, TACR2, ATL3, PDLIM4, MT1A, ZNF497, PABPC4L, CYP2E1, AOX1, IPO4, TUBA8, CELF3, SPEF1, CD44, SHBG, IFNLR1, NOS1, FGR, ZAP70, ANPEP, FGFBP2, PLTP, AMBP, ZNF169, COLEC11, SSTR3, NPPB, CORO1B, CARHSP1, E2F4, HSPA7, PGF, TRPC3, MDK, LSM2, SBNO2, CDC6, CYB5R1, ZNF259, ENPEP, HOMER3, GNAI2, DDR1, SHANK2, PCGF3, MT1F, CGB7 |

| | | | |
|---|---|---|---|
| 2 | **catalytic activity (GO:0003824)** | 77 | PAK6, ATP6V1F, VKORC1L1, EPRS, PRPSAP2, GSPT1, TYSND1, TRIM2, TOP1MT, PDE4A, RAD51C, GINS2, CTSZ, HAS1, CASP7, DNM1L, LPIN3, NEURL3, ELOVL3, DYRK1B, DHRS3, GRTP1, TBC1D10C, GSTO2, GINS4, APOA5, PRSS33, SEPT9, PPP4R2, UBOX5, ANGEL2, TGM2, UBE4B, PPIE, RPS3, PCK2, SRPK3, CWF19L1, SBK2, PNMT, NUDT7, ACER3, ATL3, ADCY7, CYP2E1, UCKL1, AOX1, LIMK1, NQO1, NUDT22, TAB1, ISG20, SAE1, NOS1, FGR, ZAP70, ANPEP, MCCC2, ERCC8, SUPT7L, SIRT6, AMBP, FUCA2, NEURL3, HSPA7, POLR1C, COASY, ATP6V1G1, PLK4, C19orf35, MAN2A2, ENPEP, GNAI2, DDR1, MAEA, HSD3B7, TOR4A |
| 3 | transcription regulator activity (GO:0140110) | 21 | HOXB3, AES, CCAR1, NR2F2, LPIN3, DYRK1B, AC018470.4, ZNF597, RXRA, TBR1, ATXN7L3, THRA, ZNF497, FOXL1, SUPT7L, SIRT6, ZNF169, E2F4, FHL2, ZMYM1, PCGF3 |
| 4 | **molecular function regulator (GO:0098772)** | 17 | PRSS41, FXYD2, SEMA4B, EPS8L1, GRTP1, TBC1D10C, LHB, APOA5, REM2, LYNX1, PPP4R2, CWF19L1, AMBP, NPPB, PGF, MDK, CGB7 |
| 5 | transporter activity (GO:0005215) | 17 | ATP6V1F, PRSS41, SLC12A6, RANBP17, FXYD2, SLC7A8, REM2, SLC16A3, OSBPL1A, P2RX1, IPO4, SLC35A1, KCNH2, PLTP, SLC10A2, TRPC3, ATP6V1G1 |

| | | | |
|---|---|---|---|
| 6 | molecular transducer activity (GO:0060089) | 15 | HTR5A, IL17RE, THRA, LYNX1, F2R, HTR2A, NGFR, TACR2, CD44, IFNLR1, RGMA, RAMP1, GPR35, SSTR3, DDR1 |
| 7 | structural molecule activity (GO:0005198) | 8 | MRPS17, RPL22L1, RPL13A, RPL11, RPS3, MRPL15, TUBA8, MRPL15 |
| 8 | translation regulator activity (GO:0045182) | 1 | GSPT1 |

**Supplementary Table 3.** PANTHER Molecular Functions classifying the genes associated with differentially methylated promoters between the non-diseased aortic and mitral valve tissue. Molecular functions of interest are indicated in bold. The methylation direction of the molecular functions' constituent genes can be found in **Supplementary Table 1.**

## 2.4 Table S4. Functional Classification based on PANTHER Biological Processes for the genes associated with differentially methylated promoters between the non-diseased aortic and mitral valve tissue – full list

| No. | PANTHER Biological Process | No. of genes | genes (Symbol) |
|---|---|---|---|
| 1 | cellular process (GO:0009987) | 161 | NDE1, PAK6, PACSIN1, IBA57, HOXB3, VKORC1L1, EPRS, AES, PRPSAP2, GSPT1, AC131097.3, SLC12A6, SYCP2L, CCAR1, TRIM2, TOP1MT, RAD51C, MRPS17, GINS2, CTSZ, RPS7, HAS1, CASP7, NR2F2, DNM1L, IQSEC1, C2orf40, SEMA4B, TCTN2, GPR68, LPIN3, ELOVL3, RPL22L1, HTR5A, EXOC7, DMTN, DYRK1B, DHRS3, ACTC1, EPS8L1, RBL1, LEO1, AC018470.4, ZNF597, ABLIM1, GINS4, RPL13A, LHB, VAMP1, APOA5, TCTN1, UNK, RXRA, TBR1, ATXN7L3, THRA, TBC1D32, MT1L, DNASE1L1, TRIP6, RPL11, F2R, DMKN, CTC1, SEPT9, TMEM101, HTR2A, PPP4R2, UBOX5, ANGEL2, TGM2, UBE4B, PPIE, RPS3, PCK2, SRPK3, LRFN3, DYSF, WNT5B, CWF19L1, IQSEC3, SBK2, NUDT7, NGFR, XAB2, TACR2, ATL3, PDLIM4, MT1A, WDTC1, ZNF497, VPS51, ADCY7, CYP2E1, HTR5A, BTBD2, PRPF6, MAGOH, AOX1, TUBA8, LIMK1, FBF1, CELF3, FOXL1, TRA2A, SPEF1, DRC1, TAB1, IFNLR1, ISG20, SAE1, NOS1, FGR, ZAP70, ANPEP, RGMA, FGFBP2, RAMP1, TEKT2, MCCC2, ERCC8, PLTP, SUPT7L, GPR35, SIRT6, SSTR3, NPPB, CORO1B, CARHSP1, TANGO6, NEURL3, E2F4, MZB1, GINS1, PSAP, HSPA7, PGF, ZMYM1, KANK3, RHBDF1, BRCA2, TRPC3, COASY, LSM2, PLK4, SBNO2, CDC6, C19orf35, ZNF259, MAN2A2, ENPEP, TNFRSF10C, SYN1, HOMER3, GNAI2, LCP2, SHANK2, MAEA, PCGF3, MT1F, CGB7 |
| 2 | **biological regulation (GO:0065007)** | 98 | PAK6, PACSIN1, HOXB3, AES, SLC12A6, CCAR1, HLA-C, CASK, FXYD2, NR2F2, DNM1L, C2orf40, SEMA4B, TCTN2, LPIN3, HTR5A, DYRK1B, DHRS3, ARHGAP27, EPS8L1, RBL1, GRTP1, LEO1, TBC1D10C, AC018470.4, ZNF597, RPL13A, LHB, APOA5, RXRA, TBR1, ATXN7L3, THRA, MT1L, TRIP6, F2R, CTC1, TMEM101, HTR2A, ANGEL2, RPS3, PCK2, SRPK3, LRFN3, DYSF, WNT5B, SBK2, NGFR, FAM115A, TACR2, MT1A, WDTC1, ZNF497, ADCY7, LIMK1, CELF3, FOXL1, TRA2A, SPEF1, DRC1, TAB1, IFNLR1, ISG20, KCNH2, NOS1, FGR, ZAP70, RGMA, RAMP1, ERCC8, PLTP, GPR35, SIRT6, SSTR3, NPPB, CARHSP1, E2F4, MZB1, PSAP, PGF, ZMYM1, KANK3, |

| | | | |
|---|---|---|---|
| | | | RHBDF1, BRCA2, TRPC3, PLK4, SBNO2, CDC6, ZNF259, TNFRSF10C, SYN1, HOMER3, GNAI2, LCP2, SHANK2, PCGF3, MT1F, CGB7 |
| 3 | metabolic process (GO:0008152) | 96 | PAK6, DDX19B, IBA57, HOXB3, VKORC1L1, EPRS, PRPSAP2, GSPT1, TYSND1, CCAR1, TRIM2, TOP1MT, RAD51C, MRPS17, GINS2, CTSZ, RPS7, HAS1, NR2F2, DNM1L, C2orf40, LPIN3, ELOVL3, RPL22L1, DYRK1B, DHRS3, LEO1, AC018470.4, ZNF597, GSTO2, GINS4, RPL13A, APOA5, PRSS33, RXRA, TBR1, ATXN7L3, THRA, DNASE1L1, TRIP6, CTC1, TMEM101, PPP4R2, UBOX5, ANGEL2, TGM2, UBE4B, RPS3, PCK2, SRPK3, CWF19L1, SBK2, NUDT7, XAB2, WDTC1, ZNF497, CYP2E1, PRPF6, MAGOH, AOX1, CELF3, FOXL1, TRA2A, TAB1, ISG20, SAE1, NOS1, FGR, ZAP70, ANPEP, RGMA, RAMP1, MCCC2, ERCC8, SUPT7L, SIRT6, PM20D1, NPPB, CARHSP1, FUCA2, E2F4, MZB1, GINS1, PSAP, PGF, ZMYM1, BRCA2, COASY, LSM2, SBNO2, C19orf35, ZNF259, MAN2A2, ENPEP, MAEA, PCGF3 |
| 4 | **cellular component organization or biogenesis (GO:0071840)** | 64 | NDE1, PAK6, PACSIN1, IBA57, GSPT1, AC131097.3, SLC12A6, SYCP2L, TOP1MT, RAD51C, RPS7, HAS1, DNM1L, IQSEC1, SEMA4B, TCTN2, EXOC7, DMTN, ACTC1, EPS8L1, RBL1, ABLIM1, VAMP1, APOA5, TCTN1, UNK, TBR1, ATXN7L3, TBC1D32, RPL11, DMKN, CTC1, SEPT9, SRPK3, LRFN3, DYSF, IQSEC3, XAB2, ATL3, PDLIM4, VPS51, HTR5A, PRPF6, TUBA8, LIMK1, FBF1, CELF3, SPEF1, DRC1, RAMP1, TEKT2, PLTP, SUPT7L, SIRT6, CORO1B, NEURL3, GINS1, ZMYM1, KANK3, PLK4, CDC6, ZNF259, SHANK2, PCGF3 |
| 5 | **response to stimulus (GO:0050896**) | 58 | PAK6, AES, AC131097.3, HLA-C, RAD51C, GINS2, CASK, C2orf40, SEMA4B, TCTN2, GPR68, LPIN3, HTR5A, DHRS3, EPS8L1, GINS4, LHB, RXRA, THRA, MT1L, TRIP6, F2R, TMEM101, HTR2A, UBE4B, RPS3, PCK2, SRPK3, DYSF, WNT5B, SBK2, NGFR, MT1A, ADCY7, CYP2E1, IFNLR1, ISG20, NOS1, FGR, ZAP70, RGMA, RAMP1, ERCC8, GPR35, SSTR3, NPPB, PSAP, HSPA7, PGF, RHBDF1, BRCA2, TNFRSF10C, HOMER3, GNAI2, LCP2, SHANK2, MT1F, CGB7 |
| 6 | localization (GO:0051179) | 54 | NDE1, DDX19B, PACSIN1, AC131097.3, SLC12A6, SLC9B1, CASK, RANBP17, FXYD2, DNM1L, SLC14A1, SEMA4B, TCTN2, ARL4D, EXOC7, ACTC1, DBP, GRTP1, TBC1D10C, VAMP1, APOA5, TCTN1, SLC7A8, SLC6A8, TBC1D32, SLC16A3, SEPT9, HTR2A, DYSF, FAM115A, TACR2, VPS51, HTR5A, IPO4, FBF1, DRC1, |

| | | | |
|---|---|---|---|
| | | | KCNH2, RAMP1, TEKT2, PLTP, AP1S3, SLC10A2, CORO1B, TANGO6, NEURL3, HSPA7, PGF, RHBDF1, TRPC3, ZNF259, SLC51A, SYN1, HOMER3, SHANK2 |
| 7 | signaling (GO:0023052) | 41 | PAK6, AES, SLC12A6, SEMA4B, TCTN2, HTR5A, DHRS3, EPS8L1, LHB, RXRA, THRA, TRIP6, F2R, TMEM101, HTR2A, RPS3, SRPK3, WNT5B, SBK2, NGFR, ADCY7, IFNLR1, NOS1, FGR, ZAP70, RGMA, FGFBP2, RAMP1, GPR35, SSTR3, NPPB, PSAP, PGF, RHBDF1, TNFRSF10C, SYN1, HOMER3, GNAI2, LCP2, SHANK2, CGB7 |
| 8 | **developmental process (GO:0032502**) | 26 | PACSIN1, HOXB3, CASK, NR2F2, C2orf40, SEMA4B, RBL1, UNK, RXRA, TBR1, THRA, PCK2, LRFN3, DYSF, WNT5B, PDLIM4, BTBD2, FOXL1, FGR, ZAP70, RAMP1, PSAP, PGF, ZMYM1, ZNF259, SHANK2 |
| 9 | multicellular organismal process (GO:0032501) | 26 | PACSIN1, HOXB3, AC131097.3, CASK, C2orf40, SEMA4B, APOA5, UNK, TBR1, THRA, PCK2, LRFN3, WNT5B, PDLIM4, BTBD2, CLEC11A, NOS1, RAMP1, PLTP, NPPB, PSAP, USH1C, PGF, PDZD7, ZNF259, SHANK2 |
| 10 | locomotion (GO:0040011) | 7 | NDE1, SEMA4B, TACR2, DRC1, TEKT2, CORO1B, PGF |
| 11 | reproduction (GO:0000003) | 5 | SYCP2L, RAD51C, TACR2, PSAP, TRPC3 |
| 12 | cell population proliferation (GO:0008283) | 5 | C2orf40, FGR, ZAP70, MZB1, PGF |
| 13 | reproductive process (GO:0022414) | 5 | SYCP2L, RAD51C, TACR2, PSAP, TRPC3 |
| 14 | immune system process (GO:0002376) | 5 | HLA-C, DYSF, ISG20, MZB1, PGF |
| 15 | multi-organism process (GO:0051704) | 4 | ISG20, NOS1, TRPC3, SHANK2 |
| 16 | **biological adhesion (GO:0022610)** | 3 | LRFN3, PCDHA5, SPON2 |
| 17 | growth (GO:0040007) | 3 | SEMA4B, PSAP, SHANK2 |

| | | | |
|---|---|---|---|
| 18 | biological phase (GO:0044848) | 2 | SYCP2L, RAD51C |
| 19 | behavior (GO:0007610) | 1 | SHANK2 |

**Supplementary Table 4.** PANTHER Biological Processes classifying the genes associated with differentially methylated promoters between the non-diseased aortic and mitral valve tissue. Biological processes of interest are indicated in bold. The methylation direction of the biological processes' constituent genes can be found in **Supplementary Table 1.**

**2.5 Table S5. Functional Classification based on PANTHER Protein Classes for the genes associated with differentially methylated promoters between the non-diseased aortic and mitral valve tissue – full list**

| No. | PANTHER Protein Class | No. of genes | genes (Symbol) |
|---|---|---|---|
| 1 | metabolite interconversion enzyme (PC00262) | 29 | VKORC1L1, EPRS, PRPSAP2, PNPO, HAS1, PIP5KL1, ELOVL3, ACSL5, SERHL2, GSTK1, ACSS1, TGM2, PCK2, PNMT, NUDT7, PIGL, CASK, UCKL1, HSD17B14, SERHL2, AOX1, NOS1, PLBD2, MCCC2, FUCA2, COASY, GANAB, LDHA, HSD3B7 |
| 2 | protein modifying enzyme (PC00260) | 29 | PRSS41, TYSND1, TRIM2, CTSZ, CASP7, MID2, NEURL3, DYRK1B, RPS6KL1, PRSS33, DCAF8, PRSS55, UBE4B, DPP6, SRPK3, F10, ADAM30, SPRYD4, WDTC1, BTBD2, ATL3, TAB1, SAE1, ANPEP, NEURL3, RHBDF1, C19orf35, ECE2, ENPEP |
| 3 | **gene-specific transcriptional regulator (PC00264)** | 21 | AES, NR2F2, AC018470.4, ZNF597, SMAD3, RXRA, TBR1, THRA, DBP, ZNF44, PBX4, ZNF497, TCF21, NMI, ZNF613, FOXL1, ZNF169, FHL2, ZMYM1, ZNF707, NCOA4 |
| 4 | **cytoskeletal protein (PC00085)** | 20 | PACSIN1, DMTN, ACTC1, ABLIM1, RIBC2, MYO15A, TRIP6, SEPT9, MYL2, PDLIM4, TUBA8, TEKT2, CRIP3, CORO1B, DCTN4, USH1C, KANK3, PDZD7, PDCD6, MYO7B |
| 5 | **nucleic acid binding protein (PC00171)** | 20 | NOS1, TOP1MT, GINS2, HN1L, ZCCHC4, LEO1, SETX, SPOCD1, ANGEL2, XAB2, PRPF6, ISG20, CARHSP1, E2F4, GINS1, BRCA2, POLR1C, G2E3, LSM2, CDC6 |
| 6 | transporter (PC00227) | 18 | ATP6V1F, RANBP17, FXYD2, NUP210L, ABCD1, SLC6A8, SLC16A3, SLC17A9, AQP2, P2RX1, ATP5J2, IPO4, SLC35A1, SLC10A2, TRPC3, ATP6V1G1, SLC51A, SLC37A4 |
| 7 | protein-binding activity modulator (PC00095) | 15 | ARL4D, ARHGAP27, GRTP1, TBC1D10C, PRKCSH, FGD3, KALRN, FGD1, SIPA1L1, ATL3, PHACTR4, CDK2AP1, ARHGEF25, AC018470.4, GNAI2 |
| 8 | **translational protein (PC00263)** | 9 | GSPT1, MRPS17, RPS7, RPL22L1, RPL13A, RPL11, RPS3, MRPL15, MRPL15 |
| 9 | transmembrane signal receptor (PC00197) | 9 | GPR68, HTR5A, HTR2A, CD44, IFNLR1, RAMP1, SSTR3, FGFRL1, GPER1 |

| | | | |
|---|---|---|---|
| 10 | intercellular signal molecule (PC00207) | 8 | SEMA4B, LHB, CRH, WNT5B, CLEC11A, PGF, MDK, CGB7 |
| 11 | membrane traffic protein (PC00150) | 7 | DNM1L, EXOC7, AP1M2, DYSF, BCL3, AP1S3, SYN1 |
| 12 | **chromatin/chromatin-binding, or -regulatory protein (PC00077)** | 5 | SYCP2L, CCAR1, RBL1, L3MBTL1, PCGF3 |
| 13 | scaffold/adaptor protein (PC00226) | 4 | GAB4, EPS8L1, PSAP, LCP2 |
| 14 | **cell junction protein (PC00070)** | 2 | GJD3, TJP2 |
| 15 | structural protein (PC00211) | 2 | STPG1, COLEC11 |
| 16 | **extracellular matrix protein (PC00102)** | 1 | COL5A3 |
| 17 | cell adhesion molecule (PC00069) | 1 | SPON2 |
| 18 | **calcium-binding protein (PC00060)** | 1 | DRC1 |
| 19 | defense/immunity protein (PC00090) | 1 | KIR2DL4 |
| 20 | transfer/carrier protein (PC00219) | 1 | DBP |
| 21 | chaperone (PC00072) | 1 | TOR4A |
| 22 | storage protein (PC00210) | 1 | CASK |

**Supplementary Table 5.** PANTHER Protein Classes classifying the genes associated with differentially methylated promoters between the non-diseased aortic and mitral valve tissue. Protein classes of interest are indicated in bold. The methylation direction of the protein classes' constituent genes can be found in **Supplementary Table 1.**

**2.6 Table S6. Functional Classification based on PANTHER Cellular Components for the genes associated with differentially methylated promoters between the non-diseased aortic and mitral valve tissue – full list**

| No. | PANTHER Cellular Component | No. of genes | genes (Symbol) |
|---|---|---|---|
| 1 | cell (GO:0005623) | 180 | NDE1, PAK6, DDX19B, PACSIN1, HOXB3, EPRS, AES, PRPSAP2, GSPT1, SYCP2L, TYSND1, CCAR1, TOP1MT, PDE4A, RAD51C, MRPS17, GINS2, RANBP17, CTSZ, RPS7, HAS1, CASP7, HN1L, NR2F2, DNM1L, SEMA4B, TCTN2, ARL4D, LPIN3, ELOVL3, HTR5A, EXOC7, DMTN, DYRK1B, DHRS3, ARHGAP27, ACTC1, EPS8L1, NUP210L, DBP, RBL1, LEO1, AC018470.4, ABLIM1, GINS4, ACRBP, RPL13A, LHB, VAMP1, TCTN1, REM2, SLC6A8, RXRA, TBR1, ATXN7L3, THRA, DBP, CD164L2, MT1L, DNASE1L1, TRIP6, DCAF8, RPL11, LYNX1, F2R, SLC16A3, CTC1, SEPT9, PLIN4, HTR2A, PPP4R2, UBOX5, TGM2, CLUL1, OSBPL1A, AQP2, UBE4B, RPS3, PCK2, SRPK3, LRFN3, PCDHA5, DYSF, CWF19L1, PNMT, NGFR, XAB2, FAM115A, TACR2, ACER3, PDLIM4, MT1A, WDTC1, ZNF497, VPS51, PABPC4L, ADCY7, CYP2E1, PIH1D1, HTR5A, NMI, BTBD2, P2RX1, PRPF6, MAGOH, AOX1, IPO4, TUBA8, LIMK1, NQO1, FBF1, CELF3, FOXL1, TRA2A, SPEF1, SLC35A1, CD44, DRC1, IFNLR1, NIPSNAP1, ISG20, KCNH2, SAE1, NOS1, FGR, ZAP70, ANPEP, UBR7, RGMA, RAMP1, TEKT2, ERCC8, SUPT7L, GPR35, SIRT6, AP1S3, AMBP, ZFYVE28, ZNF169, SLC10A2, SSTR3, NPPB, CARHSP1, FUCA2, DCTN4, RUSC1, NEURL3, E2F4, FHL2, MZB1, GINS1, HSPA7, USH1C, ZMYM1, KANK3, RHBDF1, POLR1C, TRPC3, ATP6V1G1, PDZD7, LSM2, PLK4, SBNO2, CYB5R1, C19orf35, ZNF259, MAN2A2, ENPEP, TNFRSF10C, SLC51A, SYN1, HOMER3, GNAI2, SHANK2, MAEA, PCGF3, TOR4A, MT1F, PXMP4, CGB7 |
| 2 | cell part (GO:0044464) | 180 | NDE1, PAK6, DDX19B, PACSIN1, HOXB3, EPRS, AES, PRPSAP2, GSPT1, SYCP2L, TYSND1, CCAR1, TOP1MT, PDE4A, RAD51C, MRPS17, GINS2, RANBP17, CTSZ, RPS7, HAS1, CASP7, HN1L, NR2F2, DNM1L, SEMA4B, TCTN2, ARL4D, LPIN3, ELOVL3, HTR5A, EXOC7, DMTN, DYRK1B, DHRS3, ARHGAP27, ACTC1, EPS8L1, NUP210L, DBP, RBL1, LEO1, AC018470.4, ABLIM1, GINS4, ACRBP, RPL13A, LHB, VAMP1, TCTN1, REM2, SLC6A8, RXRA, TBR1, ATXN7L3, THRA, DBP, CD164L2, MT1L, DNASE1L1, |

| | | | |
|---|---|---|---|
| | | | TRIP6, DCAF8, RPL11, LYNX1, F2R, SLC16A3, CTC1, SEPT9, PLIN4, HTR2A, PPP4R2, UBOX5, TGM2, CLUL1, OSBPL1A, AQP2, UBE4B, RPS3, PCK2, SRPK3, LRFN3, PCDHA5, DYSF, CWF19L1, PNMT, NGFR, XAB2, FAM115A, TACR2, ACER3, PDLIM4, MT1A, WDTC1, ZNF497, VPS51, PABPC4L, ADCY7, CYP2E1, PIH1D1, HTR5A, NMI, BTBD2, P2RX1, PRPF6, MAGOH, AOX1, IPO4, TUBA8, LIMK1, NQO1, FBF1, CELF3, FOXL1, TRA2A, SPEF1, SLC35A1, CD44, DRC1, IFNLR1, NIPSNAP1, ISG20, KCNH2, SAE1, NOS1, FGR, ZAP70, ANPEP, UBR7, RGMA, RAMP1, TEKT2, ERCC8, SUPT7L, GPR35, SIRT6, AP1S3, AMBP, ZFYVE28, ZNF169, SLC10A2, SSTR3, NPPB, CARHSP1, FUCA2, DCTN4, RUSC1, NEURL3, E2F4, FHL2, MZB1, GINS1, HSPA7, USH1C, ZMYM1, KANK3, RHBDF1, POLR1C, TRPC3, ATP6V1G1, PDZD7, LSM2, PLK4, SBNO2, CYB5R1, C19orf35, ZNF259, MAN2A2, ENPEP, TNFRSF10C, SLC51A, SYN1, HOMER3, GNAI2, SHANK2, MAEA, PCGF3, TOR4A, MT1F, PXMP4, CGB7 |
| 3 | organelle (GO:0043226) | 127 | NDE1, DDX19B, PACSIN1, HOXB3, AES, SYCP2L, TYSND1, CCAR1, TOP1MT, RAD51C, MRPS17, GINS2, RANBP17, CTSZ, RPS7, HN1L, NR2F2, DNM1L, TCTN2, LPIN3, ELOVL3, DMTN, DYRK1B, DHRS3, ACTC1, EPS8L1, NUP210L, RBL1, LEO1, AC018470.4, ABLIM1, GINS4, ACRBP, RPL13A, TCTN1, RXRA, TBR1, ATXN7L3, THRA, DBP, CD164L2, MT1L, DNASE1L1, TRIP6, RPL11, CTC1, SEPT9, PPP4R2, UBOX5, TGM2, CLUL1, OSBPL1A, UBE4B, RPS3, PCK2, SRPK3, LRFN3, DYSF, CWF19L1, XAB2, TACR2, ACER3, PDLIM4, MT1A, ZNF497, VPS51, PABPC4L, CYP2E1, HTR5A, BTBD2, P2RX1, PRPF6, MAGOH, IPO4, TUBA8, LIMK1, FBF1, CELF3, FOXL1, TRA2A, SPEF1, SLC35A1, DRC1, NIPSNAP1, ISG20, SAE1, NOS1, TEKT2, ERCC8, SUPT7L, SIRT6, AP1S3, ZFYVE28, ZNF169, NPPB, FUCA2, DCTN4, RUSC1, NEURL3, E2F4, FHL2, MZB1, GINS1, HSPA7, USH1C, ZMYM1, KANK3, RHBDF1, POLR1C, ATP6V1G1, PDZD7, LSM2, PLK4, SBNO2, CYB5R1, C19orf35, ZNF259, MAN2A2, SLC51A, SYN1, HOMER3, SHANK2, MAEA, PCGF3, TOR4A, MT1F, PXMP4 |
| 4 | membrane (GO:0016020) | 69 | PACSIN1, RANBP17, HAS1, DNM1L, SEMA4B, TCTN2, ARL4D, ELOVL3, HTR5A, EPS8L1, NUP210L, ACRBP, VAMP1, TCTN1, REM2, SLC6A8, LYNX1, F2R, SLC16A3, PLIN4, HTR2A, AQP2, LRFN3, |

| | | | |
|---|---|---|---|
| | | | PCDHA5, DYSF, NGFR, FAM115A, TACR2, ACER3, VPS51, ADCY7, HTR5A, P2RX1, FBF1, SPEF1, SLC35A1, CD44, DRC1, IFNLR1, KCNH2, NOS1, FGR, ZAP70, ANPEP, RGMA, GPR35, AMBP, ZFYVE28, SLC10A2, SSTR3, NEURL3, MZB1, HSPA7, USH1C, RHBDF1, TRPC3, ATP6V1G1, PDZD7, ZNF259, MAN2A2, ENPEP, TNFRSF10C, SLC51A, SYN1, HOMER3, GNAI2, SHANK2, TOR4A, PXMP4 |
| 5 | organelle part (GO:0044422) | 68 | NDE1, SYCP2L, TOP1MT, RAD51C, MRPS17, GINS2, RANBP17, RPS7, DNM1L, TCTN2, ELOVL3, ACTC1, NUP210L, RBL1, GINS4, RPL13A, TCTN1, RXRA, ATXN7L3, THRA, TRIP6, RPL11, CTC1, SEPT9, RPS3, LRFN3, CWF19L1, XAB2, TACR2, ACER3, PDLIM4, ZNF497, VPS51, P2RX1, PRPF6, MAGOH, TUBA8, FBF1, TRA2A, SPEF1, SLC35A1, DRC1, SAE1, NOS1, ERCC8, SUPT7L, SIRT6, ZFYVE28, DCTN4, E2F4, FHL2, MZB1, GINS1, HSPA7, USH1C, RHBDF1, POLR1C, ATP6V1G1, PDZD7, LSM2, PLK4, ZNF259, MAN2A2, SLC51A, SYN1, PCGF3, TOR4A, PXMP4 |
| 6 | protein-containing complex (GO:0032991) | 66 | NDE1, DDX19B, EPRS, AES, PRPSAP2, GSPT1, TOP1MT, RAD51C, MRPS17, GINS2, RANBP17, RPS7, TCTN2, EXOC7, ACTC1, NUP210L, RBL1, GINS4, RPL13A, VAMP1, APOA5, TCTN1, RXRA, ATXN7L3, THRA, DCAF8, RPL11, CTC1, SEPT9, PPP4R2, RPS3, CWF19L1, XAB2, TACR2, PDLIM4, WDTC1, VPS51, PABPC4L, BTBD2, PRPF6, MAGOH, FBF1, CELF3, TRA2A, SPEF1, DRC1, SAE1, RAMP1, MCCC2, ERCC8, SUPT7L, DCTN4, E2F4, MZB1, GINS1, HSPA7, USH1C, POLR1C, TRPC3, ATP6V1G1, PDZD7, LSM2, GNAI2, SHANK2, MAEA, PCGF3 |
| 7 | membrane part (GO:0044425) | 41 | HAS1, SEMA4B, TCTN2, ELOVL3, HTR5A, EPS8L1, VAMP1, TCTN1, LYNX1, F2R, SLC16A3, HTR2A, AQP2, LRFN3, PCDHA5, DYSF, TACR2, ACER3, ADCY7, P2RX1, FBF1, SPEF1, SLC35A1, DRC1, IFNLR1, KCNH2, FGR, ZAP70, GPR35, SLC10A2, SSTR3, USH1C, RHBDF1, TRPC3, ATP6V1G1, PDZD7, ZNF259, SLC51A, HOMER3, GNAI2, SHANK2 |
| 8 | extracellular region part (GO:0044421) | 22 | CASK, CTSZ, C2orf40, SEMA4B, DBP, LHB, APOA5, PRSS33, DMKN, TGM2, CLUL1, F10, WNT5B, CLEC11A, PLTP, COLEC11, NPPB, PSAP, HSPA7, PGF, SPON2, CGB7 |
| 9 | **extracellular region (GO:0005576)** | 22 | CASK, CTSZ, C2orf40, SEMA4B, DBP, LHB, APOA5, PRSS33, DMKN, TGM2, CLUL1, F10, WNT5B, CLEC11A, PLTP, COLEC11, NPPB, PSAP, HSPA7, PGF, SPON2, CGB7 |

| | | | |
|---|---|---|---|
| 10 | membrane-enclosed lumen (GO:0031974) | 15 | SYCP2L, TOP1MT, MRPS17, GINS2, RPS7, GINS4, ATXN7L3, CTC1, ZNF497, SUPT7L, SIRT6, GINS1, POLR1C, ZNF259, TOR4A |
| 11 | supramolecular complex (GO:0099080) | 9 | TRIP6, TACR2, PDLIM4, TUBA8, SPEF1, DRC1, FHL2, USH1C, PDZD7 |
| 12 | synapse part (GO:0044456) | 4 | LRFN3, SYN1, HOMER3, SHANK2 |
| 13 | synapse (GO:0045202) | 4 | LRFN3, SYN1, HOMER3, SHANK2 |
| 14 | cell junction (GO:0030054) | 3 | TRIP6, PDLIM4, C19orf35 |

**Supplementary Table 6.** PANTHER Cellular Components classifying the genes associated with differentially methylated promoters between the non-diseased aortic and mitral valve tissue. Cellular components of interest are indicated in bold. The methylation direction of the cellular components' constituent genes can be found in **Supplementary Table 1.**

## 2.7 Table S7. Node characteristics of the network with the minimum interactions constructed for genes associated with differentially methylated promoters between non-diseased aortic and mitral valve tissue

| Node No. | Gene Name | Degree | Betweenness Centrality | Meth.diff |
|---|---|---|---|---|
| 1 | UBC | 200 | 129377.98 | 0 |
| 2 | SMAD3 | 75 | 27152.04 | 10.24797714 |
| 3 | UBL4A | 50 | 13019.92 | 13.04122333 |
| 4 | RPS3 | 50 | 9508.1 | 13.123899 |
| 5 | RXRA | 47 | 12661.72 | 12.61173363 |
| 6 | SH3KBP1 | 44 | 13005.65 | -27.60149769 |
| 7 | APP | 42 | 12097.08 | 0 |
| 8 | HNF4A | 40 | 10699.51 | 0 |
| 9 | MAGOH | 39 | 7183.66 | -11.78014905 |
| 10 | RPL11 | 36 | 3977.57 | -18.82135283 |
| 11 | RBL1 | 30 | 7986.23 | 22.97564187 |
| 12 | RPS7 | 30 | 4608.25 | -31.77081637 |
| 13 | SUMO2 | 28 | 5688.95 | 0 |
| 14 | TRIP6 | 27 | 7374.14 | -13.50530214 |
| 15 | STAU1 | 27 | 6396.77 | 13.74715119 |
| 16 | TGM2 | 27 | 5622.28 | 23.42375367 |
| 17 | TAB1 | 26 | 5178.21 | 17.01310089 |
| 18 | LDHA | 25 | 5515.63 | 14.12136251 |
| 19 | FHL2 | 24 | 4672.28 | 20.21376557 |
| 20 | KRT8 | 23 | 5003.67 | 11.90578465 |
| 21 | TRA2A | 23 | 4523 | 23.11952934 |
| 22 | ELAVL1 | 23 | 3646.08 | 0 |
| 23 | E2F4 | 22 | 4251.37 | -10.91575092 |
| 24 | UBE4B | 22 | 3845.25 | 14.3513937 |

| | | | | |
|---|---|---|---|---|
| 25 | LCP2 | 22 | 2710.17 | -24.21052632 |
| 26 | PRPF6 | 21 | 2211.77 | 22.4137931 |
| 27 | GSTK1 | 20 | 4185.53 | -15.25840084 |
| 28 | ZAP70 | 20 | 3991.93 | 12.89194545 |
| 29 | CD44 | 20 | 2575.69 | -23.95740905 |
| 30 | SHBG | 20 | 2503.5 | 26.40496882 |
| 31 | HSP90AA1 | 20 | 2310.66 | 0 |
| 32 | BCL3 | 20 | 2209.78 | -22.21153846 |
| 33 | CASP7 | 19 | 2658.38 | 12.29277823 |
| 34 | UNK | 19 | 1253.48 | -15.14692026 |
| 35 | BRCA2 | 18 | 5519.91 | 16.35676628 |
| 36 | ESR1 | 18 | 1911.01 | 0 |
| 37 | FN1 | 18 | 1677.57 | 0 |
| 38 | EGR1 | 17 | 3430.25 | 0 |
| 39 | NMI | 17 | 3321.82 | 11.66389975 |
| 40 | GRB2 | 17 | 3228.05 | 0 |
| 41 | GSPT1 | 17 | 2911.37 | 12.36027239 |
| 42 | THRA | 17 | 2613.06 | 13.74375284 |
| 43 | TP53 | 17 | 2571.84 | 0 |
| 44 | HSP90AB1 | 17 | 1488.24 | 0 |
| 45 | IPO4 | 16 | 3055.36 | 12.19300654 |
| 46 | GANAB | 16 | 2848.85 | -14.01992187 |
| 47 | GFAP | 16 | 2705.77 | -11.3620941 |
| 48 | PDCD6 | 16 | 2563.05 | 13.54642314 |
| 49 | ACTC1 | 16 | 2450.25 | 11.2347351 |
| 50 | MYC | 16 | 1993.41 | 0 |
| 51 | LEO1 | 15 | 3282.64 | 14.16123089 |
| 52 | BTBD2 | 15 | 2126.7 | 14.78212999 |

| | | | | |
|---|---|---|---|---|
| 53 | CUL3 | 15 | 1028.75 | 0 |
| 54 | GNAI2 | 14 | 3677.97 | 16.04330204 |
| 55 | LSM2 | 14 | 3531.09 | -11.79832117 |
| 56 | ATP6V1F | 14 | 2229.8 | 11.53754185 |
| 57 | NGFR | 14 | 2192.03 | -10.69046155 |
| 58 | PAK6 | 14 | 1982.42 | -25.30567215 |
| 59 | TRIM2 | 14 | 1483.96 | -22.52276867 |
| 60 | SUMO1 | 14 | 1142.11 | 0 |
| 61 | CAND1 | 14 | 991.97 | 0 |
| 62 | YWHAZ | 14 | 893.51 | 0 |
| 63 | COPS5 | 14 | 860.52 | 0 |
| 64 | TJP2 | 13 | 3141.78 | 14.3216432 |
| 65 | FGR | 13 | 3049.4 | 10.6788552 |
| 66 | RGS14 | 13 | 2147.7 | 11.14789411 |
| 67 | FBF1 | 13 | 1936.35 | 12.48030734 |
| 68 | SP1 | 13 | 1690.29 | 0 |
| 69 | YWHAQ | 13 | 1649.32 | 0 |
| 70 | POLR1C | 13 | 1534.29 | 10.54313099 |
| 71 | DYRK1B | 13 | 1401.02 | 15.87205316 |
| 72 | CDK2 | 13 | 1302.1 | 0 |
| 73 | SHANK2 | 13 | 1106.01 | 16.65018901 |
| 74 | RPL13A | 13 | 1010.94 | -11.81580267 |
| 75 | EP300 | 13 | 954.41 | 0 |
| 76 | XAB2 | 13 | 897.97 | -17.19454457 |
| 77 | FLOT2 | 12 | 2935.56 | -10.19283747 |
| 78 | CDC6 | 12 | 2482.34 | 10.7300885 |
| 79 | DNM1L | 12 | 2287.39 | 11.23387061 |
| 80 | DCAF8 | 12 | 2209.18 | 12.21130221 |

| | | | | |
|---|---|---|---|---|
| 81 | KALRN | 12 | 2184.4 | -11.43568841 |
| 82 | CCP110 | 12 | 1951.26 | 13.90348395 |
| 83 | SRC | 12 | 1451.86 | 0 |
| 84 | SUPT7L | 12 | 1368.89 | -11.85426244 |
| 85 | HSPA4 | 12 | 1217.56 | 0 |
| 86 | SRPK3 | 12 | 1065.38 | 13.3665988 |
| 87 | PACSIN1 | 12 | 683.47 | 13.28454478 |
| 88 | TRAF6 | 12 | 597.06 | 0 |
| 89 | PHYHIP | 12 | 511.72 | -18.61342387 |
| 90 | CASK | 11 | 2525.78 | 16.94493884 |
| 91 | PSAP | 11 | 1765.43 | -20.44239631 |
| 92 | UBOX5 | 11 | 1709.49 | -17.30249467 |
| 93 | ABLIM1 | 11 | 1655.14 | -26.26418989 |
| 94 | CUL1 | 11 | 1408.23 | 0 |
| 95 | CORO1B | 11 | 1264.69 | -16.60028824 |
| 96 | DDX19B | 11 | 1243.98 | -10.47878223 |
| 97 | LIMK1 | 11 | 1241.28 | 19.58291321 |
| 98 | RELA | 11 | 904.57 | 0 |
| 99 | ERCC8 | 11 | 694.93 | -12.28840789 |
| 100 | SYN1 | 11 | 559.06 | -11.53911922 |
| 101 | IKBKG | 11 | 384.9 | 0 |
| 102 | TERF1 | 10 | 1628.49 | 0 |
| 103 | MID2 | 10 | 1534.37 | -15.72362855 |
| 104 | L3MBTL1 | 10 | 1511.63 | -10.3180913 |

| | | | | |
|---|---|---|---|---|
| 105 | CRK | 10 | 1436.3 | 0 |
| 106 | NOS1 | 10 | 1409.8 | 17.85714286 |
| 107 | SAE1 | 10 | 1222.72 | -19.13422885 |
| 108 | HNF1A | 10 | 1184.5 | 0 |
| 109 | AMBP | 10 | 1042.72 | -20.23057429 |
| 110 | CCAR1 | 10 | 1029.58 | -10.18921065 |
| 111 | EGFR | 10 | 748.01 | 0 |
| 112 | HDAC1 | 10 | 665.78 | 0 |
| 113 | TAF8 | 10 | 408.11 | 10.96999406 |
| 114 | COPS6 | 10 | 320.69 | 0 |
| 115 | DDR1 | 9 | 1422.88 | 24.41290323 |
| 116 | POT1 | 9 | 1169.22 | 0 |
| 117 | SIRT6 | 9 | 1026.3 | -25.16339869 |
| 118 | GINS4 | 9 | 1015.21 | -20.5911083 |
| 119 | PPIE | 9 | 935.05 | 13.61707009 |
| 120 | NCOA4 | 9 | 905.8 | -11.32027902 |
| 121 | KCNH2 | 9 | 808.38 | 11.70187061 |
| 122 | SMAD2 | 9 | 596.67 | 0 |
| 123 | ILF3 | 9 | 589.33 | 0 |
| 124 | CDK2AP1 | 9 | 493.8 | -11.02325581 |
| 125 | UBD | 9 | 448.23 | 0 |
| 126 | F10 | 8 | 3153.89 | -13.17388003 |
| 127 | MATK | 8 | 1516.76 | -27.08355414 |
| 128 | PIH1D1 | 8 | 1361.59 | -20.13082734 |
| 129 | IBTK | 8 | 1359.57 | -10.80118972 |
| 130 | HOMER3 | 8 | 1335.1 | 10.61532606 |

| | | | | |
|---|---|---|---|---|
| 131 | MRPL15 | 8 | 1332.61 | 11.08810785 |
| 132 | VIM | 8 | 1025.24 | 0 |
| 133 | PIK3R1 | 8 | 553.07 | 0 |
| 134 | SRRM2 | 8 | 380.99 | 0 |
| 135 | UBE2D1 | 8 | 333.14 | 0 |
| 136 | AR | 8 | 278.68 | 0 |
| 137 | MDK | 8 | 224.34 | 18.33024119 |
| 138 | HNRNPM | 8 | 223.57 | 0 |
| 139 | CUL4B | 8 | 216.39 | 0 |
| 140 | MDM2 | 8 | 215.1 | 0 |
| 141 | NR2F2 | 7 | 1130.92 | 12.30783391 |
| 142 | CBL | 7 | 1068.57 | 0 |
| 143 | RGS3 | 7 | 873.05 | 14.64135021 |
| 144 | F2R | 7 | 857.33 | -10.04558482 |
| 145 | PHACTR4 | 7 | 803.49 | 14.90112994 |
| 146 | DYSF | 7 | 786.99 | -12.68664758 |
| 147 | GINS2 | 7 | 754.36 | -13.763999 |
| 148 | ATXN7L3 | 7 | 732.5 | 13.65551171 |
| 149 | YWHAG | 7 | 694.84 | 0 |
| 150 | TRAF2 | 7 | 615.02 | 0 |
| 151 | SUMO3 | 7 | 589.3 | 0 |
| 152 | PRKCSH | 7 | 581.72 | -21.01236017 |
| 153 | ACTB | 7 | 544.31 | 0 |
| 154 | CTNNB1 | 7 | 530.79 | 0 |
| 155 | FOS | 7 | 517.91 | 0 |
| 156 | SRPK2 | 7 | 487.24 | 0 |
| 157 | UBQLN4 | 7 | 424 | 0 |
| 158 | HSPA8 | 7 | 421.13 | 0 |
| 159 | EEF1A1 | 7 | 386.96 | 0 |

| | | | | |
|---|---|---|---|---|
| 160 | FOXH1 | 7 | 375.26 | -10.93239444 |
| 161 | VCP | 7 | 341.28 | 0 |
| 162 | AKT1 | 7 | 339.31 | 0 |
| 163 | STAT3 | 7 | 310.52 | 0 |
| 164 | RPL3 | 7 | 276.53 | 0 |
| 165 | PDCD6IP | 7 | 250.59 | 0 |
| 166 | CSNK2B | 7 | 227.98 | 0 |
| 167 | TMEM177 | 7 | 161.12 | 10.7813703 |
| 168 | IGBP1 | 7 | 150.13 | 0 |
| 169 | EIF4A3 | 7 | 41.66 | 0 |
| 170 | EXOC7 | 6 | 1478.4 | 14.00485374 |
| 171 | SSTR3 | 6 | 1124.27 | 13.69205298 |
| 172 | PPP4R2 | 6 | 953.08 | 14.40733332 |
| 173 | PAK2 | 6 | 898.28 | 0 |
| 174 | AP1M2 | 6 | 874.97 | 17.43690472 |
| 175 | CALM3 | 6 | 771.71 | 0 |
| 176 | TNFRSF10C | 6 | 681.73 | 16.42309636 |
| 177 | ARL4D | 6 | 637.28 | -12.23162088 |
| 178 | NR3C1 | 6 | 570.79 | 0 |
| 179 | CDC42 | 6 | 494.77 | 0 |
| 180 | ARL6IP4 | 6 | 469.72 | 12.77897455 |
| 181 | CDK1 | 6 | 430.89 | 0 |
| 182 | HNRNPU | 6 | 423.97 | 0 |
| 183 | CDKN1A | 6 | 398.58 | 0 |
| 184 | NCL | 6 | 357.5 | 0 |
| 185 | SNRPD2 | 6 | 344.53 | 0 |
| 186 | PCGF3 | 6 | 334.9 | 12.46448229 |
| 187 | GSK3B | 6 | 320.48 | 0 |
| 188 | HSPA1A | 6 | 302.53 | 0 |
| 189 | SRPK1 | 6 | 294.44 | 0 |
| 190 | LCK | 6 | 282.32 | 0 |

| | | | | |
|---|---|---|---|---|
| 191 | SIMC1 | 6 | 261.89 | -12.69516375 |
| 192 | VHL | 6 | 251.74 | 0 |
| 193 | NQO1 | 6 | 246.16 | 15.32276203 |
| 194 | SQSTM1 | 6 | 175.25 | 0 |
| 195 | ZNF689 | 6 | 161.7 | 10.64866543 |
| 196 | XRCC6 | 6 | 154.24 | 0 |
| 197 | DNAJA1 | 6 | 153.49 | 0 |
| 198 | CDKN2A | 6 | 147.86 | 0 |
| 199 | VPS26A | 6 | 144.31 | 0 |
| 200 | UBE2C | 6 | 116.54 | 0 |
| 201 | HIST1H3H | 6 | 111.91 | 0 |
| 202 | SAP18 | 6 | 111.37 | 0 |
| 203 | RB1 | 6 | 95.44 | 0 |
| 204 | NEDD8 | 6 | 82.63 | 0 |
| 205 | LNX1 | 5 | 1319.28 | 0 |
| 206 | ATP6V1G1 | 5 | 1274.25 | -14.97846937 |
| 207 | WDR73 | 5 | 1268.08 | 17.49580981 |
| 208 | PAX7 | 5 | 875.36 | -11.79847495 |
| 209 | PPP1CA | 5 | 867.36 | 0 |
| 210 | DBP | 5 | 853.36 | -18.88432179 |
| 211 | SIPA1L1 | 5 | 851.03 | -15.64637198 |
| 212 | ATXN1 | 5 | 806.13 | 0 |
| 213 | VAV2 | 5 | 739.58 | 0 |
| 214 | PLK4 | 5 | 727.84 | 19.9696697 |
| 215 | SETX | 5 | 716.24 | -11.80237604 |
| 216 | VPS51 | 5 | 635.08 | 18.47006129 |
| 217 | MYL2 | 5 | 606.7 | 11.13942483 |
| 218 | KAT2A | 5 | 596.52 | 0 |

| | | | | |
|---|---|---|---|---|
| 219 | MCCC2 | 5 | 563.68 | 10.53041743 |
| 220 | UBR7 | 5 | 523.68 | -11.11563381 |
| 221 | SERF2 | 5 | 494.32 | 10.78098472 |
| 222 | CEBPB | 5 | 474.99 | 0 |
| 223 | CMBL | 5 | 384.49 | 10.2980713 |
| 224 | PABPC1 | 5 | 378.31 | 0 |
| 225 | PRPSAP2 | 5 | 345.91 | -21.99777613 |
| 226 | SIRT1 | 5 | 328.8 | 0 |
| 227 | CDC5L | 5 | 305.07 | 0 |
| 228 | SNRNP70 | 5 | 285.82 | 0 |
| 229 | KPNA2 | 5 | 267.04 | 0 |
| 230 | EPB49 | 5 | 265.22 | -20.3026714 |
| 231 | EPS8L1 | 5 | 258.04 | -13.76208018 |
| 232 | PPP1CC | 5 | 257.96 | 0 |
| 233 | NFKB1 | 5 | 250.06 | 0 |
| 234 | EIF6 | 5 | 243.42 | 0 |
| 235 | AOX1 | 5 | 220.72 | -17.84657214 |
| 236 | DCAF4 | 5 | 218.38 | -11.40170693 |
| 237 | APOA1 | 5 | 214.64 | 0 |
| 238 | MAPK1 | 5 | 198.3 | 0 |
| 239 | RUSC1 | 5 | 195.3 | -18.98785425 |
| 240 | NCOA6 | 5 | 187.89 | 0 |
| 241 | SF3A1 | 5 | 178.37 | 0 |
| 242 | ETS1 | 5 | 155.66 | 0 |
| 243 | NDRG1 | 5 | 145.49 | 0 |
| 244 | MAPK6 | 5 | 144.79 | 0 |
| 245 | UBE2D3 | 5 | 144.45 | 0 |
| 246 | POLR2A | 5 | 141.49 | 0 |

| | | | | |
|---|---|---|---|---|
| 247 | HAS1 | 5 | 133.29 | -22.15357458 |
| 248 | RPL18A | 5 | 131.04 | 0 |
| 249 | PCK2 | 5 | 124.7 | 18.05555556 |
| 250 | UBE2D2 | 5 | 110.68 | 0 |
| 251 | PLCG1 | 5 | 110.03 | 0 |
| 252 | SFN | 5 | 109.1 | 0 |
| 253 | CUL2 | 5 | 103.49 | 0 |
| 254 | MCM7 | 5 | 102.38 | 0 |
| 255 | UPF1 | 5 | 98.98 | 0 |
| 256 | NPPB | 5 | 84.04 | 15.58047344 |
| 257 | SHC1 | 5 | 83.64 | 0 |
| 258 | CD2BP2 | 5 | 78.99 | 0 |
| 259 | YWHAE | 5 | 77.94 | 0 |
| 260 | PRPF19 | 5 | 77.68 | 0 |
| 261 | APOA5 | 5 | 70.59 | 15.3011658 |
| 262 | CELF3 | 5 | 69.72 | 22.17164279 |
| 263 | RPL5 | 5 | 54.06 | 0 |
| 264 | MDFI | 4 | 1244.06 | 0 |
| 265 | ABCD1 | 4 | 1185.35 | -11.45628411 |
| 266 | TINF2 | 4 | 851.11 | 0 |
| 267 | CREBBP | 4 | 777.6 | 0 |
| 268 | TJP1 | 4 | 754.95 | 0 |
| 269 | SH3GL2 | 4 | 750.43 | 0 |
| 270 | USHBP1 | 4 | 606.42 | 0 |
| 271 | ARHGAP27 | 4 | 546.59 | -13.74946375 |
| 272 | CHD3 | 4 | 467.89 | 0 |
| 273 | TRIM63 | 4 | 439.79 | 0 |
| 274 | RAD51C | 4 | 416.17 | -13.02061055 |

| | | | | |
|---|---|---|---|---|
| 275 | COASY | 4 | 408.87 | -19.27691347 |
| 276 | SERTAD1 | 4 | 397.25 | 0 |
| 277 | KRT73 | 4 | 393.93 | 15.59735114 |
| 278 | RAC1 | 4 | 386.86 | 0 |
| 279 | PCP4 | 4 | 377.3 | 25.13227513 |
| 280 | ISG20 | 4 | 361.3 | 26.67956769 |
| 281 | SPTBN1 | 4 | 335.27 | 0 |
| 282 | IRAK1 | 4 | 334.02 | 0 |
| 283 | USH1C | 4 | 333.41 | -15.09568525 |
| 284 | PNPO | 4 | 324.26 | 16.87888199 |
| 285 | KIR2DL4 | 4 | 318 | 13.07364836 |
| 286 | KPNB1 | 4 | 306.7 | 0 |
| 287 | HDAC4 | 4 | 288.64 | 0 |
| 288 | DCTN4 | 4 | 288.61 | 17.75735843 |
| 289 | FAS | 4 | 281.11 | 0 |
| 290 | CARHSP1 | 4 | 275.38 | 11.42265952 |
| 291 | HNRNPF | 4 | 233.89 | 0 |
| 292 | UCHL3 | 4 | 232.26 | 0 |
| 293 | ATP5A1 | 4 | 228.57 | 0 |
| 294 | MAN2A2 | 4 | 218.82 | 23.64103121 |
| 295 | RAP1A | 4 | 210.46 | 0 |
| 296 | CEBPA | 4 | 204.33 | 0 |
| 297 | DYRK1A | 4 | 196.04 | 0 |
| 298 | CTTN | 4 | 187.42 | 0 |
| 299 | TUBA8 | 4 | 178.75 | 10.36429324 |
| 300 | PDE4A | 4 | 170.26 | -18.65566534 |
| 301 | DYNLL1 | 4 | 165.69 | 0 |
| 302 | DLG4 | 4 | 163.57 | 0 |
| 303 | WAS | 4 | 161.37 | 0 |
| 304 | TUBA1A | 4 | 160.61 | 0 |

| | | | | |
|---|---|---|---|---|
| 305 | USP9X | 4 | 160.23 | 0 |
| 306 | ANXA7 | 4 | 156.59 | 0 |
| 307 | TBP | 4 | 156.27 | 0 |
| 308 | CYP2E1 | 4 | 146.81 | 17.71943624 |
| 309 | HIST3H3 | 4 | 141.66 | 0 |
| 310 | PMEPA1 | 4 | 137.81 | -31.01662309 |
| 311 | C14orf1 | 4 | 136.62 | 0 |
| 312 | PRMT5 | 4 | 132.46 | 0 |
| 313 | EPS15 | 4 | 132.22 | 0 |
| 314 | DSTN | 4 | 132.22 | 0 |
| 315 | TUBB | 4 | 121.22 | 0 |
| 316 | TRADD | 4 | 118.93 | 0 |
| 317 | NEDD4L | 4 | 118.54 | 0 |
| 318 | BIRC3 | 4 | 114.71 | 0 |
| 319 | A2M | 4 | 110.68 | 0 |
| 320 | ALB | 4 | 107.86 | 0 |
| 321 | CSF3 | 4 | 105.46 | -30.7312253 |
| 322 | PTPN6 | 4 | 102.62 | 0 |
| 323 | KAT5 | 4 | 100.34 | 0 |
| 324 | PTN | 4 | 96.22 | 0 |
| 325 | TERF2IP | 4 | 94.03 | 0 |
| 326 | ANPEP | 4 | 92.81 | -17.30523014 |
| 327 | VDR | 4 | 86.67 | 0 |
| 328 | UBE2E1 | 4 | 83.66 | 0 |
| 329 | TSR1 | 4 | 81.94 | 0 |
| 330 | UBE2E2 | 4 | 81.87 | 0 |
| 331 | PAICS | 4 | 78.19 | 0 |
| 332 | BARD1 | 4 | 69.17 | 0 |
| 333 | LYN | 4 | 68.93 | 0 |
| 334 | PA2G4 | 4 | 67.26 | 0 |

| | | | | |
|---|---|---|---|---|
| 335 | PARK2 | 4 | 54.47 | 0 |
| 336 | SNRPB | 4 | 51.21 | 0 |
| 337 | GAPDH | 4 | 46.32 | 0 |
| 338 | NCK1 | 4 | 41.55 | 0 |
| 339 | HDAC11 | 4 | 33.84 | 0 |
| 340 | SNRPA1 | 4 | 31.73 | 0 |
| 341 | CAV1 | 4 | 25.56 | 0 |
| 342 | PHB | 3 | 2155.43 | 0 |
| 343 | PDLIM4 | 3 | 934.03 | 12.58184713 |
| 344 | VKORC1L1 | 3 | 879.41 | 12.57696127 |
| 345 | CEBPD | 3 | 734.72 | 0 |
| 346 | CDK5RAP3 | 3 | 726.39 | 0 |
| 347 | CCNA1 | 3 | 723.84 | 0 |
| 348 | PITX3 | 3 | 721.55 | 0 |
| 349 | PSMA1 | 3 | 664.09 | 0 |
| 350 | NFYC | 3 | 562.25 | 0 |
| 351 | ACTG1 | 3 | 378.63 | 0 |
| 352 | KRT83 | 3 | 307.1 | -17.00700701 |
| 353 | MT1A | 3 | 285.52 | -15.95382168 |
| 354 | FASLG | 3 | 279.88 | 0 |
| 355 | MAEA | 3 | 260.22 | 11.85714286 |
| 356 | CTSZ | 3 | 256.47 | 12.42827198 |
| 357 | PCBP1 | 3 | 244.35 | 0 |
| 358 | HIF1A | 3 | 233.68 | 0 |
| 359 | PEX5 | 3 | 232.76 | 0 |
| 360 | FGD1 | 3 | 224.66 | 13.42414861 |
| 361 | PPARA | 3 | 182.11 | 0 |
| 362 | IKBKB | 3 | 175.61 | 0 |
| 363 | KCNAB3 | 3 | 174.83 | 10.49174661 |
| 364 | GFI1B | 3 | 167.23 | 0 |
| 365 | JUN | 3 | 157.82 | 0 |

| | | | | |
|---|---|---|---|---|
| 366 | USF1 | 3 | 155.97 | 0 |
| 367 | NIPSNAP1 | 3 | 148.79 | -12.78946721 |
| 368 | ACD | 3 | 148.07 | 0 |
| 369 | ONECUT1 | 3 | 140.97 | 0 |
| 370 | HTR2A | 3 | 139.58 | -10.23484608 |
| 371 | APOE | 3 | 138.36 | 0 |
| 372 | NR1H4 | 3 | 132.46 | 0 |
| 373 | ADCY7 | 3 | 129.99 | 22.55754476 |
| 374 | UBA52 | 3 | 129.76 | 0 |
| 375 | ZKSCAN5 | 3 | 127.15 | -22.36495829 |
| 376 | DNM2 | 3 | 114.32 | 0 |
| 377 | TUBGCP4 | 3 | 106.81 | 0 |
| 378 | WDTC1 | 3 | 105.47 | 13.3971831 |
| 379 | PLAT | 3 | 100.03 | 0 |
| 380 | ATL3 | 3 | 96.94 | -11.56622611 |
| 381 | WASL | 3 | 96.62 | 0 |
| 382 | TUBA3D | 3 | 92.18 | 0 |
| 383 | ZBTB16 | 3 | 92.04 | 0 |
| 384 | DTNBP1 | 3 | 90.34 | 0 |
| 385 | TCF21 | 3 | 87.78 | -14.31752365 |
| 386 | UBE2E3 | 3 | 87.61 | 0 |
| 387 | CREB1 | 3 | 87.39 | 0 |
| 388 | MYD88 | 3 | 86.41 | 0 |
| 389 | HDAC6 | 3 | 85.9 | 0 |
| 390 | TAF1 | 3 | 83.11 | 0 |
| 391 | POM121 | 3 | 81.98 | 0 |
| 392 | TCF12 | 3 | 76.27 | 0 |
| 393 | SCRIB | 3 | 72.9 | 0 |

| | | | | |
|---|---|---|---|---|
| 394 | TLE1 | 3 | 71.55 | 0 |
| 395 | DDX1 | 3 | 71.13 | 0 |
| 396 | STAM2 | 3 | 70.75 | 0 |
| 397 | RPL22L1 | 3 | 70.52 | -18.77380205 |
| 398 | TAF7 | 3 | 66.36 | 0 |
| 399 | XPO1 | 3 | 65.95 | 0 |
| 400 | ESR2 | 3 | 65.79 | 0 |
| 401 | MIIP | 3 | 63.54 | 23.46419997 |
| 402 | APEH | 3 | 62.42 | 0 |
| 403 | TNFRSF1A | 3 | 62.32 | 0 |
| 404 | MAP3K3 | 3 | 58.88 | 0 |
| 405 | G2E3 | 3 | 56.48 | 12.05819958 |
| 406 | HERC6 | 3 | 56.48 | -22.48307525 |
| 407 | THOC5 | 3 | 56.2 | 0 |
| 408 | SPOCD1 | 3 | 55.08 | -12.60432378 |
| 409 | EWSR1 | 3 | 54.37 | 0 |
| 410 | NFKBIB | 3 | 54.27 | 0 |
| 411 | SRF | 3 | 54.22 | 0 |
| 412 | TSC22D4 | 3 | 52.47 | 0 |
| 413 | ACTA2 | 3 | 52.12 | 0 |
| 414 | ZFYVE19 | 3 | 51.42 | 0 |
| 415 | SRSF3 | 3 | 50.36 | 0 |
| 416 | MLH1 | 3 | 49.03 | 0 |
| 417 | FLOT1 | 3 | 48.54 | 0 |
| 418 | UBE2K | 3 | 48.17 | 0 |
| 419 | UBE2B | 3 | 46.78 | 0 |
| 420 | TAF9 | 3 | 46.17 | 0 |
| 421 | GPR39 | 3 | 45.05 | 18.21648984 |
| 422 | UBE2H | 3 | 44.09 | 0 |
| 423 | HOXA9 | 3 | 43.82 | 0 |

| | | | | |
|---|---|---|---|---|
| 424 | BIRC2 | 3 | 43.4 | 0 |
| 425 | TAF10 | 3 | 42.59 | 0 |
| 426 | DHX30 | 3 | 39.93 | 0 |
| 427 | USP7 | 3 | 39.76 | 0 |
| 428 | UBXN1 | 3 | 39.61 | 0 |
| 429 | SNAPIN | 3 | 39.41 | 0 |
| 430 | ZNF408 | 3 | 39.17 | 0 |
| 431 | TTR | 3 | 38.99 | 0 |
| 432 | MYO15A | 3 | 38.95 | 11.24751826 |
| 433 | SNCA | 3 | 36.99 | 0 |
| 434 | MED1 | 3 | 36.52 | 0 |
| 435 | TYSND1 | 3 | 34.79 | 11.1003409 |
| 436 | XIAP | 3 | 31.47 | 0 |
| 437 | CIAO1 | 3 | 31.1 | 0 |
| 438 | XPO7 | 3 | 30.88 | 0 |
| 439 | ATP6V1B2 | 3 | 29.95 | 0 |
| 440 | GNA11 | 3 | 27.82 | 0 |
| 441 | GOLGA2 | 3 | 27.62 | 0 |
| 442 | MYL12A | 3 | 27.55 | 0 |
| 443 | TRPC3 | 3 | 27.35 | -10.14567874 |
| 444 | HAMP | 3 | 27.01 | 28.14538677 |
| 445 | CRH | 3 | 26.79 | 10.87427493 |
| 446 | MLL | 3 | 24.54 | 0 |
| 447 | CRMP1 | 3 | 24 | 0 |
| 448 | COL5A3 | 3 | 22.61 | 16.65572874 |
| 449 | UBA2 | 3 | 21.82 | 0 |
| 450 | PSEN1 | 3 | 21.51 | 0 |
| 451 | IQGAP1 | 3 | 21.28 | 0 |
| 452 | NFKBIA | 3 | 21.03 | 0 |
| 453 | IQSEC1 | 3 | 20.55 | -15.25651229 |

| | | | | |
|---|---|---|---|---|
| 454 | CDH1 | 3 | 19.53 | 0 |
| 455 | TSC22D1 | 3 | 19.01 | 0 |
| 456 | ERBB2 | 3 | 18.55 | 0 |
| 457 | ARF6 | 3 | 18.52 | 0 |
| 458 | GNB2L1 | 3 | 16.91 | 0 |
| 459 | FAM131B | 3 | 16.38 | -14.52636895 |
| 460 | NFKB2 | 3 | 14.78 | 0 |
| 461 | COPS2 | 3 | 14.56 | 0 |
| 462 | GNAI1 | 3 | 11.56 | 0 |
| 463 | PLTP | 3 | 4.85 | -21.9858156 |
| 464 | RNPS1 | 3 | 3.09 | 0 |
| 465 | GINS1 | 3 | 0 | -16.24493927 |
| 466 | DPP6 | 2 | 1424 | -13.39739856 |
| 467 | PPP1R16A | 2 | 715.25 | 0 |
| 468 | NAA38 | 2 | 713 | 0 |
| 469 | KCND2 | 2 | 713 | 0 |
| 470 | SPP1 | 2 | 713 | 0 |
| 471 | RPA2 | 2 | 713 | 0 |
| 472 | VEGFA | 2 | 713 | 0 |
| 473 | CKAP4 | 2 | 434.88 | 0 |
| 474 | VAMP2 | 2 | 376.73 | 0 |
| 475 | USP13 | 2 | 360.76 | 0 |
| 476 | SCP2 | 2 | 306.92 | 0 |
| 477 | MT1F | 2 | 302.81 | 17.07041344 |
| 478 | SOS1 | 2 | 292.99 | 0 |
| 479 | IRF7 | 2 | 281.26 | 0 |
| 480 | WDR61 | 2 | 270.77 | 0 |
| 481 | ITPR3 | 2 | 267.8 | 0 |
| 482 | ACOX1 | 2 | 256.05 | 0 |
| 483 | SLC10A2 | 2 | 223.77 | 19.11392405 |

| | | | | |
|---|---|---|---|---|
| 484 | TMCO7 | 2 | 216.34 | -24.19205701 |
| 485 | RND3 | 2 | 212.37 | 0 |
| 486 | MRPS17 | 2 | 184.23 | -15.89559927 |
| 487 | GATA4 | 2 | 173.97 | 0 |
| 488 | UBA1 | 2 | 170.92 | 0 |
| 489 | CD3E | 2 | 167.88 | 0 |
| 490 | PNMT | 2 | 167.2 | 14.99975986 |
| 491 | FGD3 | 2 | 151.81 | -27.32594937 |
| 492 | EPS8 | 2 | 147.19 | 0 |
| 493 | RBPMS | 2 | 131.62 | 0 |
| 494 | PALM | 2 | 126.8 | 11.65543558 |
| 495 | VKORC1 | 2 | 126.15 | 0 |
| 496 | RHOXF2 | 2 | 123.47 | 0 |
| 497 | HSD3B7 | 2 | 120.05 | 14.91576912 |
| 498 | RANBP17 | 2 | 120.05 | 10.46476762 |
| 499 | ABI1 | 2 | 115.87 | 0 |
| 500 | PSMC5 | 2 | 103.8 | 0 |
| 501 | USF2 | 2 | 103.13 | 0 |
| 502 | IQCB1 | 2 | 101.25 | 0 |
| 503 | PAX6 | 2 | 97.08 | 0 |
| 504 | GIT1 | 2 | 96.89 | 0 |
| 505 | TRPC4 | 2 | 90.85 | 0 |
| 506 | MZB1 | 2 | 88.99 | 14.09249831 |
| 507 | TRIM28 | 2 | 83.34 | 0 |
| 508 | FAIM2 | 2 | 81.99 | 15.48509588 |
| 509 | NFYB | 2 | 81.42 | 0 |
| 510 | TAF6 | 2 | 81.25 | 0 |
| 511 | TMEM102 | 2 | 80.16 | 13.90491422 |
| 512 | NMNAT1 | 2 | 80.03 | 0 |
| 513 | DDIT3 | 2 | 76.92 | 0 |

| | | | | |
|---|---|---|---|---|
| 514 | SPINT2 | 2 | 72.78 | -12.57339571 |
| 515 | 11-Sep | 2 | 72.47 | 0 |
| 516 | VAMP1 | 2 | 72.09 | 12.39162089 |
| 517 | TTC38 | 2 | 72.09 | 14.57203134 |
| 518 | ZNF586 | 2 | 72.09 | 14.64212077 |
| 519 | CWF19L1 | 2 | 72.09 | -17.88451309 |
| 520 | TPM4 | 2 | 69.4 | 0 |
| 521 | RGMA | 2 | 67.92 | 24.28750772 |
| 522 | GNB1 | 2 | 64.61 | 0 |
| 523 | NFATC3 | 2 | 60.46 | 0 |
| 524 | SLC25A3 | 2 | 59.18 | 0 |
| 525 | DHRS3 | 2 | 56.7 | 11.88140956 |
| 526 | TCTN2 | 2 | 56.7 | -19.74948079 |
| 527 | ANGEL2 | 2 | 56.7 | -10.22966507 |
| 528 | ATXN7 | 2 | 55 | 0 |
| 529 | ZNF44 | 2 | 53.98 | 21.20105498 |
| 530 | GJB5 | 2 | 53.89 | 0 |
| 531 | YBX1 | 2 | 53.63 | 0 |
| 532 | STAM | 2 | 52.77 | 0 |
| 533 | ITPRIPL1 | 2 | 52.72 | -12.72555503 |
| 534 | CYB5R1 | 2 | 52.72 | -10.71956022 |
| 535 | GK | 2 | 48.49 | 0 |
| 536 | ASAP1 | 2 | 47.46 | 0 |
| 537 | ERBB2IP | 2 | 44.68 | 0 |
| 538 | CTSB | 2 | 44.06 | 0 |
| 539 | ZNF385A | 2 | 41.81 | -15.13931889 |
| 540 | RARA | 2 | 40.84 | 0 |
| 541 | SMAD7 | 2 | 40.24 | 0 |

| | | | | |
|---|---|---|---|---|
| 542 | S100A9 | 2 | 39.8 | 0 |
| 543 | TUBB2B | 2 | 39.49 | 0 |
| 544 | ENO3 | 2 | 38.24 | 0 |
| 545 | PACSIN2 | 2 | 37.25 | 0 |
| 546 | SORD | 2 | 36.92 | 0 |
| 547 | FAM90A1 | 2 | 33.53 | 19.62301587 |
| 548 | NUDT7 | 2 | 33.21 | -13.02141011 |
| 549 | CBX5 | 2 | 33.19 | 0 |
| 550 | UBB | 2 | 31.94 | 0 |
| 551 | ZFYVE28 | 2 | 31.94 | 11.95305224 |
| 552 | PRKD2 | 2 | 31.89 | 0 |
| 553 | ACER3 | 2 | 31.81 | 12.32267585 |
| 554 | KAT7 | 2 | 31.68 | 0 |
| 555 | SMARCAD1 | 2 | 31.2 | 0 |
| 556 | TMEM101 | 2 | 31.09 | -19.31227782 |
| 557 | UBE2O | 2 | 30.76 | 0 |
| 558 | APEX1 | 2 | 30.69 | 0 |
| 559 | RGS9 | 2 | 30.33 | 14.68599225 |
| 560 | ABCA1 | 2 | 30.23 | 0 |
| 561 | TNNT1 | 2 | 30.21 | 0 |
| 562 | CRKL | 2 | 27.95 | 0 |
| 563 | CACNA1A | 2 | 27.26 | 0 |
| 564 | PLA2G4A | 2 | 27.01 | 0 |
| 565 | DMKN | 2 | 26.83 | -14.03125067 |
| 566 | CARS | 2 | 26.73 | 0 |
| 567 | HSD17B14 | 2 | 26.07 | 12.72055505 |
| 568 | ZMYM1 | 2 | 25.96 | 14.52109141 |
| 569 | LRSAM1 | 2 | 25.89 | 0 |
| 570 | STAT5A | 2 | 25.73 | 0 |
| 571 | SH3GL1 | 2 | 24.28 | 0 |

| | | | | |
|---|---|---|---|---|
| 572 | DYNLL2 | 2 | 24.16 | 0 |
| 573 | NLK | 2 | 23.68 | 0 |
| 574 | NFE2 | 2 | 23.21 | 0 |
| 575 | LSM4 | 2 | 23.2 | 0 |
| 576 | MED8 | 2 | 22.46 | 0 |
| 577 | CD320 | 2 | 22.43 | 0 |
| 578 | PLEKHG5 | 2 | 21.25 | 10.63829787 |
| 579 | FUCA2 | 2 | 21.23 | 11.09652771 |
| 580 | TRIM29 | 2 | 21.2 | 0 |
| 581 | TOLLIP | 2 | 20.98 | 0 |
| 582 | RCHY1 | 2 | 20.77 | 0 |
| 583 | CPSF3 | 2 | 20.31 | 0 |
| 584 | USP6 | 2 | 19.77 | 0 |
| 585 | CEBPE | 2 | 19.41 | 0 |
| 586 | PYGL | 2 | 19.11 | 0 |
| 587 | PSPC1 | 2 | 19.11 | 0 |
| 588 | CTSD | 2 | 18.95 | 0 |
| 589 | SIRT3 | 2 | 18.53 | 0 |
| 590 | SUPT5H | 2 | 18.13 | 0 |
| 591 | QKI | 2 | 17.42 | 0 |
| 592 | BCL2L11 | 2 | 17.2 | 0 |
| 593 | NEK3 | 2 | 16.37 | 11.21173141 |
| 594 | SNX2 | 2 | 16.14 | 0 |
| 595 | ARHGEF7 | 2 | 16.05 | 0 |
| 596 | TRIP11 | 2 | 15.79 | 0 |
| 597 | PTBP1 | 2 | 15.76 | 0 |
| 598 | GRAP2 | 2 | 15.6 | 0 |
| 599 | NCK2 | 2 | 15.17 | 0 |
| 600 | MPDZ | 2 | 15.1 | 0 |
| 601 | FANCD2 | 2 | 14.59 | 0 |
| 602 | BTK | 2 | 14.53 | 0 |
| 603 | MYCN | 2 | 14.5 | 0 |

| | | | | |
|---|---|---|---|---|
| 604 | CDK2AP2 | 2 | 13.24 | 0 |
| 605 | BRF2 | 2 | 13.2 | 0 |
| 606 | ITK | 2 | 13.1 | 0 |
| 607 | ZNF143 | 2 | 12.72 | 0 |
| 608 | CBLB | 2 | 12.53 | 0 |
| 609 | ATXN2 | 2 | 11.98 | 0 |
| 610 | PTPN11 | 2 | 11.79 | 0 |
| 611 | PROCR | 2 | 10.81 | 0 |
| 612 | KRT38 | 2 | 10.72 | 18.20971867 |
| 613 | PLAUR | 2 | 10.61 | 0 |
| 614 | TFEB | 2 | 10.57 | 10.3831553 |
| 615 | DNAJC4 | 2 | 10.52 | -18.48648649 |
| 616 | AKR1B1 | 2 | 10.36 | 0 |
| 617 | CCDC85B | 2 | 10.16 | 0 |
| 618 | RAF1 | 2 | 10 | 0 |
| 619 | BEGAIN | 2 | 9.8 | 0 |
| 620 | POLA1 | 2 | 9.33 | 0 |
| 621 | POLR1D | 2 | 8.66 | 0 |
| 622 | TCF3 | 2 | 7.61 | 0 |
| 623 | PINX1 | 2 | 7.48 | 0 |
| 624 | MCM4 | 2 | 7.2 | 0 |
| 625 | ATP6V1E1 | 2 | 7.05 | 0 |
| 626 | CEP76 | 2 | 6.81 | 0 |
| 627 | CHEK2 | 2 | 6.56 | 0 |
| 628 | UBE2L3 | 2 | 6.17 | 0 |
| 629 | NDE1 | 2 | 5.91 | -10.83774474 |
| 630 | RAD51 | 2 | 5.5 | 0 |
| 631 | PUF60 | 2 | 4.99 | 0 |
| 632 | ETF1 | 2 | 4.65 | 0 |
| 633 | TRIM55 | 2 | 4.63 | 0 |

| | | | | |
|---|---|---|---|---|
| 634 | AHSA1 | 2 | 4.45 | 0 |
| 635 | PTP4A3 | 2 | 3.96 | 0 |
| 636 | FKBP8 | 2 | 3.03 | 0 |
| 637 | ARHGEF25 | 2 | 2.5 | -18.98496241 |
| 638 | AP2B1 | 2 | 2.24 | 0 |
| 639 | UBE2I | 2 | 2.19 | 0 |
| 640 | SLFN12L | 2 | 1.96 | -17.45401338 |
| 641 | RPS6KL1 | 2 | 0.3 | -15.46579445 |
| 642 | TBR1 | 2 | 0 | -13.0460917 |
| 643 | FABP6 | 2 | 0 | 14.57521178 |
| 644 | SLC12A6 | 2 | 0 | -19.50497788 |
| 645 | ZNF707 | 1 | 0 | -10.50522846 |
| 646 | WNT5B | 1 | 0 | -14.11333659 |
| 647 | PGF | 1 | 0 | 17.06009966 |
| 648 | SPRYD4 | 1 | 0 | 15.09637188 |
| 649 | P2RX1 | 1 | 0 | -11.26171279 |
| 650 | SEMA4B | 1 | 0 | 14.38911013 |
| 651 | UCKL1 | 1 | 0 | -11.80669064 |
| 652 | PBX4 | 1 | 0 | -18.83535088 |
| 653 | IGSF22 | 1 | 0 | 11.53846154 |
| 654 | SLC7A8 | 1 | 0 | -40.74363992 |
| 655 | AQP2 | 1 | 0 | -21.13402062 |
| 656 | RCCD1 | 1 | 0 | -19.09976674 |
| 657 | TOP1MT | 1 | 0 | 15.78471484 |

| | | | | |
|---|---|---|---|---|
| 658 | NRGN | 1 | 0 | -19.32741357 |
| 659 | IBA57 | 1 | 0 | 11.57785584 |
| 660 | OSBPL1A | 1 | 0 | 10.37672439 |
| 661 | LRRC6 | 1 | 0 | -16.12932196 |
| 662 | TMEM176B | 1 | 0 | -13.26311973 |
| 663 | ENPEP | 1 | 0 | 15.28656529 |
| 664 | SLC17A9 | 1 | 0 | 18.99396593 |
| 665 | PLIN4 | 1 | 0 | -10.44036895 |
| 666 | SUSD3 | 1 | 0 | 13.07512479 |
| 667 | KCNIP2 | 1 | 0 | -12.92603632 |
| 668 | FGFBP2 | 1 | 0 | -17.30638201 |
| 669 | SYCP2L | 1 | 0 | -15.29411765 |
| 670 | TBC1D10C | 1 | 0 | 20.51867862 |
| 671 | PRSS33 | 1 | 0 | -11.21960356 |
| 672 | PLBD2 | 1 | 0 | -17.45553827 |
| 673 | IL28RA | 1 | 0 | -13.28182049 |
| 674 | SLC51A | 1 | 0 | 14.35946769 |
| 675 | SPA17 | 1 | 0 | 14.3130227 |
| 676 | PCDHA5 | 1 | 0 | -18.60317124 |
| 677 | GJD3 | 1 | 0 | 10.64665127 |
| 678 | C6orf170 | 1 | 0 | -41.28076063 |
| 679 | CDH4 | 1 | 0 | 10.00650699 |
| 680 | KLHDC9 | 1 | 0 | -10.41534165 |

| | | | | |
|---|---|---|---|---|
| 681 | ALS2CL | 1 | 0 | 10.70251436 |
| 682 | SLC16A3 | 1 | 0 | 13.40507788 |
| 683 | TOR4A | 1 | 0 | -10.1031555 |
| 684 | SLC6A8 | 1 | 0 | 11.05676882 |
| 685 | HOXB3 | 1 | 0 | -14.3977591 |
| 686 | SYCP1 | 1 | 0 | 14.50739668 |
| 687 | SLC35A1 | 1 | 0 | 12.37947316 |
| 688 | FAM102A | 1 | 0 | -16.0597161 |
| 689 | ZSWIM3 | 1 | 0 | 10.1334111 |
| 690 | AP1S3 | 1 | 0 | -10.95702069 |
| 691 | COLEC11 | 1 | 0 | 14.12857674 |
| 692 | REM2 | 1 | 0 | -25.01960784 |
| 693 | GAS2L2 | 1 | 0 | 17.50334033 |
| 694 | ACSL5 | 1 | 0 | -16.80434783 |
| 695 | PXMP4 | 1 | 0 | -10.51601631 |
| 696 | ZNF551 | 1 | 0 | 17.70154374 |
| 697 | NUDT22 | 1 | 0 | -11.98311417 |
| 698 | CLEC11A | 1 | 0 | -24.72385967 |
| 699 | GRTP1 | 1 | 0 | -11.84194818 |
| 700 | SLC2A10 | 1 | 0 | -17.92462066 |
| 701 | ZNF597 | 1 | 0 | 10.88286327 |
| 702 | NUP210L | 1 | 0 | -14.65801887 |
| 703 | ZNF354C | 1 | 0 | -17.16573796 |
| 704 | MYO7B | 1 | 0 | -12.89716112 |

| | | | | |
|---|---|---|---|---|
| 705 | LDLRAD4 | 1 | 0 | -10.46176976 |
| 706 | GSTO2 | 1 | 0 | 10.83863537 |
| 707 | ASAH2B | 1 | 0 | 15.99460916 |
| 708 | CTC1 | 1 | 0 | -17.32804233 |
| 709 | SERHL2 | 1 | 0 | -11.04450096 |
| 710 | C2orf50 | 1 | 0 | -10.14194139 |
| 711 | C2orf68 | 1 | 0 | -18.20188872 |
| 712 | IQCA1 | 1 | 0 | -15.98734108 |
| 713 | DNAAF3 | 1 | 0 | -14.74512147 |
| 714 | RHBDF1 | 1 | 0 | 10.29470688 |
| 715 | ACRBP | 1 | 0 | 10.31926174 |

**Supplementary Table 7.** Node characteristics of the minimum PPI network (**Figure 4A**) constructed based on the genes associated with differentially methylated promoters between non-diseased aortic and mitral valve tissue. Each node represents a gene. Degree refers to the number of connections/edges/PPIs that a node has to other nodes and betweenness centrality reflects the number of shortest paths passing through a node (2).

**2.8 Table S8. List of enriched pathways in the network constructed upon the genes associated with the differentially methylated hyo- and hypermethylated promoters between the non-diseased aortic and mitral valve tissue based on the Reactome pathway database (selected results)**

| **Reactome Pathway** | **Total** | **Expected** | **Hits** | **P.Value** | **FDR** | **Seed genes** | **Other genes** |
|---|---|---|---|---|---|---|---|
| Immune System | 1140 | 67.8 | 125 | 2.73E-13 | 3.82E-10 | CD44, AP1M2, ZAP70, TAB1, LIMK1, LCP2, SH3KBP1, ISG20, FGR, DCTN4, OSBPL1A, AP1S3, KIR2DL4, ADCY7, UBOX5, PRKCSH | ZBTB16, TRAF6, YWHAZ, DYNLL1, POM121, AP2B1, GRAP2, PSMA1, NFKBIB, IKBKG, MAP3K3, NFKB2, CDH1, MDM2, CREBBP, RELA, NFKBIA, EIF4A3, ACTB, STAT5A, STAT3, WAS, CBLB, RAF1, CRK, CTSB, ERBB2, EGFR, HSP90AA1, SQSTM1, MYD88, TOLLIP, PIK3R1, CDK1, FOS, NFKB1, AKT1, GRB2, NCK1, SRC, MAPK1, CDKN1A, APP, UBE2H, UBE2E2, UBE2K, UBE2L3, UBE2E1, UBE2E3, UBE2B, EP300, PTPN6, PARK2, ABI1, IKBKB, EGR1, CBL, SH3GL2, SOS1, RAC1, CDC42, CUL1, SUMO1, TUBA1A, GSK3B, UBE2D3, UBE2D2, WASL, CRKL, RCHY1, CUL3, CUL2, NEDD4L, SHC1, UBE2C, UBA1, PAK2, UBE2D1, UBA52, LCK, HSP90AB1, |

| | | | | | | | |
|---|---|---|---|---|---|---|---|
| | | | | | | | LRSAM1, TRAF2, TUBB2B, VAV2, RAP1A, LYN, ITPR3, TRIM63, JUN, CREB1, CD3E, PLCG1, ACTG1, PSMC5, KPNB1, KPNA2, VHL, BIRC2, ITK, BTK, PTPN11, IRAK1, BIRC3, IRF7, DNM2, CTSD, DYNLL2, UBE2O |
| Signaling by Interleukins | 116 | 6.91 | 29 | 1.68E-11 | 7.86E-09 | TAB1 | TRAF6, YWHAZ, IKBKG, MAP3K3, STAT5A, STAT3, RAF1, CRK, SQSTM1, MYD88, TOLLIP, PIK3R1, CDK1, NFKB1, GRB2, MAPK1, PTPN6, IKBKB, CBL, SOS1, CUL1, CRKL, SHC1, UBA52, LCK, LYN, PTPN11, IRAK1 |
| Developmental Biology | 417 | 24.9 | 55 | 1.05E-08 | 1.05E-06 | RXRA, ABLIM1, SMAD3, LIMK1, TRPC3, FOXH1, COL5A3, CDH4, RGMA | CRMP1, AP2B1, CREBBP, ACTB, SMAD2, RAF1, ERBB2, EGFR, HSP90AA1, MED1, CTNNB1, CDK1, TCF12, AKT1, GRB2, NCK1, SRC, MAPK1, HNF4A, EP300, SPTBN1, DLG4, SH3GL2, TRPC4, SOS1, RAC1, CDC42, TUBA1A, GSK3B, WASL, ONECUT1, PAK2, HSP90AB1, MED8, TUBB2B, CSNK2B, VAV2, CEBPB, TCF3, CREB1, PLCG1, |

| | | | | | | | |
|---|---|---|---|---|---|---|---|
| | | | | | | | CEBPD, ACTG1, NCK2, PTPN11, DNM2 |
| Apoptosis | 158 | 9.42 | 28 | 1.42E-07 | 8.66E-06 | TJP2, DNM1L, CASP7 | VIM, YWHAQ, YWHAZ, YWHAG, DYNLL1, PSMA1, TNFRSF1A, TRADD, CDH1, CTNNB1, AKT1, YWHAE, TJP1, BCL2L11, PAK2, UBA52, TRAF2, SFN, FASLG, FAS, PSMC5, KPNB1, BIRC2, XIAP, DYNLL2 |
| Signaling by TGF-beta Receptor Complex | 70 | 4.17 | 17 | 4.55E-07 | 2.06E-05 | RBL1, SMAD3, PMEPA1, E2F4 | MYC, SMAD2, PPP1CA, SMAD7, SP1, UBE2D3, XPO1, NEDD4L, HDAC1, PPP1CC, UBE2D1, UBA52, USP9X |
| Signaling by constitutively active EGFR | 19 | 1.13 | 9 | 4.70E-07 | 2.06E-05 | NA | EGFR, HSP90AA1, PIK3R1, GRB2, CBL, SOS1, SHC1, UBA52, PLCG1 |
| Transcriptional activity of SMAD2/SMAD3:SMAD4 heterotrimer | 43 | 2.56 | 13 | 6.99E-07 | 2.72E-05 | RBL1, SMAD3, E2F4 | MYC, SMAD2, SMAD7, SP1, UBE2D3, NEDD4L, HDAC1, UBE2D1, UBA52, USP9X |

| | | | | | | | |
|---|---|---|---|---|---|---|---|
| Cellular responses to stress | 27 | 1.61 | 10 | 1.71E-06 | 5.09E-05 | NA | CREBBP, HIF1A, EP300, UBE2D3, UBE2D2, CUL2, UBE2D1, UBA52, VHL, VEGFA |
| Cellular response to hypoxia | 27 | 1.61 | 10 | 1.71E-06 | 5.09E-05 | NA | CREBBP, HIF1A, EP300, UBE2D3, UBE2D2, CUL2, UBE2D1, UBA52, VHL, VEGFA |
| Regulation of Hypoxia-inducible Factor (HIF) by Oxygen | 27 | 1.61 | 10 | 1.71E-06 | 5.09E-05 | NA | CR+A7:H14EBBP, HIF1A, EP300, UBE2D3, UBE2D2, CUL2, UBE2D1, UBA52, VHL, VEGFA |
| Interleukin-3, 5 and GM-CSF signaling | 51 | 3.04 | 13 | 5.87E-06 | 0.000151 | NA | YWHAZ, STAT5A, CRK, PIK3R1, GRB2, PTPN6, CBL, SOS1, CRKL, SHC1, UBA52, LYN, PTPN11 |
| Signaling by PDGF | 189 | 11.3 | 28 | 5.93E-06 | 0.000151 | COL5A3, ADCY7 | MDM2, STAT5A, STAT3, RAF1, CRK, ERBB2, EGFR, PIK3R1, CDK1, AKT1, GRB2, NCK1, SRC, MAPK1, CDKN1A, PLAT, SOS1, GSK3B, CRKL, SPP1, LCK, ITPR3, CREB1, PLCG1, NCK2, PTPN11 |
| Interleukin-1 signaling | 45 | 2.68 | 12 | 8.12E-06 | 0.000187 | TAB1 | TRAF6, IKBKG, MAP3K3, SQSTM1, MYD88, TOLLIP, NFKB1, IKBKB, CUL1, UBA52, IRAK1 |

| | | | | | | | |
|---|---|---|---|---|---|---|---|
| mRNA Splicing | 115 | 6.85 | 20 | 1.21E-05 | 0.000249 | LSM2, MAGOH, PRPF6, CCAR1 | CD2BP2, RNPS1, SNRPB, SNRPA1, CPSF3, HNRNPM, SNRPD2, PCBP1, SF3A1, POLR2A, HNRNPF, HNRNPU, PTBP1, SRSF3, YBX1, SNRNP70 |
| Downregulation of TGF-beta receptor signaling | 27 | 1.61 | 9 | 1.55E-05 | 0.000316 | SMAD3, PMEPA1 | SMAD2, PPP1CA, SMAD7, XPO1, NEDD4L, PPP1CC, UBA52 |
| NOTCH1 Intracellular Domain Regulates Transcription | 50 | 2.98 | 12 | 2.61E-05 | 0.000515 | NA | TLE1, CREBBP, MYC, HIF1A, EP300, HDAC4, KAT2A, CUL1, HDAC6, HDAC1, UBA52, HDAC11 |
| Downregulation of SMAD2/3:SMAD4 transcriptional activity | 23 | 1.37 | 8 | 3.29E-05 | 0.000624 | SMAD3 | SMAD2, UBE2D3, NEDD4L, HDAC1, UBE2D1, UBA52, USP9X |
| TGF-beta receptor signaling activates SMADs | 30 | 1.79 | 9 | 4.04E-05 | 0.000736 | SMAD3, PMEPA1 | SMAD2, PPP1CA, SMAD7, XPO1, NEDD4L, PPP1CC, UBA52 |
| mRNA Processing | 140 | 8.35 | 21 | 7.32E-05 | 0.00125 | LSM2, MAGOH, PRPF6, CCAR1 | CD2BP2, RNPS1, SNRPB, SNRPA1, CPSF3, HNRNPM, SNRPD2, PCBP1, SF3A1, POLR2A, HNRNPF, HNRNPU, PTBP1, SRSF3, YBX1, SUPT5H, SNRNP70 |
| Signaling by FGFR | 162 | 9.66 | 22 | 0.000222 | 0.00311 | ADCY7 | MDM2, RAF1, ERBB2, EGFR, PIK3R1, CDK1, AKT1, GRB2, SRC, MAPK1, CDKN1A, CBL, SOS1, GSK3B, SHC1, UBA52, LCK, ITPR3, CREB1, PLCG1, PTPN11 |

| | | | | | | | |
|---|---|---|---|---|---|---|---|
| Interleukin-2 signaling | 42 | 2.5 | 10 | 0.000133 | 0.002 | NA | STAT5A, RAF1, PIK3R1, CDK1, GRB2, MAPK1, PTPN6, SOS1, SHC1, LCK |
| SMAD2/SMAD3:SMAD4 heterotrimer regulates transcription | 28 | 1.67 | 8 | 0.00016 | 0.00232 | RBL1, SMAD3, E2F4 | MYC, SMAD2, SMAD7, SP1, UBA52 |
| Regulation of Lipid Metabolism by Peroxisome proliferator-activated receptor alpha (PPARalpha) | 76 | 4.53 | 13 | 0.000482 | 0.00582 | RXRA, APOA5, FHL2 | CREBBP, NCOA6, MED1, PPARA, APOA1, SP1, ACOX1, NFYB, NFYC, ABCA1 |

**Supplementary Table 8.** List of enriched pathways in the network (**Figure 4A**) constructed upon the genes associated with the differentially methylated hyo- and hypermethylated promoters between the non-diseased aortic and mitral valve tissue based on the Reactome pathway database (selected results). “Total” refers to the total number of genes that belong to the particular Reactome pathway as per the chosen reference list of genes, which is hg19 in our case, “Expected” denotes the number of genes to be expected in our gene list for the particular Reactome pathway, “Hits” describes the number of genes in our list that map to the particular Reactome pathway, “P.Value” is equivalent to the enrichment p-value computed using the hypergeometric test (**see Methods**), FDR stands for false discovery rate, which is the method used to correct the corresponding p-value for multiple testing, “Seed genes” represent genes associated with significantly differentially hyper- and hypomethylated promoters and “Other genes” denote genes that are part of the minimum non-seed genes that are necessarily required to connect the seed genes to construct the network. The methylation direction of the seed genes can be found in **Supplementary Table 1**.

## 2.9 Table S9. List of enriched biological processes in the network constructed upon the genes associated with the differentially methylated hyo- and hypermethylated promoters between the non-diseased aortic and mitral valve tissue based on the Gene Ontology (GO) Biological Processes database (selected results)

| GO_BP | Total | Expected | Hits | P.Value | FDR | Seed genes | Other genes |
|---|---|---|---|---|---|---|---|
| Epidermal growth factor receptor signaling pathway | 167 | 7.56 | 38 | 6.90E-17 | 9.54E-15 | SH3KBP1, ZFYVE28, RHBDF1, ADCY7 | AP2B1, MDM2, UBB, RAF1, ERBB2, EGFR, PIK3R1, CDK1, AKT1, GRB2, SRC, MAPK1, APP, UBC, EPS15, CBL, SH3GL2, SOS1, CDC42, GSK3B, SHC1, UBA52, STAM, STAM2, ITPR3, CREB1, FASLG, PLCG1, EPS8, ARHGEF7, NCK2, PTPN11, ERBB2IP, PSEN1 |
| Positive regulation of transcription, DNA_dependent | 1260 | 57 | 123 | 6.98E-17 | 9.54E-15 | RXRA, PRPF6, RBL1, TBR1, ABLIM1, SMAD3, DYRK1B, BRCA2, ATXN7L3, MDK, LEO1, THRA, TCF21, NR2F2, FHL2, E2F4, BCL3, NCOA4, CSF3, F2R, FOXH1, PBX4, TAF8, | PAX6, KAT5, ZBTB16, TRAF6, CDK2, DDIT3, MLL, TNFRSF1A, CDH1, ATXN1, CREBBP, CHEK2, AR, RELA, NFKBIA, MYCN, UBB, EIF4A3, STAT5A, STAT3, NCOA6, TP53, SERTAD1, MYC, ILF3, SMAD2, XRCC6, ERBB2, MED1, SMAD7, SQSTM1, PIK3R1, CTNNB1, FOS, NFKB1, HIF1A, |

| | | | | | | | |
|---|---|---|---|---|---|---|---|
| | | | | | | CASK, TFEB | TCF12, PPARA, RB1, CDKN2A, AKT1, NCK1, MAPK1, SP1, NR1H4, HNF4A, HNF1A, APP, NFATC3, GATA4, USF2, EP300, UBC, IKBKB, EGR1, DLG4, COPS5, HDAC4, IGBP1, CDK5RAP3, TAF1, GSK3B, TAF7, ONECUT1, DBP, PITX3, CAND1, HDAC1, SMARCAD1, CEBPA, SIRT1, ESR1, UBA52, TRIM28, NFE2, ETS1, TCF3, TBP, JUN, NFYB, NFYC, CREB1, USF1, WDR61, PSMC5, VHL, PRKD2, RARA, NCK2, IRAK1, PHB, VDR, IRF7, DNM2, ATXN7, SUPT5H, VEGFA, SRF |

| | | | | | | | |
|---|---|---|---|---|---|---|---|
| Positive regulation of transcription from RNA polymerase II promoter | 800 | 36.2 | 91 | 1.64E-16 | 1.92E-14 | RXRA, PRPF6, RBL1, TBR1, ABLIM1, SMAD3, LEO1, THRA, TCF21, E2F4, BCL3, CSF3, FOXH1, DBP, CASK, TFEB | PAX6, KAT5, TRAF6, DDIT3, MLL, TNFRSF1A, ATXN1, AR, RELA, NFKBIA, MYCN, UBB, EIF4A3, STAT5A, STAT3, NCOA6, TP53, MYC, SMAD2, XRCC6, MED1, SMAD7, SQSTM1, PIK3R1, CTNNB1, FOS, NFKB1, HIF1A, PPARA, RB1, CDKN2A, AKT1, NCK1, SP1, NR1H4, HNF4A, HNF1A, APP, NFATC3, GATA4, USF2, EP300, UBC, EGR1, DLG4, COPS5, HDAC4, IGBP1, CDK5RAP3, TAF1, GSK3B, TAF7, ONECUT1, CAND1, HDAC1, CEBPA, SIRT1, ESR1, UBA52, ETS1, TCF3, JUN, CREB1, USF1, WDR61, PRKD2, RARA, NCK2, IRAK1, VDR, IRF7, ATXN7, SUPT5H, VEGFA, SRF |

| | | | | | | | |
|---|---|---|---|---|---|---|---|
| Transcription initiation from RNA polymerase II promoter | 219 | 9.92 | 40 | 2.96E-14 | 1.87E-12 | RXRA, RBL1, SMAD3, THRA, E2F4 | CREBBP, AR, UBB, NCOA6, MYC, SMAD2, MED1, SMAD7, PPARA, SP1, NR1H4, HNF4A, HNF1A, POLR2A, UBC, TAF10, NR3C1, TAF6, TAF1, UBE2D3, TAF7, CAND1, NEDD4L, HDAC1, ESR1, UBE2D1, UBA52, TRIM28, USP9X, MED8, TBP, RARA, VDR, ESR2, SRF |
| DNA_dependent transcription, initiation | 257 | 11.6 | 42 | 3.47E-13 | 1.67E-11 | RXRA, RBL1, POLR1C, SMAD3, THRA, E2F4 | CREBBP, AR, UBB, NCOA6, MYC, SMAD2, MED1, SMAD7, PPARA, SP1, NR1H4, HNF4A, HNF1A, BRF2, POLR2A, UBC, TAF10, NR3C1, TAF6, TAF1, UBE2D3, TAF7, CAND1, NEDD4L, HDAC1, ESR1, UBE2D1, UBA52, TRIM28, USP9X, MED8, TBP, RARA, VDR, ESR2, SRF |

| | | | | | | | |
|---|---|---|---|---|---|---|---|
| Protein modification by small protein conjugation | 713 | 32.3 | 73 | 3.76E-11 | 1.34E-09 | ERCC8, SAE1, LIMK1, LEO1, MID2, HERC6, UBOX5, DCAF4, UBE4B, DCAF8, RUSC1, G2E3 | PAX6, ZBTB16, TRAF6, UBE2I, SUMO3, UBA2, CDK2, PSMA1, MDM2, UBB, VCP, SMAD7, TOLLIP, CDK1, BARD1, NEDD8, CDKN2A, UBXN1, AKT1, UBE2H, UBE2E2, UBE2K, UBE2L3, UBE2E1, UBE2E3, UBE2B, PARK2, UBC, CUL4B, EGR1, CBL, HDAC4, CUL1, HDAC6, SUMO1, UBE2D3, UBE2D2, RCHY1, CUL3, CAND1, CUL2, LNX1, NEDD4L, UBD, SUMO2, SIRT1, UBE2C, UBA1, UBE2D1, UBA52, TRIM28, LRSAM1, MED8, TRAF2, PSMC5, VHL, PRPF19, BIRC2, CAV1, IRAK1, PSEN1 |

| | | | | | | | |
|---|---|---|---|---|---|---|---|
| Blood coagulation | 564 | 25.5 | 60 | 5.39E-10 | 1.13E-08 | F10, GNAI2, RAD51C, LCP2, TRPC3, F2R, NOS1, P2RX1, FGR, SLC7A8, PSAP, SLC16A3 | YWHAZ, CDK2, ACTB, TP53, WAS, RAF1, CRK, PIK3R1, GNA11, PLAUR, AKT1, GRB2, SRC, MAPK1, APOA1, ALB, HNF4A, APP, GATA4, PLAT, PTPN6, CBX5, GNAI1, A2M, SOS1, RAC1, CDC42, FN1, SHC1, HDAC1, LCK, NFE2, PROCR, VAV2, RAP1A, LYN, ITPR3, USF1, PLCG1, ACTG1, CAV1, PTPN11, APOE, DTNBP1, GNB1, PLA2G4A, VEGFA, SRF |
| Apoptotic process | 2130 | 96.4 | 150 | 7.53E-09 | 1.12E-07 | TNFRSF10C, ACTC1, CD44, TJP2, SMAD3, RPS3, CCAR1, BRCA2, TRIM2, THRA, SH3KBP1, TGM2, FHL2, BCL3, NGFR, F2R, PAX7, CRH, P2RX1, DNM1L, FGD1, SSTR3, UBE4B, FAIM2, AQP2, | VIM, GAPDH, ZBTB16, TLE1, GNB2L1, TSC22D1, TRAF6, YWHAZ, ARF6, DYNLL1, PDCD6IP, DDIT3, PSMA1, MLH1, MLL, TNFRSF1A, TRADD, IKBKG, CDH1, MDM2, CHEK2, AR, RELA, NFKBIA, UBB, STAT5A, TP53, MYC, SCRIB, VCP, RAF1, CTSB, EGFR, MED1, SMAD7, SQSTM1, PIK3R1, |

| | | | | | | | |
|---|---|---|---|---|---|---|---|
| | | | | | | FGD3, TMEM102, MZB1, PLEKHG5, ACSL5, CASP7, G2E3, KALRN, NQO1, PDCD6, MAEA | CTNNB1, CDK1, BARD1, NFKB1, FKBP8, HIF1A, RB1, CDKN2A, PUF60, AKT1, SRC, MAPK1, ALB, CDKN1A, APP, UBE2B, EP300, YWHAE, PTPN6, PARK2, UBC, TERF1, TJP1, NR3C1, EGR1, CBL, SOS1, IGBP1, RAC1, CDC42, KAT2A, CUL1, HDAC6, GSK3B, UBE2D3, BCL2L11, CUL3, CUL2, UBD, SNCA, HDAC1, SIRT1, ESR1, S100A9, PAK2, UBA52, LCK, HSP90AB1, SRPK2, TRAF2, SFN, VAV2, CEBPB, LYN, ETS1, JUN, FASLG, CD3E, FAS, PSMC5, KPNB1, VHL, BIRC2, CAV1, XIAP, ARHGEF7, BTK, PHB, BIRC3, PSEN1, APOE, ESR2, IRF7, DNM2, DYNLL2, GNB1, VEGFA |
| Positive regulation of I_kappaB kinase/NF_kappa B cascade | 150 | 6.79 | 23 | 2.69E-07 | 2.34E-06 | TGM2, F2R, TMEM101, PLEKHG5 | TRAF6, TNFRSF1A, TRADD, IKBKG, MAP3K3, RELA, UBB, MYD88, CTNNB1, PARK2, UBC, |

| | | | | | | | |
|---|---|---|---|---|---|---|---|
| | | | | | | | IKBKB, TERF2IP, UBD, UBA52, FASLG, BIRC2, IRAK1, BIRC3 |
| Regulation of cellular component organization | 1520 | 69 | 110 | 3.84E-07 | 3.23E-06 | MYL2, TBR1, PLK4, GFAP, PACSIN1, SMAD3, LIMK1, THRA, L3MBTL1, CDC6, SIPA1L1, SH3KBP1, CSF3, NGFR, APOA5, DNM1L, FGR, FGD1, NPPB, FGD3, PALM, CDH4, GAS2L2, DDR1, PRKCSH, RPL13A | VIM, EPB49, GNB2L1, PRMT5, ARF6, CD320, CEP76, UPF1, ATXN2, MYL12A, AR, TP53, MYC, SMAD2, VCP, RAF1, CRK, ERBB2, SMAD7, PIK3R1, CTNNB1, ETF1, RB1, CDKN2A, AKT1, GRB2, NCK1, SRC, MAPK1, APOA1, CDKN1A, HNF4A, HNF1A, UBE2E1, UBE2B, PARK2, TERF1, SPTBN1, DLG4, CBL, SH3GL2, RAC1, CDC42, POT1, HDAC6, TAF1, SUMO1, GSK3B, TAF7, FN1, WASL, XPO1, BCL2L11, TERF2IP, CAND1, TINF2, NEDD4L, UBD, SNCA, SPP1, SIRT1, PEX5, S100A9, UBE2C, UBE2D1, LRSAM1, SFN, LYN, ETS1, WDR61, FAS, CCDC85B, PRKD2, NCK2, |

| | | | | | | | |
|---|---|---|---|---|---|---|---|
| | | | | | | | PHB, PSEN1, APOE, ESR2, ANXA7, ACD, DSTN, PACSIN2, VEGFA, SRF |

**Supplementary Table 9.** List of enriched biological processes in the network (**Figure 4A**) constructed upon the genes associated with the differentially methylated hyo- and hypermethylated promoters between the non-diseased aortic and mitral valve tissue based on the Gene Ontology (GO) Biological Processes (BPs) database (selected results). “Total” refers to the total number of genes that belong to the particular GO BP as per the chosen reference list of genes, which is hg19 our case, “Expected” denotes the number of genes to be expected in our gene list for the particular GO BP, “Hits” describes the number of genes in our list that map to the particular GO BP, “ P.value” is equivalent to the enrichment p-value computed using the hypergeometric test (**see Methods**), FDR stands for false discovery rate, which is the method used to correct the corresponding p-value for multiple testing. “Seed genes” represent genes associated with significantly differentially hyper- and hypomethylated promoters and “Other genes” denote genes that are part of the minimum non-seed genes that are necessarily required to connect the seed genes to construct the network. The methylation direction of the seed genes can be found in **Supplementary Table 1**.

**2.10 Table S10. List of enriched molecular functions in the network constructed upon the genes associated with the differentially methylated hyo- and hypermethylated promoters between the non-diseased aortic and mitral valve tissue based on the Gene Ontology (GO) Molecular Functions database (selected results)**

| GO MF | Total | Expected | Hits | P.Value | FDR | Seed genes | Other genes |
|---|---|---|---|---|---|---|---|
| Transcription factor binding | 509 | 22.5 | 75 | 1.39E-20 | 2.70E-18 | RXRA, RBL1, TRIP6, SMAD3, RPS3, THRA, E2F4, BCL3, FOXH1, TCF21, FHL2 | PAX6, KAT5, TLE1, YWHAZ, UBE2I, UBA2, MDFI, DDIT3, CREBBP, AR, RELA, NFKBIA, ACTB, STAT3, NCOA6, TP53, MYC, SMAD2, MED1, CTNNB1, FOS, HIF1A, PPARA, RB1, CDKN2A, MAPK1, SP1, NR1H4, HNF4A, HNF1A, GATA4, USF2, EP300, USP7, APEX1, CBX5, EGR1, HDAC4, TAF1, SUMO1, GSK3B, TAF7, CAND1, HDAC1, CEBPA, SIRT1, ESR1, HDAC11, CSNK2B, CEBPB, ETS1, TCF3, TBP, JUN, NFYB, CREB1, USF1, PSMC5, VHL, RARA, VDR, NLK, GFI1B, SRF |

| | | | | | | | |
|---|---|---|---|---|---|---|---|
| Positive regulation of transcription, DNA_dependent | 1260 | 55.7 | 123 | 1.16E-17 | 1.50E-15 | RXRA, PRPF6, RBL1, TBR1, ABLIM1, SMAD3, DYRK1B, BRCA2, ATXN7L3, MDK, LEO1, THRA, E2F4, BCL3, NCOA4, CSF3, F2R, FOXH1, PBX4, TAF8, DBP, CASK, TFEB, TCF21, FHL2, NR2F2 | PAX6, KAT5, ZBTB16, TRAF6, CDK2, DDIT3, MLL, TNFRSF1A, CDH1, ATXN1, CREBBP, CHEK2, AR, RELA, NFKBIA, MYCN, UBB, EIF4A3, STAT5A, STAT3, NCOA6, TP53, SERTAD1, MYC, ILF3, SMAD2, XRCC6, ERBB2, MED1, SMAD7, SQSTM1, PIK3R1, CTNNB1, FOS, NFKB1, HIF1A, TCF12, PPARA, RB1, CDKN2A, AKT1, NCK1, MAPK1, SP1, NR1H4, HNF4A, HNF1A, APP, NFATC3, GATA4, USF2, EP300, UBC, IKBKB, EGR1, DLG4, COPS5, HDAC4, IGBP1, CDK5RAP3, TAF1, GSK3B, TAF7, ONECUT1, PITX3, CAND1, HDAC1, SMARCAD1, CEBPA, SIRT1, ESR1, UBA52, TRIM28, NFE2, ETS1, TCF3, TBP, JUN, NFYB, NFYC, CREB1, USF1, WDR61, PSMC5, VHL, PRKD2, RARA, NCK2, IRAK1, PHB, VDR, IRF7, DNM2, ATXN7, |

| | | | | | | | |
|---|---|---|---|---|---|---|---|
| | | | | | | | SUPT5H, VEGFA, SRF |
| Transcription from RNA polymerase II promoter | 1930 | 85.4 | 151 | 4.25E-13 | 2.75E-11 | RXRA, MAGOH, PRPF6, RBL1, TBR1, ABLIM1, NMI, SMAD3, LEO1, THRA, CDC6, WDTC1, E2F4, BCL3, CSF3, FOXH1, PBX4, DBP, SETX, HOXB3, CASK, TFEB, TCF21, FHL2, NR2F2 | PAX6, KAT5, CHD3, PRMT5, TSC22D1, TRAF6, RNPS1, SNRPB, UBE2I, SAP18, CPSF3, TRIP11, CIAO1, MDFI, DDIT3, MLL, TNFRSF1A, ATXN1, MDM2, CREBBP, AR, RELA, NFKBIA, MYCN, UBB, EIF4A3, STAT5A, STAT3, NCOA6, TP53, MYC, SMAD2, XRCC6, CRK, MED1, SMAD7, SQSTM1, PIK3R1, CTNNB1, CDK1, FOS, NFKB1, HIF1A, COPS2, TCF12, NEDD8, PPARA, |

| | | | | | | | |
|---|---|---|---|---|---|---|---|
| | | | | | | | RB1, CDKN2A, AKT1, NCK1, SP1, NR1H4, HNF4A, ZNF143, HNF1A, APP, NFATC3, GATA4, USF2, EP300, POLR2A, UBC, TAF10, NR3C1, EGR1, DLG4, TAF6, TRIM29, COPS5, CCNA1, HDAC4, IGBP1, CDK5RAP3, KAT2A, POLA1, TAF1, GSK3B, UBE2D3, UBE2D2, TAF7, ONECUT1, XPO1, CAND1, CUL2, NEDD4L, HDAC1, CEBPA, SIRT1, ESR1, UBE2D1, UBA52, TRIM28, SRSF3, USP9X, YBX1, NFE2, MED8, CEBPB, ETS1, TCF3, TBP, JUN, NFYC, CREB1, USF1, FASLG, WDR61, CEBPD, PSMC5, VHL, CAV1, PRKD2, RARA, NCK2, IRAK1, PHB, VDR, PSEN1, ESR2, IRF7, ATXN7, SUPT5H, GFI1B, VEGFA, SRF |

| | | | | | | | |
|---|---|---|---|---|---|---|---|
| Structure_specific DNA binding | 242 | 10.7 | 32 | 2.91E-08 | 7.06E-07 | RXRA, SMAD3, BRCA2, THRA, FOXH1, CTC1 | PAX6, MCM4, MCM7, MLH1, RAD51, SMAD2, XRCC6, PCBP1, EGFR, CTNNB1, FOS, NFKB1, SP1, HNF1A, USF2, EP300, TERF1, APEX1, EGR1, POT1, YBX1, RPA2, JUN, USF1, RARA, SRF |
| Transcription coactivator activity | 312 | 13.8 | 35 | 4.10E-07 | 8.84E-06 | SUPT7L, RXRA, PRPF6, DYRK1B, CCAR1, ATXN7L3, NCOA4, FHL2 | KAT5, TRIP11, RBPMS, NFKBIB, NFKB2, CREBBP, NCOA6, MED1, CTNNB1, RB1, NR1H4, UBE2L3, EP300, APEX1, TAF10, COPS5, KAT2A, TAF1, TAF7, TRIM28, NFE2, TCF3, JUN, NFYC, BIRC2, RARA, ESR2 |
| Histone deacetylase binding | 65 | 2.88 | 14 | 7.24E-07 | 1.32E-05 | WDTC1, TCF21 | TRAF6, HIF1A, SP1, YWHAE, CBX5, HDAC4, KAT2A, HDAC6, HDAC1, USF1, KPNA2, PHB |
| Chromatin binding | 338 | 15 | 32 | 4.33E-05 | 0.00041 | RXRA, SMAD3, THRA, L3MBTL1, CDC6 | TLE1, PRMT5, UPF1, CREBBP, AR, RELA, NCOA6, TP53, SMAD2, DHX30, MED1, CTNNB1, NFATC3, GATA4, EP300, TERF1, APEX1, CBX5, KAT2A, POLA1, ESR1, TCF3, RARA, ATXN7, DDX1, CDC5L, SRF |

| | | | | | | | |
|---|---|---|---|---|---|---|---|
| Histone acetyltransferase activity | 52 | 2.3 | 9 | 0.000411 | 0.00319 | SUPT7L, BRCA2 | KAT5, KAT7, CREBBP, EP300, TAF10, KAT2A, TAF1 |
| RNA binding | 976 | 43.2 | 65 | 0.00059 | 0.00432 | CARHSP1, LSM2, GSPT1, MAGOH, PRPF6, TRA2A, RPS3, RPS7, THRA, STAU1, ISG20, DDX19B, PPIE, RPL11, CELF3, MRPS17, PRKCSH, NUDT7 | RPL18A, RNPS1, SNRPB, PINX1, SNRPA1, CPSF3, HNRNPM, RBPMS, PSMA1, EWSR1, NAA38, UPF1, LSM4, ATXN1, ATXN2, EIF4A3, PA2G4, ILF3, PCBP1, SF3A1, DHX30, BARD1, ETF1, PUF60, PABPC1, SRRM2, RPL5, CARS, APEX1, COPS5, XPO1, HNRNPF, ELAVL1, THOC5, NCL, HNRNPU, PTBP1, SRSF3, EEF1A1, YBX1, QKI, RPL3, PSPC1, DDX1, CDC5L, EIF6, SNRNP70 |
| NF_kappaB binding | 25 | 1.11 | 6 | 0.000632 | 0.00446 | RPS3 | RELA, NFKBIA, CDKN2A, APEX1, GSK3B |
| SMAD binding | 68 | 3.01 | 10 | 0.000768 | 0.00523 | SMAD3, FOXH1 | PAX6, SMAD2, SMAD7, CTNNB1, FOS, GATA4, USP9X, JUN |

**Supplementary Table 10.** List of enriched molecular functions in the network (**Figure 4A**) constructed upon the genes associated with the differentially methylated hyo- and hypermethylated promoters between the non-diseased aortic and mitral valve tissue based on the Gene Ontology (GO) Molecular Functions (MFs) database (selected results). “Total” refers to the total number of genes that belong to the particular GO MF as per the chosen reference list of genes, which is hg19 in our case, “Expected” denotes the number of genes to be expected in our gene list for the particular GO MF, “Hits” describes the number of genes in our list that map to the particular GO MF, “P.value” is equivalent to the enrichment p-value computed using the hypergeometric test (**see Methods**), FDR stands for false discovery rate, which is the method used to correct the corresponding p-value for multiple testing, “Seed genes” represent genes associated with significantly differentially hyper- and

hypomethylated promoters and “Other genes” denote genes that are part of the minimum non-seed genes that are necessarily required to connect the seed genes to construct the network. The methylation direction of the seed genes can be found in **Supplementary Table 1**.

## 2.11 Table S11. List of enriched cellular components in the network constructed upon the genes associated with the differentially methylated hyo- and hypermethylated promoters between the non-diseased aortic and mitral valve tissue based on the Gene Ontology (GO) Cellular Components database (selected results)

| GO CC | Total | Expected | Hits | P.Value | FDR | Seed genes | Other genes |
|---|---|---|---|---|---|---|---|
| Transcription factor complex | 303 | 12.2 | 40 | 3.36E-11 | 7.57E-10 | RBL1, SMAD3, E2F4, FOXH1, TAF8, TFEB | KAT5, TLE1, CDK2, HOXA9, CREBBP, RELA, NCOA6, TP53, SMAD2, XRCC6, SMAD7, CTNNB1, FOS, HIF1A, TCF12, RB1, HNF1A, EP300, APEX1, TAF10, TAF6, KAT2A, TAF1, TAF7, CEBPA, ETS1, TCF3, TBP, JUN, NFYB, NFYC, CREB1, USF1, GFI1B |
| Spliceosomal complex | 161 | 6.47 | 21 | 1.99E-06 | 1.82E-05 | LSM2, MAGOH, PRPF6, PPIE, XAB2 | SNRPB, SNRPA1, HNRNPM, UPF1, SNRPD2, EIF4A3, SF3A1, PABPC1, SRRM2, HSPA8, HNRNPF, HNRNPU, YBX1, PRPF19, CDC5L, SNRNP70 |
| Cytoskeleton | 2200 | 88.3 | 128 | 6.94E-06 | 5.38E-05 | MYO15A, ACTC1, MYL2, GNAI2, TRIP6, KRT8, PLK4, HOMER3, ABLIM1, GFAP, RGS14, CORO1B, TUBA8, NDE1, BRCA2, USH1C, RPS7, CDC6, SIPA1L1, STAU1, SH3KBP1, MID2, FBF1, CCP110, NOS1, DNM1L, FGR, | VIM, CRMP1, GAPDH, CHD3, EPB49, NDRG1, GNB2L1, YWHAZ, PINX1, DYNLL1, PDCD6IP, TRIP11, CIAO1, CDK2, MTUS2, PSMA1, TUBGCP4, CEP76, TRADD, CDH1, MYL12A, ACTB, MYC, WAS, CTNNB1, CDK1, TRIM55, RB1, AKT1, SRC, MAPK1, APP, TUBB, IQGAP1, YWHAE, TERF1, APEX1, TPM4, SPTBN1, GNAI1, ABI1, DLG4, TRPC4, CCNA1, HDAC4, CDC42, KAT2A, HDAC6, TUBA1A, GSK3B, WASL, BCL2L11, CUL3, SNCA, THOC5, S100A9, IQCB1, LCK, 11-Sep, NFE2, PROCR, TUBB2B, MPDZ, LYN, |

| | | | | | | | |
|---|---|---|---|---|---|---|---|
| | | | | | | FGD1, DCTN4, KRT73, FGD3, RUSC1, KRT38, HSD17B14, SLC16A3, GAS2L2, KRT83, CLEC11A, MYO7B, TCTN2, EXOC7, CASK, KALRN, ZNF44, SHANK2, PPP4R2, MAEA, FHL2 | TRIM63, ACTG1, TNNT1, CKAP4, EPS8, CCDC85B, PRPF19, PSEN1, DNM2, CTTN, ATXN7, DYNLL2, DTNBP1, ACTA2, DSTN, FLOT1, EIF6 |
| Transcription factor TFIID complex | 21 | 0.844 | 7 | 1.17E-05 | 8.74E-05 | TAF8 | TP53, TAF10, TAF6, TAF1, TAF7, TBP |
| Nuclear chromatin | 159 | 6.39 | 19 | 2.16E-05 | 0.000155 | RXRA, SIRT6 | PAX6, CREBBP, AR, TP53, NR1H4, UBE2B, CBX5, HDAC1, SIRT1, TRIM28, CEBPB, TCF3, TBP, JUN, CREB1, RARA, SRF |
| Chromatin | 326 | 13.1 | 30 | 2.21E-05 | 0.000155 | RXRA, L3MBTL1, SIRT6 | PAX6, KAT5, MCM7, UPF1, CREBBP, AR, MYCN, TP53, RB1, CDKN2A, NR1H4, UBE2B, CBX5, POLA1, HDAC1, SMARCAD1, SIRT1, HIST3H3, TRIM28, CEBPB, TCF3, TBP, JUN, CREB1, WDR61, RARA, SRF |
| Actin cytoskeleton | 430 | 17.3 | 30 | 0.00247 | 0.0096 | MYO15A, ACTC1, MYL2, ABLIM1, CORO1B, FGR, | EPB49, CDH1, MYL12A, WAS, IQGAP1, TPM4, SPTBN1, HDAC4, WASL, SNCA, 11-Sep, NFE2, ACTG1, TNNT1, DYNLL2, ACTA2 |

| | | | | | | | |
|---|---|---|---|---|---|---|---|
| | | | | | | SLC16A3, GAS2L2, MYO7B, CASK, KALRN, MAEA, FHL2 | |
| Histone deacetylase complex | 53 | 2.13 | 7 | 0.00507 | 0.0175 | NA | CHD3, SAP18, CBX5, HDAC4, HDAC6, HDAC1, HDAC11 |

**Supplementary Table 11.** List of enriched cellular components in the network (**Figure 4A**) constructed upon the genes associated with the differentially methylated hyo- and hypermethylated promoters between the non-diseased aortic and mitral valve tissue based on the Gene Ontology (GO) Cellular Components (CCs) database (selected results). "Total" refers to the total number of genes that belong to the particular GO CC as per the chosen reference list of genes, which is hg19 in our case, "Expected" denotes the number of genes to be expected in our gene list for the particular GO CC, "Hits" describes the number of genes in our list that map to the particular GO CC, "P.value" is equivalent to the enrichment p-value computed using the hypergeometric test (**see Methods**), FDR stands for false discovery rate, which is the method used to correct the corresponding p-value for multiple testing, "Seed genes" represent genes associated with significantly differentially hyper- and hypomethylated promoters and "Other genes" denote genes that are part of the minimum non-seed genes that are necessarily required to connect the seed genes to construct the network. The methylation direction of the seed genes can be found in **Supplementary Table 1**.

s

## 2.12 Table S12. Node characteristics of subnetwork 1/module 1 of the NW (pvalue 3.04 e-13)

| Node No. | Gene name | Degree | Betweenness Centrality | Meth.diff |
|---|---|---|---|---|
| 1 | UBC | 43 | 939 | 0 |
| 2 | UBQLN4 | 4 | 3 | 0 |
| 3 | PMEPA1 | 2 | 10 | -31.0166 |
| 4 | ITPRIPL1 | 2 | 10 | -12.7256 |
| 5 | UBR7 | 2 | 10 | -11.1156 |
| 6 | CYB5R1 | 2 | 10 | -10.7196 |
| 7 | WNT5B | 1 | 0 | -14.1133 |
| 8 | SEMA4B | 1 | 0 | 14.38911 |
| 9 | UCKL1 | 1 | 0 | -11.8067 |
| 10 | SLC7A8 | 1 | 0 | -40.7436 |
| 11 | AQP2 | 1 | 0 | -21.134 |
| 12 | RCCD1 | 1 | 0 | -19.0998 |
| 13 | TOP1MT | 1 | 0 | 15.78471 |
| 14 | IBA57 | 1 | 0 | 11.57786 |
| 15 | OSBPL1A | 1 | 0 | 10.37672 |
| 16 | PLIN4 | 1 | 0 | -10.4404 |
| 17 | PLBD2 | 1 | 0 | -17.4555 |
| 18 | SPA17 | 1 | 0 | 14.31302 |
| 19 | C6orf170 | 1 | 0 | -41.2808 |
| 20 | ALS2CL | 1 | 0 | 10.70251 |
| 21 | SLC16A3 | 1 | 0 | 13.40508 |
| 22 | TOR4A | 1 | 0 | -10.1032 |
| 23 | SLC6A8 | 1 | 0 | 11.05677 |
| 24 | SYCP1 | 1 | 0 | 14.5074 |
| 25 | AP1S3 | 1 | 0 | -10.957 |
| 26 | FUCA2 | 1 | 0 | 11.09653 |
| 27 | ACSL5 | 1 | 0 | -16.8043 |
| 28 | PXMP4 | 1 | 0 | -10.516 |
| 29 | ZNF551 | 1 | 0 | 17.70154 |

| | | | | |
|---|---|---|---|---|
| 30 | NUDT22 | 1 | 0 | -11.9831 |
| 31 | CLEC11A | 1 | 0 | -24.7239 |
| 32 | GRTP1 | 1 | 0 | -11.8419 |
| 33 | SLC2A10 | 1 | 0 | -17.9246 |
| 34 | ZNF597 | 1 | 0 | 10.88286 |
| 35 | NUP210L | 1 | 0 | -14.658 |
| 36 | ZNF354C | 1 | 0 | -17.1657 |
| 37 | MYO7B | 1 | 0 | -12.8972 |
| 38 | LDLRAD4 | 1 | 0 | -10.4618 |
| 39 | GSTO2 | 1 | 0 | 10.83864 |
| 40 | ASAH2B | 1 | 0 | 15.99461 |
| 41 | CTC1 | 1 | 0 | -17.328 |
| 42 | SERHL2 | 1 | 0 | -11.0445 |
| 43 | IQCA1 | 1 | 0 | -15.9873 |
| 44 | DNAAF3 | 1 | 0 | -14.7451 |
| 45 | RHBDF1 | 1 | 0 | 10.29471 |

**Supplementary Table 12.** Node characteristics of subnetwork 1/module 1 of the network in **Figure 4B** (pvalue 3.04 e-13). Each node represents a gene. Degree refers to the number of connections/edges/PPI-interactions that a node has to other nodes and betweenness centrality reflects the number of shortest paths passing through a node (2).

**2.13 Table S13. List of enriched pathways, biological processes, molecular functions and cellular components in subnetwork 1/module 1 (pvalue 3.04 e-13) of the network constructed upon the genes associated with the differentially methylated hyo- and hypermethylated promoters between the non-diseased aortic and mitral valve tissue based on KEGG/Reactome, Gene Ontology (GO) Biological Processes database, Gene Ontology (GO) Molecular Functions database and Gene Ontology (GO) Cellular Components database (full list)**

| **KEGG** | | | | | |
|---|---|---|---|---|---|
| **Pathway** | **Total** | **Expected** | **Hits** | **P.Value** | **FDR** |
| PPAR signaling pathway | 74 | 0.172 | 2 | 0.0125 | 1 |
| Drug metabolism - other enzymes | 79 | 0.184 | 2 | 0.0142 | 1 |
| Peroxisome | 83 | 0.193 | 2 | 0.0156 | 1 |
| Fatty acid biosynthesis | 18 | 0.0419 | 1 | 0.0411 | 1 |
| Other glycan degradation | 18 | 0.0419 | 1 | 0.0411 | 1 |
| Axon guidance | 181 | 0.421 | 2 | 0.0652 | 1 |
| Ferroptosis | 40 | 0.0931 | 1 | 0.0892 | 1 |
| Fatty acid degradation | 44 | 0.102 | 1 | 0.0977 | 1 |
| Vasopressin-regulated water reabsorption | 44 | 0.102 | 1 | 0.0977 | 1 |
| Herpes simplex infection | 492 | 1.14 | 3 | 0.102 | 1 |
| **Reactome** | | | | | |
| **Pathway** | **Total** | **Expected** | **Hits** | **P.Value** | **FDR** |
| Basigin interactions | 32 | 0.0671 | 2 | 0.00195 | 1 |
| Proton-coupled monocarboxylate transport | 4 | 0.00839 | 1 | 0.00836 | 1 |
| SLC-mediated transmembrane transport | 251 | 0.526 | 3 | 0.014 | 1 |
| Transport of glucose and other sugars, bile salts and organic acids, metal ions and amine compounds | 95 | 0.199 | 2 | 0.0163 | 1 |
| Vitamin C (ascorbate) metabolism | 8 | 0.0168 | 1 | 0.0167 | 1 |
| Transmembrane transport of small molecules | 504 | 1.06 | 4 | 0.0175 | 1 |
| Cell surface interactions at the vascular wall | 99 | 0.208 | 2 | 0.0176 | 1 |
| Creatine metabolism | 9 | 0.0189 | 1 | 0.0187 | 1 |

| | | | | | |
|---|---|---|---|---|---|
| MHC class II antigen presentation | 118 | 0.247 | 2 | 0.0245 | 1 |
| Bile salt and organic anion SLC transporters | 12 | 0.0252 | 1 | 0.0249 | 1 |
| Facilitative Na+-independent glucose transporters | 12 | 0.0252 | 1 | 0.0249 | 1 |
| Passive Transport by Aquaporins | 12 | 0.0252 | 1 | 0.0249 | 1 |
| Nef mediated downregulation of MHC class I complex cell surface expression | 13 | 0.0273 | 1 | 0.0269 | 1 |
| Synthesis of very long-chain fatty acyl-CoAs | 14 | 0.0294 | 1 | 0.029 | 1 |
| Fatty Acyl-CoA Biosynthesis | 18 | 0.0377 | 1 | 0.0371 | 1 |
| Pyruvate metabolism | 21 | 0.044 | 1 | 0.0432 | 1 |
| Nef-mediates down modulation of cell surface receptors by recruiting them to clathrin adapters | 24 | 0.0503 | 1 | 0.0492 | 1 |
| **GO BP** | | | | | |
| **Biological Process** | **Total** | **Expected** | **Hits** | **P.Value** | **FDR** |
| Organic acid transport | 246 | 0.551 | 3 | 0.0173 | 1 |
| Carboxylic acid transport | 246 | 0.551 | 3 | 0.0173 | 1 |
| Meiosis I | 91 | 0.204 | 2 | 0.0176 | 1 |
| Regulation of transforming growth factor beta receptor signaling pathway | 107 | 0.24 | 2 | 0.0238 | 1 |
| Amino acid transport | 143 | 0.32 | 2 | 0.0406 | 1 |
| Carbohydrate transport | 147 | 0.329 | 2 | 0.0426 | 1 |
| Neutral amino acid transport | 23 | 0.0515 | 1 | 0.0503 | 1 |
| Anion transport | 374 | 0.838 | 3 | 0.0504 | 1 |
| Epidermal growth factor receptor signaling pathway | 167 | 0.374 | 2 | 0.0536 | 1 |
| Carboxylic acid metabolic process | 1270 | 2.85 | 6 | 0.0605 | 1 |
| **GO MF** | | | | | |
| **Molecular Function** | **Total** | **Expected** | **Hits** | **P.Value** | **FDR** |
| Carbohydrate transmembrane transporter activity | 33 | 0.0775 | 2 | 0.0027 | 0.365 |
| Carboxylic acid transmembrane transporter activity | 127 | 0.298 | 3 | 0.00323 | 0.365 |

| | | | | | |
|---|---|---|---|---|---|
| Organic acid transmembrane transporter activity | 130 | 0.305 | 3 | 0.00345 | 0.365 |
| Symporter activity | 134 | 0.315 | 3 | 0.00376 | 0.365 |
| Organic anion transmembrane transporter activity | 147 | 0.345 | 3 | 0.00487 | 0.378 |
| Secondary active transmembrane transporter activity | 212 | 0.498 | 3 | 0.0133 | 0.857 |
| Amino acid transmembrane transporter activity | 84 | 0.197 | 2 | 0.0165 | 0.917 |
| GTPase activator activity | 267 | 0.627 | 3 | 0.0243 | 1 |
| Ras GTPase activator activity | 117 | 0.275 | 2 | 0.0307 | 1 |
| Anion transmembrane transporter activity | 304 | 0.714 | 3 | 0.0339 | 1 |
| Small GTPase regulator activity | 319 | 0.749 | 3 | 0.0383 | 1 |
| Cofactor transporter activity | 20 | 0.047 | 1 | 0.046 | 1 |
| Active transmembrane transporter activity | 347 | 0.815 | 3 | 0.0472 | 1 |
| **GO CC** | | | | | |
| **Cellular Component** | **Total** | **Expected** | **Hits** | **P.Value** | **FDR** |
| Lytic vacuole | 401 | 0.938 | 4 | 0.014 | 1 |
| Lysosome | 401 | 0.938 | 4 | 0.014 | 1 |
| Vacuole | 486 | 1.14 | 4 | 0.0262 | 1 |
| Vacuolar part | 279 | 0.653 | 3 | 0.0272 | 1 |
| Trans_Golgi network | 120 | 0.281 | 2 | 0.032 | 1 |
| Lysosomal membrane | 153 | 0.358 | 2 | 0.0497 | 1 |
| Basolateral plasma membrane | 162 | 0.379 | 2 | 0.055 | 1 |
| Peroxisome | 181 | 0.423 | 2 | 0.0669 | 1 |
| Microbody | 181 | 0.423 | 2 | 0.0669 | 1 |
| Cytoplasmic vesicle membrane | 403 | 0.943 | 3 | 0.0676 | 1 |

**Supplementary Table 13.** List of enriched pathways, biological processes, molecular functions and cellular components in subnetwork 1/module 1 (pvalue 3.04 e-13, **Figure 4B**) of the network constructed upon the genes associated with the differentially methylated hyo- and hypermethylated promoters between the non-diseased aortic and mitral valve tissue based on KEGG/Reactome, Gene Ontology (GO) Biological Processes database, Gene Ontology (GO) Molecular Functions database and Gene Ontology (GO) Cellular Components database (full list). “Total” refers to the total number of genes that belong to a particular category as per the chosen reference list of genes, which is hg19 in

our case, "Expected" denotes the number of genes to be expected in our gene list for a particular category, "Hits" describes the number of genes in our list that map to a particular category, "P.value" is equivalent to the enrichment p-value computed using the hypergeometric test (**see Methods**) and FDR stands for false discovery rate, which is the method used to correct the corresponding p-value for multiple testing.

## 2.14 Table S14. Node characteristics of subnetwork 2/module 2 of the NW (pvalue 0.047)

| Node No. | Gene name | Degree | Betweenness Centrality | Meth.diff |
|---|---|---|---|---|
| 1 | BRCA2 | 5 | 33.5 | 16.35677 |
| 2 | APOA5 | 4 | 32.5 | 15.30117 |
| 3 | USF2 | 2 | 12.5 | 0 |
| 4 | USF1 | 2 | 12.5 | 0 |
| 5 | PPARA | 2 | 10 | 0 |
| 6 | NR1H4 | 2 | 10 | 0 |
| 7 | RPA2 | 2 | 10 | 0 |
| 8 | FANCD2 | 1 | 0 | 0 |
| 9 | BARD1 | 1 | 0 | 0 |
| 10 | FABP6 | 1 | 0 | 14.57521 |
| 11 | SLC10A2 | 1 | 0 | 19.11392 |
| 12 | SLC17A9 | 1 | 0 | 18.99397 |

**Supplementary Table 14.** Node characteristics of subnetwork 2/module 2 of the network in **Figure 4C** (pvalue 0.047). Each node represents a gene. Degree refers to the number of connections/edges/PPI-interactions that a node has to other nodes and betweenness centrality reflects the number of shortest paths passing through a node (2).

**2.15 Table S15. List of enriched pathways, biological processes, molecular functions and cellular components in subnetwork 2/module 2 (pvalue 0.047) of the network constructed upon the genes associated with the differentially methylated hyo- and hypermethylated promoters between the non-diseased aortic and mitral valve tissue based on KEGG/Reactome, Gene Ontology (GO) Biological Processes database, Gene Ontology (GO) Molecular Functions database and Gene Ontology (GO) Cellular Components database (full list)**

| **KEGG** | | | | | |
|---|---|---|---|---|---|
| **Pathway** | **Total** | **Expected** | **Hits** | **P.Value** | **FDR** |
| **Homologous recombination** | 41 | 0.0477 | 3 | 1.14E-05 | 0.00361 |
| Fanconi anemia pathway | 54 | 0.0628 | 3 | 2.62E-05 | 0.00417 |
| **PPAR signaling pathway** | 74 | 0.0861 | 3 | 6.77E-05 | 0.00718 |
| Bile secretion | 72 | 0.0838 | 2 | 0.00295 | 0.234 |
| Mismatch repair | 23 | 0.0268 | 1 | 0.0265 | 1 |
| DNA replication | 36 | 0.0419 | 1 | 0.0411 | 1 |
| Nucleotide excision repair | 47 | 0.0547 | 1 | 0.0534 | 1 |
| Adipocytokine signaling pathway | 69 | 0.0803 | 1 | 0.0775 | 1 |
| Pancreatic cancer | 75 | 0.0872 | 1 | 0.084 | 1 |
| Glucagon signaling pathway | 103 | 0.12 | 1 | 0.114 | 1 |
| **Reactome** | | | | | |
| **Pathway** | **Total** | **Expected** | **Hits** | **P.Value** | **FDR** |
| **Homologous DNA pairing and strand exchange** | 6 | 0.00719 | 2 | 1.88E-05 | 0.0132 |
| Presynaptic phase of homologous DNA pairing and strand exchange | 6 | 0.00719 | 2 | 1.88E-05 | 0.0132 |
| Recycling of bile acids and salts | 11 | 0.0132 | 2 | 6.87E-05 | 0.0321 |
| **Homologous recombination repair of replication-independent double-strand breaks** | 16 | 0.0192 | 2 | 0.000149 | 0.0419 |
| **Homologous Recombination Repair** | 16 | 0.0192 | 2 | 0.000149 | 0.0419 |
| DNA Repair | 117 | 0.14 | 3 | 0.000275 | 0.0644 |
| Double-Strand Break Repair | 24 | 0.0288 | 2 | 0.000342 | 0.0685 |
| Bile acid and bile salt metabolism | 28 | 0.0335 | 2 | 0.000468 | 0.0728 |
| Fanconi Anemia pathway | 28 | 0.0335 | 2 | 0.000468 | 0.0728 |
| Nuclear Receptor transcription pathway | 53 | 0.0635 | 2 | 0.00168 | 0.229 |

| | | | | | |
|---|---|---|---|---|---|
| Metabolism of lipids and lipoproteins | 507 | 0.607 | 4 | 0.0018 | 0.229 |
| PPARA Activates Gene Expression | 59 | 0.0707 | 2 | 0.00208 | 0.243 |
| Regulation of Lipid Metabolism by Peroxisome proliferator-activated receptor alpha (PPARalpha) | 76 | 0.0911 | 2 | 0.00342 | 0.36 |
| Processing of DNA double-strand break ends | 3 | 0.00359 | 1 | 0.00359 | 0.36 |
| Meiotic Recombination | 84 | 0.101 | 2 | 0.00417 | 0.39 |
| Assembly of the RAD51-ssDNA nucleoprotein complex | 5 | 0.00599 | 1 | 0.00598 | 0.524 |
| Meiosis | 117 | 0.14 | 2 | 0.00796 | 0.656 |
| Fatty acid, triacylglycerol, and ketone body metabolism | 139 | 0.167 | 2 | 0.0111 | 0.864 |
| Removal of the Flap Intermediate from the C-strand | 10 | 0.012 | 1 | 0.0119 | 0.88 |
| Processive synthesis on the C-strand of the telomere | 11 | 0.0132 | 1 | 0.0131 | 0.919 |
| Regulation of the Fanconi anemia pathway | 13 | 0.0156 | 1 | 0.0155 | 0.952 |
| Removal of the Flap Intermediate | 14 | 0.0168 | 1 | 0.0167 | 0.952 |
| BMAL1:CLOCK/NPAS2 Activates Circadian Expression | 14 | 0.0168 | 1 | 0.0167 | 0.952 |
| Processive synthesis on the lagging strand | 15 | 0.018 | 1 | 0.0178 | 0.952 |
| Repair synthesis of patch ~27-30 bases long by DNA polymerase | 15 | 0.018 | 1 | 0.0178 | 0.952 |
| Repair synthesis for gap-filling by DNA polymerase in TC-NER | 15 | 0.018 | 1 | 0.0178 | 0.952 |
| Gap-filling DNA repair synthesis and ligation in GG-NER | 16 | 0.0192 | 1 | 0.019 | 0.952 |
| Gap-filling DNA repair synthesis and ligation in TC-NER | 16 | 0.0192 | 1 | 0.019 | 0.952 |
| Lagging Strand Synthesis | 20 | 0.024 | 1 | 0.0237 | 1 |
| Telomere C-strand (Lagging Strand) Synthesis | 22 | 0.0264 | 1 | 0.0261 | 1 |
| Dual incision reaction in GG-NER | 23 | 0.0276 | 1 | 0.0272 | 1 |

| | | | | | |
|---|---|---|---|---|---|
| Formation of incision complex in GG-NER | 23 | 0.0276 | 1 | 0.0272 | 1 |
| Extension of Telomeres | 24 | 0.0288 | 1 | 0.0284 | 1 |
| DNA strand elongation | 31 | 0.0371 | 1 | 0.0366 | 1 |
| Activation of the pre-replicative complex | 32 | 0.0383 | 1 | 0.0377 | 1 |
| Circadian Clock | 35 | 0.0419 | 1 | 0.0412 | 1 |
| Global Genomic NER (GG-NER) | 36 | 0.0431 | 1 | 0.0423 | 1 |
| Activation of ATR in response to replication stress | 41 | 0.0491 | 1 | 0.0481 | 1 |
| **GO BP** | | | | | |
| **Biological Process** | **Total** | **Expected** | **Hits** | **P.Value** | **FDR** |
| Bile acid metabolic process | 59 | 0.0496 | 3 | 1.43E-05 | 0.0118 |
| Monocarboxylic acid metabolic process | 567 | 0.476 | 5 | 6.07E-05 | 0.0127 |
| Monocarboxylic acid transport | 103 | 0.0865 | 3 | 7.63E-05 | 0.0127 |
| **Lipid homeostasis** | 103 | 0.0865 | 3 | 7.63E-05 | 0.0127 |
| **Response to nutrient levels** | 295 | 0.248 | 4 | 7.73E-05 | 0.0127 |
| **Response to extracellular stimulus** | 320 | 0.269 | 4 | 0.000106 | 0.0145 |
| **Cellular response to nutrient levels** | 126 | 0.106 | 3 | 0.000139 | 0.0163 |
| **Response to nutrient** | 147 | 0.123 | 3 | 0.000219 | 0.0208 |
| **Cellular response to extracellular stimulus** | 149 | 0.125 | 3 | 0.000228 | 0.0208 |
| DNA repair | 538 | 0.452 | 4 | 0.000773 | 0.0624 |
| Organic acid transport | 246 | 0.207 | 3 | 0.000989 | 0.0624 |
| Regulation of lipid metabolic process | 246 | 0.207 | 3 | 0.000989 | 0.0624 |
| Carboxylic acid transport | 246 | 0.207 | 3 | 0.000989 | 0.0624 |
| Positive regulation of transcription, DNA_dependent | 1260 | 1.06 | 5 | 0.00245 | 0.115 |
| Positive regulation of transcription, DNA_dependent | 1260 | 1.06 | 5 | 0.00245 | 0.115 |
| Meiosis I | 91 | 0.0764 | 2 | 0.00254 | 0.115 |
| Carboxylic acid metabolic process | 1270 | 1.07 | 5 | 0.0026 | 0.115 |
| Response to radiation | 345 | 0.29 | 3 | 0.00261 | 0.115 |

| | | | | | |
|---|---|---|---|---|---|
| Positive regulation of metabolic process | 2690 | 2.26 | 7 | 0.00272 | 0.115 |
| Steroid metabolic process | 361 | 0.303 | 3 | 0.00297 | 0.115 |
| Positive regulation of RNA metabolic process | 1330 | 1.12 | 5 | 0.00317 | 0.115 |
| Anion transport | 374 | 0.314 | 3 | 0.00328 | 0.115 |
| Nucleotide_excision repair | 104 | 0.0873 | 2 | 0.0033 | 0.115 |
| Positive regulation of transcription from RNA polymerase II promoter | 800 | 0.672 | 4 | 0.00336 | 0.115 |
| Response to UV | 112 | 0.0941 | 2 | 0.00382 | 0.12 |
| Response to ionizing radiation | 112 | 0.0941 | 2 | 0.00382 | 0.12 |
| Organic acid metabolic process | 1430 | 1.2 | 5 | 0.0043 | 0.124 |
| Response to DNA damage stimulus | 862 | 0.724 | 4 | 0.00441 | 0.124 |
| DNA_dependent DNA replication | 121 | 0.102 | 2 | 0.00444 | 0.124 |
| Lipoprotein metabolic process | 124 | 0.104 | 2 | 0.00466 | 0.124 |
| Response to abiotic stimulus | 876 | 0.736 | 4 | 0.00467 | 0.124 |
| Positive regulation of nucleobase_containing compound metabolic process | 1490 | 1.25 | 5 | 0.00518 | 0.133 |
| Homeostatic process | 1510 | 1.26 | 5 | 0.00542 | 0.135 |
| Double_strand break repair | 139 | 0.117 | 2 | 0.00582 | 0.14 |
| S phase of mitotic cell cycle | 144 | 0.121 | 2 | 0.00623 | 0.146 |
| S phase | 153 | 0.128 | 2 | 0.00701 | 0.16 |
| Lipid metabolic process | 1730 | 1.45 | 5 | 0.00981 | 0.214 |
| Meiotic cell cycle | 183 | 0.154 | 2 | 0.0099 | 0.214 |
| Cellular lipid catabolic process | 188 | 0.158 | 2 | 0.0104 | 0.218 |
| Positive regulation of cellular metabolic process | 2530 | 2.12 | 6 | 0.0106 | 0.218 |
| Negative regulation of nucleobase_containing compound metabolic process | 1130 | 0.95 | 4 | 0.0115 | 0.231 |
| Regulation of catabolic process | 595 | 0.5 | 3 | 0.0119 | 0.233 |
| Transcription initiation from RNA polymerase II promoter | 219 | 0.184 | 2 | 0.014 | 0.266 |
| Response to light stimulus | 229 | 0.192 | 2 | 0.0152 | 0.283 |

| | | | | | |
|---|---|---|---|---|---|
| Regulation of DNA metabolic process | 235 | 0.197 | 2 | 0.0159 | 0.286 |
| DNA metabolic process | 1240 | 1.05 | 4 | 0.0161 | 0.286 |
| Viral infectious cycle | 241 | 0.202 | 2 | 0.0167 | 0.292 |
| Response to hypoxia | 245 | 0.206 | 2 | 0.0173 | 0.293 |
| DNA recombination | 247 | 0.207 | 2 | 0.0175 | 0.293 |
| Wound healing | 700 | 0.588 | 3 | 0.0185 | 0.303 |
| DNA_dependent transcription, initiation | 257 | 0.216 | 2 | 0.0189 | 0.304 |
| Lipid transport | 268 | 0.225 | 2 | 0.0204 | 0.321 |
| Intracellular receptor mediated signaling pathway | 270 | 0.227 | 2 | 0.0207 | 0.321 |
| Response to endogenous stimulus | 1360 | 1.15 | 4 | 0.0219 | 0.332 |
| Response to hormone stimulus | 751 | 0.631 | 3 | 0.0223 | 0.332 |
| Glucose metabolic process | 290 | 0.244 | 2 | 0.0237 | 0.341 |
| Developmental growth | 290 | 0.244 | 2 | 0.0237 | 0.341 |
| Cell cycle process | 1420 | 1.19 | 4 | 0.0248 | 0.35 |
| Gland development | 303 | 0.254 | 2 | 0.0257 | 0.356 |
| Lipid catabolic process | 305 | 0.256 | 2 | 0.026 | 0.356 |
| Negative regulation of DNA binding | 37 | 0.0311 | 1 | 0.0306 | 0.406 |
| Response to external stimulus | 1510 | 1.27 | 4 | 0.0307 | 0.406 |
| Response to stress | 4150 | 3.49 | 7 | 0.0325 | 0.421 |
| DNA replication | 346 | 0.291 | 2 | 0.0329 | 0.421 |
| Chromosome organization | 878 | 0.737 | 3 | 0.0335 | 0.423 |
| Regulation of cell cycle | 886 | 0.744 | 3 | 0.0343 | 0.425 |
| Negative regulation of DNA replication | 42 | 0.0353 | 1 | 0.0347 | 0.425 |
| Regulation of transcription from RNA polymerase II promoter | 1610 | 1.35 | 4 | 0.0377 | 0.455 |
| Centrosome cycle | 47 | 0.0395 | 1 | 0.0388 | 0.457 |
| Cellular response to stress | 1620 | 1.36 | 4 | 0.039 | 0.457 |
| Protein export from nucleus | 48 | 0.0403 | 1 | 0.0396 | 0.457 |
| Intrinsic apoptotic signaling pathway in response to DNA damage | 51 | 0.0428 | 1 | 0.042 | 0.469 |
| Negative regulation of cellular metabolic process | 1660 | 1.4 | 4 | 0.0421 | 0.469 |

| | | | | | |
|---|---|---|---|---|---|
| Fatty acid metabolic process | 397 | 0.333 | 2 | 0.0423 | 0.469 |
| Regulation of biological quality | 3400 | 2.85 | 6 | 0.0435 | 0.476 |
| Cell cycle arrest | 428 | 0.359 | 2 | 0.0484 | 0.512 |
| Reproductive process | 1740 | 1.46 | 4 | 0.0489 | 0.512 |
| Negative regulation of RNA metabolic process | 1020 | 0.86 | 3 | 0.0496 | 0.512 |
| Interphase of mitotic cell cycle | 435 | 0.365 | 2 | 0.0499 | 0.512 |
| **GO MF** | | | | | |
| **Molecular Function** | **Total** | **Expected** | **Hits** | **P.Value** | **FDR** |
| **Structure_specific DNA binding** | 242 | 0.184 | 4 | 2.29E-05 | 0.00888 |
| **RNA polymerase II distal enhancer sequence_specific DNA binding transcription factor activity** | 110 | 0.0836 | 3 | 6.73E-05 | 0.0131 |
| **Enzyme binding** | 1200 | 0.911 | 6 | 0.000102 | 0.0132 |
| **Transcription factor binding** | 509 | 0.387 | 4 | 0.000409 | 0.0397 |
| Steroid hormone receptor activity | 56 | 0.0425 | 2 | 0.00079 | 0.0529 |
| Ligand_activated sequence_specific DNA binding RNA polymerase II transcription factor activity | 57 | 0.0433 | 2 | 0.000818 | 0.0529 |
| Single_stranded DNA binding | 65 | 0.0494 | 2 | 0.00106 | 0.0589 |
| Positive regulation of transcription, DNA_dependent | 1260 | 0.956 | 5 | 0.00145 | 0.0692 |
| Sequence_specific DNA binding | 732 | 0.556 | 4 | 0.00161 | 0.0692 |
| Lipid binding | 788 | 0.599 | 4 | 0.00211 | 0.0819 |
| Protein heterodimerization activity | 374 | 0.284 | 3 | 0.00242 | 0.0852 |
| Phosphatase binding | 116 | 0.0881 | 2 | 0.00334 | 0.108 |
| Double_stranded DNA binding | 149 | 0.113 | 2 | 0.00544 | 0.162 |
| Protein homodimerization activity | 573 | 0.435 | 3 | 0.00802 | 0.222 |
| DNA binding | 2760 | 2.1 | 6 | 0.00909 | 0.235 |
| Monocarboxylic acid transmembrane transporter activity | 36 | 0.0273 | 1 | 0.027 | 0.637 |
| Identical protein binding | 910 | 0.691 | 3 | 0.0279 | 0.637 |
| Protein dimerization activity | 996 | 0.757 | 3 | 0.0353 | 0.71 |
| Kinase binding | 418 | 0.318 | 2 | 0.0385 | 0.71 |

| | | | | | |
|---|---|---|---|---|---|
| Histone acetyltransferase activity | 52 | 0.0395 | 1 | 0.0388 | 0.71 |
| Transport | 4830 | 3.67 | 7 | 0.0389 | 0.71 |
| Solute:sodium symporter activity | 54 | 0.041 | 1 | 0.0403 | 0.71 |
| Transcription from RNA polymerase II promoter | 1930 | 1.47 | 4 | 0.0478 | 0.781 |
| Histone deacetylase binding | 65 | 0.0494 | 1 | 0.0483 | 0.781 |
| **GO CC** | | | | | |
| **Cellular Component** | **Total** | **Expected** | **Hits** | **P.Value** | **FDR** |
| Nucleoplasm | 1820 | 1.31 | 6 | 0.000876 | 0.197 |
| Nuclear part | 3330 | 2.4 | 7 | 0.00388 | 0.436 |
| Nuclear lumen | 2690 | 1.94 | 6 | 0.00673 | 0.463 |
| Nucleus | 7600 | 5.47 | 10 | 0.00885 | 0.463 |
| Nuclear chromosome part | 273 | 0.197 | 2 | 0.0158 | 0.463 |
| Chromosome | 784 | 0.564 | 3 | 0.0166 | 0.463 |
| Nuclear ubiquitin ligase complex | 26 | 0.0187 | 1 | 0.0186 | 0.463 |
| Nuclear replication fork | 28 | 0.0202 | 1 | 0.02 | 0.463 |
| Organelle lumen | 3380 | 2.44 | 6 | 0.0208 | 0.463 |
| Nuclear chromosome | 320 | 0.23 | 2 | 0.0214 | 0.463 |
| Membrane_enclosed lumen | 3440 | 2.48 | 6 | 0.0226 | 0.463 |
| Replication fork | 50 | 0.036 | 1 | 0.0354 | 0.664 |

**Supplementary Table 15.** List of enriched pathways, biological processes, molecular functions and cellular components in subnetwork 2/module 2 (pvalue 0.047, **Figure 4C**) of the network constructed upon the genes associated with the differentially methylated hyo- and hypermethylated promoters between the non-diseased aortic and mitral valve tissue based on KEGG/Reactome, Gene Ontology (GO) Biological Processes database, Gene Ontology (GO) Molecular Functions database and Gene Ontology (GO) Cellular Components database (full list). “Total” refers to the total number of genes that belong to a particular category as per the chosen reference list of genes, which is hg19 in our case, “Expected” denotes the number of genes to be expected in our gene list for a particular category, “Hits” describes the number of genes in our list that map to a particular category, “P.value” is equivalent to the enrichment p-value computed using the hypergeometric test (**see Methods**) and FDR stands for false discovery rate, which is the method used to correct the corresponding p-value for multiple testing.

## 3 References


1. Akalin A, Kormaksson M, Li S, Garrett-Bakelman FE, Figueroa ME, Melnick A, Mason CE. methylKit: a comprehensive R package for the analysis of genome-wide DNA methylation profiles. *Genome Biol* (2012) 13:R87. doi:10.1186/gb-2012-13-10-R87

2. Xia J, Benner M, Hancock R. NetworkAnalyst - integrative approaches for protein–protein interaction network analysis and visual exploration. *Nucleic Acids Res* (2014) 42:W167–W174. doi:10.1093/nar/gku443.
